# Supplementary material for: Chest radiograph findings in children aged 2–59 months hospitalised with community-acquired pneumonia, prior to the introduction of pneumococcal conjugate vaccine in India: a prospective multisite observational study
Source: BMJ Open. 2020 May 7;10(5):e034066. doi: 10.1136/bmjopen-2019-034066 (PMC7228527; doi:10.1136/bmjopen-2019-034066)
Supplement: Supplementary data [file bmjopen-2019-034066supp001.pdf]

Childhood Pneumonia Surveillance

Department of Paediatrics, KGMU, Lucknow ,UP

Form-R

(RADIOLOGY REPORT FORM)

1	**Drs_ID**	[__][__][__][__]
	**Radiology Report**	**Patient Details**
2	**IDNo:**	[__] / [__] / [__][__][__]/[__][__][__][__]<br>State /District / Unit / Subject number<br>(*For office use*)
2	Date Of Report	[__][__]/[__][__]/[__][__][__][__] (DD/MM/YYYY)
	**Report Details**	**Findings (tick one)**
3	Image Quality	Adequate ☐ Suboptimal ☐ Un-interpretable ☐
4	Significant Pathology	Yes ☐ No ☐ Un-interpretable ☐
5	**End Point Consolidation**	
5a	Left	Yes ☐ No ☐ Un-interpretable ☐
5b	Right	Yes ☐ No ☐ Un-interpretable ☐<br>Uninterpretable
6	Other Infiltrates/Abnormalities	
6a	Left	Yes ☐ No ☐ Un-interpretable ☐
6b	Right	Yes ☐ No ☐ Un-interpretable ☐
7	Pleural Fluid	
7a	Left	Yes ☐ No ☐ Un-interpretable ☐
7b	Right	Yes ☐ No ☐ Un-interpretable ☐
8	Comments:	
9	Conclusion:	a) Primary endpoint pneumonia only ☐<br>b) Other infiltrate only ☐<br>c) Both PEP and other infiltrate ☐<br>d) Normal ☐<br>e) Un-interpretable for any findings☐
