# Supplementary material for: Chest radiograph findings in children aged 2–59 months hospitalised with community-acquired pneumonia, prior to the introduction of pneumococcal conjugate vaccine in India: a prospective multisite observational study
Source: BMJ Open. 2020 May 7;10(5):e034066. doi: 10.1136/bmjopen-2019-034066 (PMC7228527; doi:10.1136/bmjopen-2019-034066)
Supplement: Author's manuscript [file bmjopen-2019-034066.draft_revisions.pdf]

BMJ Open is committed to open peer review. As part of this commitment we make the peer review history of every article we publish publicly available.

When an article is published we post the peer reviewers' comments and the authors' responses online. We also post the versions of the paper that were used during peer review. These are the versions that the peer review comments apply to.

The versions of the paper that follow are the versions that were submitted during the peer review process. They are not the versions of record or the final published versions. They should not be cited or distributed as the published version of this manuscript.

BMJ Open is an open access journal and the full, final, typeset and author-corrected version of record of the manuscript is available on our site with no access controls, subscription charges or pay-per-view fees (http://bmjopen.bmj.com).

If you have any questions on BMJ Open's open peer review process please email info.bmjopen@bmj.com

BMJ Open

Chest Radiograph Findings in children aged 2-59 months hospitalized with Community-Acquired Pneumonia, prior to the introduction of Pneumococcal Conjugate Vaccine in India

Journal:	*BMJ Open*
Manuscript ID	bmjopen-2019-034066
Article Type:	Original research
Date Submitted by the Author:	05-Sep-2019

Complete List of Authors:	Awasthi, Shally; King George's Medical University, Pediatrics<br>Rastogi, Tuhina; King George's Medical University, Paediatrics<br>Mishra, Neha; King George's Medical University, Pediatrics<br>Chauhan, Abhishek ; Dr Ram Manohar Lohia Institute of Medical Sciences, Department of Radio-diagnosis<br>Mohindra, Namita; Sanjay Gandhi Post Graduate Institute of Medical Sciences, Department of Radio-diagnosis<br>Shukla, Ram ; Banaras Hindu University, Varanasi, India, Institute of Medical Sciences<br>Agarwal, Monica; King George's Medical University, Department of Community Medicine<br>Pandey, CM; Sanjay Gandhi Post Graduate Institute of Medical Sciences, Biostatistics & Health Informatics<br>Kohli, Neera ; King George's Medical University, Department of Radio-diagnosis<br>Study Group, CAP; CAP study Group

Keywords:	Chest radiographs, Hospitalized community-acquired pneumonia, under-five, Streptococcus pneumoniae, India

SCHOLARONE™
Manuscripts

I, the Submitting Author has the right to grant and does grant on behalf of all authors of the Work (as defined in the below author licence), an exclusive licence and/or a non-exclusive licence for contributions from authors who are: i) UK Crown employees; ii) where BMJ has agreed a CC-BY licence shall apply, and/or iii) in accordance with the terms applicable for US Federal Government officers or employees acting as part of their official duties; on a worldwide, perpetual, irrevocable, royalty-free basis to BMJ Publishing Group Ltd ("BMJ") its licensees and where the relevant Journal is co-owned by BMJ to the co-owners of the Journal, to publish the Work in this journal and any other BMJ products and to exploit all rights, as set out in our licence.

The Submitting Author accepts and understands that any supply made under these terms is made by BMJ to the Submitting Author unless you are acting as an employee on behalf of your employer or a postgraduate student of an affiliated institution which is paying any applicable article publishing charge ("APC") for Open Access articles. Where the Submitting Author wishes to make the Work available on an Open Access basis (and intends to pay the relevant APC), the terms of reuse of such Open Access shall be governed by a Creative Commons licence – details of these licences and which Creative Commons licence will apply to this Work are set out in our licence referred to above.

Other than as permitted in any relevant BMJ Author's Self Archiving Policies, I confirm this Work has not been accepted for publication elsewhere, is not being considered for publication elsewhere and does not duplicate material already published. I confirm all authors consent to publication of this Work and authorise the granting of this licence.

For peer review only

Title: *Chest Radiograph Findings in children aged 2-59 months hospitalized with Community-Acquired Pneumonia, prior to the introduction of Pneumococcal Conjugate Vaccine in India*

Short Title: *Radiological Findings in Children hospitalized with Community-Acquired Pneumonia in India Pre-Pneumococcal Conjugate Vaccine introduction*

Shally Awasthi[1]*, Tuhina Rastogi[1], Neha Mishra[1], Abhishek Chauhan[2], Namita Mohindra[3], Ram Chandra Shukla[4], Monica Agarwal[5], Chandra Mani Pandey[6], Neera Kohli[7] and CAP Group^

[1] Department of Pediatrics, King George's Medical University, Lucknow, India

[2] Department of Radio-diagnosis, Dr Ram Manohar Lohia Institute of Medical Sciences, Lucknow, India

[3] Department of Radio-diagnosis, Sanjay Gandhi Post Graduate Institute of Medical Sciences, Lucknow, India

[4]Department of Radio-diagnosis, Institute of Medical Sciences, Banaras Hindu University, Varanasi, India

[5] Department of Community Medicine, King George's Medical University, Lucknow, India

[6]Department of Biostatistics and Health Informatics, Sanjay Gandhi Postgraduate Institute of Medical Sciences, Lucknow, India

[7]Department of Radio-diagnosis, King George's Medical University, Lucknow, India

^ Members of the Lucknow CAP Group are given in the Acknowledgement

*Corresponding Author

Postal Address: Prof. Shally Awasthi, Head, Department of Paediatrics, King George's Medical University, Lucknow , India Pincode: 226003

Mobile: +91-9839221244

E-mail: shally07@gmail.com

Word Count: 3587 Tables: 3 Figure: 1

For peer review only - http://bmjopen.bmj.com/site/about/guidelines.xhtml

ABSTRACT

Objectives: To assess radiological abnormalities in chest X-rays and to identify the demographic and clinical correlates in children aged 2-59 months, hospitalized with World Health Organization defined community-acquired pneumonia, who reside in pre-specified districts of India

Design: Prospective, hospital-based surveillance

Setting: Multi-site study conducted in a network of 117 secondary/tertiary care hospitals in four districts of Uttar Pradesh and Bihar, India.

Participants: Included were children aged 2-59 months hospitalized with community-acquired pneumonia, residing in project district, with duration of illness of <14 days and who were not hospitalized elsewhere for this episode or nor had been recruited previously.

Main outcome measure: Radiological abnormalities in the chest X-rays, where there was concordance between two or more of the panel of three trained radiologists.

Results: From January 2015 to April 2017, 3214 cases were recruited and in 99.40 % (3195/3214) chest X-rays were available. Among 88.54 % (2829/3195) interpretable X-rays, 34.53 % (977/ 2829, 95% C.I. 32.78 - 36.28) had some radiological abnormalities, while the rest were normal. Primary end point pneumonia alone or with other infiltrates was found in 22.44 % (635/2829, C.I. 20.90 %-23.98 %), other infiltrates only in 12.09% (342/ 2829; C.I. 10.88 %-13.29 %). There was a statistically significant inter-district variation in radiological abnormality. Statistically significantly higher proportion of abnormal chest X-ray was found among girls, those with weight for age z score ≤-3 SD, longer duration of fever, pallor and with exposure to biomass fuel.

For peer review only - http://bmjopen.bmj.com/site/about/guidelines.xhtml

For peer review only

Conclusions: Among hospitalized cases of community-acquired pneumonia, almost one-third children had abnormal chest radiographs of which about two-thirds had abnormalities related with possible bacterial etiology (*Streptococcus pneumoniae*). Hence introduction of pneumococcal vaccination is likely to reduce burden of childhood pneumonia in India.

Key words: Chest radiographs, Hospitalized community-acquired pneumonia, under-five, *Streptococcus pneumoniae*, India

Strengths and Limitations of the Study

- Prospective, multisite recruitments from a large hospital surveillance network established for the project in four districts in two states of India that have high under-five mortality rates
- Standardized World Health Organization definition was used for identifying hospitalized cases of clinical pneumonia
- Radiological abnormalities interpreted by a panel of three trained radiologists at locations out of the surveillance network, blinded to each other as well as clinical features of the case
- Since pre-existing X-rays machines were used, there was variation in the quality of images obtained, which were minimized by digitizing them centrally
- Laboratory investigations were according to the routine hospital practice and were not uniform across hospitals, since the study objective was to assess radiological abnormalities in chest X-rays of recruited cases .

For peer review only - http://bmjopen.bmj.com/site/about/guidelines.xhtml

INTRODUCTION

Community-acquired pneumonia (CAP) is the single largest infectious cause of death in young children worldwide. Globally, pneumonia accounts for 16% of deaths in children under-five years of age and results in almost one million deaths (0.9 million children in 2016) every year [1 2]. Most deaths due to pneumonia occur in low and middle income countries particularly in sub-Saharan Africa and South Asia [2 3]. In India, there were approximately 0·44 million under-five deaths due to CAP in the year 2015 [4].

CAP could have viral or bacterial etiology [5 - 7]. Etiology varies from country to country and also across different time periods. Pediatric bacterial pneumonia is predominantly caused by *Streptococcus pneumoniae* (*SP*) and *Hemophilus influenzae Type B* (HiB) while Respiratory syncytial virus and Influenza A or B virus are important contributors of pediatric viral pneumonia [5 6]. The World Health Organization (WHO) recommends the introduction of Pneumococcal Conjugate Vaccine (PCV) in the national immunization programme (NIP) of countries with high child mortality rates, which includes India [8]. Consequently, PCV-13 was launched in May 2017 under the NIP of five Indian states (Uttar Pradesh, Bihar, Rajasthan, Madhya Pradesh and Himachal Pradesh) in a phased manner [9]. It is expected to be rolled out in other parts of the country in the coming days. Vaccination against HiB is already under the NIP since 2011.

Differentiating bacterial from viral etiology of CAP on clinical features or by investigations remains difficult [7 10 11]. Several PCV probe trials have used radiographically confirmed alveolar

For peer review only - http://bmjopen.bmj.com/site/about/guidelines.xhtml

pneumonia, also called end-point pneumonia, to be an outcome for vaccine efficacy and this has been endorsed by WHO [12-14].

The current study was a hospital-based surveillance to assess the radiological abnormalities in chest X-rays (CXRs) and to identify the demographic clinical correlates of specific radiological abnormalities in children aged 2-59 months, hospitalized with WHO defined CAP, residing in pre-specified districts of Uttar Pradesh and Bihar, India.

METHODS

Setting

This multi-site site prospective study was conducted in Lucknow and Etawah districts of Uttar Pradesh and Patna & Darbhanga districts of Bihar, India. Uttar Pradesh is the most populated state of India and Bihar third populated [15] [16]. Lucknow district has urban population of 66.2% [15] and Patna district 43.07% [16.] In contrast, only 22.3%[15] population of Etawah district and 9.74%[16] population of Darbhanga district reside in rural areas. All four project districts have poor socio-demographic and child health indicators [15 16 17].

Study Population

A hospital-based surveillance system was established for this study. Recruitment was done from a network of public and private hospitals, which provided either secondary or tertiary level care, which admitted children. Children aged 2-59 months hospitalized in network hospitals with history of fast breathing with/without chest in-drawing were screened [17].

For peer review only - http://bmjopen.bmj.com/site/about/guidelines.xhtml

For peer review only

Children with cough and respiratory symptoms for ≥ 14 days were excluded [17]. Children with history of hospitalization within 14 days of recruitment were excluded to remove the potential risk of acquiring hospital-acquired pneumonia [17]. Included were children hospitalized with symptoms of WHO defined CAP and residing in the project district. WHO has developed guidelines for the identification of CAP by the community health workers [18.] According to these guidelines, CAP is defined as the presence of fast breathing above age-specific cutoff. The cutoff for infants less than 2 months is 60 more breaths per minute (bpm), for 2-11 months of age 50 or more bpm and 12-59 months of age is 40 or more bpm [18]. In addition, WHO has defined severe pneumonia as CAP with presence of certain danger signs such as not able to drink, persistent vomiting, convulsions, lethargy or unconsciousness, stridor in a calm child or severe malnutrition [18]. Children with fast breathing with or without chest in-drawing are classified as "pneumonia" and children with pneumonia and with any danger signs are classified as "severe pneumonia" [18].

Data collection

Information on socio-demographic and clinical variables was obtained by trained surveillance officers. Socio-demographic information was collected through face-to-face interviews from the parent/caregiver of the recruited child. Clinical data and anthropometric information (height, weight, mid-arm circumference and head circumference) was abstracted from clinical records of admitting hospital. Clinical Outcome (survival or mortality) was noted [17 19].

Chest x-ray (CXR) image acquisition and archiving

CXR (poster-anterior view) was done on the advice of treating physician as part of routine clinical care. Surveillance staff obtained CXR at the time of recruitment. CXRs were either analogous or digital. In case of digital CXRs, second copy was obtained where possible. If only

For peer review only - http://bmjopen.bmj.com/site/about/guidelines.xhtml

single analog image was available, then the CXR hardcopy was obtained from the parent/guardian after the child was discharged. If this could not be done, image of the hardcopy was captured. CXR machines were not provided through the project.

CXRs of recruited cases were subsequently scanned and converted into digital format using a diagnostic-quality film image digitalizer (Microteck International Limited, model Medi 6000 plus) [20]. CXRs obtained/converted into digital image were stored as per the standard operating procedure and were subsequently archived for web-based radiological interpretation. Digital images were stored in JPEG format at 300 dpi resolution. Each CXR file was anonymized and given a unique identification number. Digital CXRs were uploaded on online data management software (www.capxrs.org), developed especially for the project. De-identified CXRs were uploaded every month in batches by the data manager.

Interpretation of radiological images

A panel of radiologists was constituted for standardized interpretations of pediatric CXRs. Four radiologists were part of this panel, one of whom was Project Co-Investigator-Radiology. Radiologists were trained as per the methodology developed by Department of Immunization, Vaccines, and Biologicals of the World Health Organization (WHO) [11].

After training, radiologists were required to independently review CXRs and register their findings in an online standardized chest radiograph interpretation form **[S1 Appendix].** For optimal viewing of CXRs, all readers used similar workstations. Specifications were provided for the computer monitor and hardware to be used. It was ensured that monitors had the correct

For peer review only - http://bmjopen.bmj.com/site/about/guidelines.xhtml

brightness and contrast adjustment for optimal viewing. The sequence of presentation of CXR to the readers was randomized.

Radiographic interpretation was done on film quality, findings of CXRs and conclusion. Radiologists interpreted film quality as follows: (a) `*Adequate/optimal*` for features allow confident interpretation of consolidation and pleural effusion as well as other infiltrates; (b) `*Suboptimal*` for features allow interpretation of consolidation and pleural effusion, but not of other infiltrates or findings and (c) `*Un-interpretable*` pertaining to features of the image that are not interpretable with respect to presence or absence of consolidation or pleural effusion without additional images [12].

After interpreting film quality, readers interpreted the pathological findings. For each radiographic finding, there were two options to be chosen: `yes` for the presence of findings and `no` for its absence. Pathological findings were classified into (a)`*significant pathology*` such as presence of consolidation, infiltrates or effusion; (b) `*end-point consolidation*` for CXRs with a dense or confluent opacity that occupies a portion or whole of a lobe or the entire lung, that may or may not contain air bronchograms ; (c) `*other (non end-point) infiltrate*` for CXRs with linear and patchy opacities (interstitial infiltrate) in a lacy pattern, featuring peri-bronchial thickening and multiple areas of atelectasis; also including minor patchy infiltrates that are not of sufficient magnitude to constitute endpoint consolidation, and small areas of atelectasis that in children may be difficult to distinguish from consolidation and (d) `*pleural effusion*` on presence of fluid in the lateral pleural space between the lung and chest wall that is spatially associated with a pulmonary parenchymal infiltrate (including `other infiltrate`) or has obliterated enough of the

For peer review only - http://bmjopen.bmj.com/site/about/guidelines.xhtml

hemithorax to obscure any infiltrate; in most cases, this will be seen at the costo-phrenic angle or as a layer of fluid adjacent to the lateral chest-wall; this does not include fluid seen in the horizontal or oblique fissures [12].

Radiologists concluded their interpretations of CXRs as per WHO guidelines [12]. Conclusions were categorised into: (a) `*Primary End Point Pneumonia only*` (PEP) on the presence of consolidation or pleural effusion; (b) `*Other (non end-point) infiltrate only*` on the presence of other (non-consolidation) infiltrates as defined above in the absence of a pleural effusion (c) `*Both PEP and other infiltrate*` and (d) `*Normal*` when there were no findings consistent with 'endpoint consolidation' or 'other infiltrate' or `pleural effusion` [12].

After radiological interpretation, online data was archived, stored and checked for inconsistencies and completeness by the data manager. CXRs with concordant and discordant interpretations were identified. Interpretations were considered concordant when two or more radiologists agreed on the same. If all the three radiologists disagreed on set of findings, then such CXRs with discordant interpretations were forwarded to the study arbitrator (Project Co-Investigator-Radiology) using customized software (www.capxrs.org). Arbitrator read discordant CXRs and submitted the interpretation to the data manager. Readings of arbitrator were taken as final in case of discordant interpretations.

Data management and statistical analysis

Clinical data of hospital surveillance network was entered online in customized software. Primary entry was by the four participating sites. Secondary data entry was done by the coordinating site in different customized software. Anonymized CXRs were uploaded on

For peer review only - http://bmjopen.bmj.com/site/about/guidelines.xhtml

customized software. Each of the three panelists had independent access to them. They assessed the CXRs online, blind to peer assessments as well as clinical features of the case, and uploaded their findings online. CXR assessment data was downloaded from the online software in MS Access database.

Exploratory data analysis was performed for outlier detection and missing observations for all the variables. Descriptive statistics was calculated for measurable variables in Mean ± Standard Deviation (M±SD) and categorical variables in percent (%). Un-interpretable CXRs were removed from analysis. Among interpretable CXRs, radiological abnormalities, which were reported by two members of the panel, were taken as final. Weight-for-age z-score each child was calculated using Epi-Info software [21]. Weight-for-age z score (WAZ) of ≤ -3 was taken as `underweight` [22]. Kappa statistics was performed for agreement analysis among radiologists for CXRs findings. Statistical analysis was performed using SPSS version 22.0 (Chicago, IL) [23]. A p value of <0·05 was taken as statistically significant using a two-tailed distribution.

Univariate analysis was performed to evaluate heterogeneity stratified by four participating sites for socio-demographic variables such as age, gender, place of residence, type of house, type of family, maternal & paternal education and their occupation, use of biomass fuel for cooking and parental smoking status. Likewise, univariate analysis was done for clinical variables such as height, weight, duration of illness and percent oxygen saturation, in cases where pulse oximetry was done.

For peer review only - http://bmjopen.bmj.com/site/about/guidelines.xhtml

We report proportions of radiological abnormalities among hospitalized children for CAP for four districts. Univariate analysis was performed to find out associated socio-demographic variables and clinical signs of CAP with radiological abnormalities. Chi-square test was used to find out association for categorical variables and student's t-test for continuous variables. Multivariate binary logistic regression was performed find association of presence of various radiological abnormalities among cases hospitalized for CAP, controlling for district of residence and other variables that had univariate association with radiological abnormalities (p value ≤ 0.2) and/or were clinically meaningful.

Thereafter, we developed and report four models for estimation of adjusted odds ratios of socio-demographic and clinical variables with specific radiological abnormalities (dependent variable). In these four models, dependent (outcome) were as follows:

Model I: Abnormal vs. Normal

Model II: Primary End Point Pneumonia alone or with infiltrates vs. Normal

Model III: Primary End Point Pneumonia alone vs. Normal

Model IV**:** Other infiltrates only vs. Normal

Independent variables that were kept across all the four models were : participating districts, age, gender, use of biomass fuel, symptoms of CAP such as duration of illness, presence of rhonchi, pallor and vomiting and malnutrition status of the case [WAZ $\leq$ -2 (malnourished) and WAZ $\leq$ -3 (severely malnourished)].

Patient and public involvement in research

For peer review only - http://bmjopen.bmj.com/site/about/guidelines.xhtml

Health Ministry Steering Committee of Indian Council of Medical Research, New Delhi, India approved the study. State governments of Uttar Pradesh and Bihar gave consent for initiating the study. Written, informed consent was obtained from parents/guardians of eligible children who were willing to participate in the study. Written informed consent was also taken from the administration of hospital for participation.

RESULTS

A total of 3290 hospitalized cases were screened in hospital surveillance network of Lucknow and Etawah districts of Uttar Pradesh and Patna & Darbhanga districts of Bihar from January 2015 to April 2017. Out of these, 3214 cases fulfilling the WHO diagnosis of CAP were included **[Figure 1].** Among them, 3195 (99.40%) cases were enrolled with CXRs and in 19 (1.0%) cases CXRs were not done. Concordance among ≥ 2 radiologists for CXRs findings was 86.0%. Kappa statistics was calculated for agreement of CXRs findings between Reader 1 vs. Reader 2 (K_1=0.31), Reader 2 vs. Reader 3 (K_2=0.46) and Reader 3 vs. Reader 1(K_3=0.42). Thereafter, out of these 88.54% (2829/3195) CXRs were found interpretable and remaining 11.45% (366/3195) were found un-interpretable by radiologists. Among interpretable CXRs, we found 22.44% (635/2829) children had primary end point pneumonia (PEP) alone or with infiltrates, other infiltrates only 12.09% (342/2829) and 65.46% (1852/2829) had normal CXRs findings [**Figure 1**].

Table 1 shows univariate distribution of socio-demographic and clinical variables among hospitalized cases across four participating districts. A statistically significant variation was observed in all socio-demographic variables such as place of residence, type of house, type of

For peer review only - http://bmjopen.bmj.com/site/about/guidelines.xhtml

family, maternal and paternal education and occupation, use of biomass fuel and parental smoking status across the four districts. We also found and report clinical variables of recruited cases across the four districts in **table 1**. While oxygen saturation by pulse-oxymetry was statistically significantly different across the sites, the proportion of cases with oxygen saturation ≤92 % was found not significant in the children across four districts (p=0.13).

Table 1: Distribution of socio-demographic and clinical variables among hospitalized children for participating districts (Jan 2015-April 2017)

**Characteristics**	**Lucknow**	**Etawah**	**Patna**	**Darbhanga**	**Total**	
**Socio-demographic Characteristics**	**n=1025 (%)**	**n=389 (%)**	**n=744 (%)**	**n=671 (%)**	**N=2829 (%)**	**p value**
**Gender**						
Male	659(64.29)	287(73.78)	557(74.87)	502(74.81)	2005(70.87)	<0.0001
**Place of residence**						
Rural	195(19.02)	279(71.72)	304(40.86)	614(91.51)	1392(49.20)	<0.0001
Urban	830(80.98)	110(28.28)	440(59.14)	57(8.49)	1437(50.80)	
**Family Type**						
Joint	688(67.12)	360(92.54)	707(95.03)	383(57.08)	2138(75.57)	<0.0001
Nuclear	337(32.88)	29(7.46)	37(4.97)	287(42.77)	690(24.39)	
**House type**						
Mud	64(6.24)	54(13.88)	123(16.53)	374(55.74)	615(21.74)	
Bricks	854(83.32)	256(65.81)	453(60.89)	85(12.67)	1648(58.25)	<0.0001
Combined	107(10.4)	79(20.31)	168(22.58)	212(31.59)	566(20.01)	
**Mother's Education**						
No formal education	203(19.80)	56(14.40)	328(44.09)	496(73.92)	1083(38.28)	
Class I-V	108(10.54)	28(7.20)	82(11.02)	38(5.66)	256(9.05)	<0.0001
Class VI-XII	379(36.98)	176(45.24)	243(33.66)	112(16.69)	910(32.17)	
Graduate/ Post graduation	335(32.68)	129(33.16)	91(12.23)	25(3.73)	580(20.50)	
**Father's Education**						
No formal education	167(16.29)	29(7.46)	153(20.56)	345(51.42)	694(24.53)	
Class I-V	85(8.29)	19(4.88)	91(12.23)	82(12.22)	277(9.79)	
Class VI-XII	437(42.63)	206(52.96)	328(44.09)	205(30.55)	1176(41.57)	<0.0001
Graduate/ Post graduation	336(32.78)	135(34.70)	172(23.12)	39(5.81)	682(24.11)	
**Birth Order**						
0	435(42.44)	187(48.07)	315(42.34)	192(28.61)	1129(39.91)	
1st	343(33.46)	120(30.85)	235(31.59)	258(38.45)	956(33.79)	<0.0001
2nd	153(14.93)	47(12.08)	129(17.34)	137(20.42)	466(16.47)	
More than 2nd	93(9.07)	35(9.00)	62(8.33)	83(12.37)	273(9.65)	
**Immunization Status**						
Complete for age	792(77.27)	300(77.12)	711(95.56)	544(81.07)	2347(82.96)	
Incomplete for age	220(21.46)	84(21.59)	25(3.36)	126(18.78)	455(16.08)	<0.0001

For peer review only - http://bmjopen.bmj.com/site/about/guidelines.xhtml

For peer review only

Unimmunized	13(1.27)	5(1.29)	8(1.08)	1(0.15)	27(0.95)	
**Currently Breast Feeding**						
Yes	653(63.71)	256(65.81)	589(79.17)	537(80.03)	2035(71.93)	<0.0001
No	372(36.29)	133(34.19)	155(20.83)	134(19.97)	794(28.07)	
**Father's Occupation**						
Unemployed	13(1.27)	20(5.14)	27(3.63)	63(9.39)	123(4.35)	
Daily wages	329(32.10)	81(20.82)	165(22.18)	474(70.64)	1049(37.08)	
Salaried/ Professional	397(38.73)	104(26.74)	245(32.93)	55(8.20)	801(28.31)	<0.0001
Self-Employment	286(27.90)	184(47.30)	307(41.26)	79(11.77)	856(30.26)	
**Mother's Occupation**						
Home maker	961(93.76)	376(96.66)	701(94.22)	484(72.13)	2522(89.15)	
Daily wages	17(1.66)	3(0.77)	17(2.28)	171(25.48)	208(7.35)	
Salaried/Professionals	47(4.59)	9(2.31)	18(2.42)	7(1.04)	81(2.86)	<0.0001
Self-Employment	0(0.0)	1(0.26)	8(1.08)	9(1.34)	18(0.64)	
**Biomass fuel**						
Yes	211(20.59)	245(62.98)	263(35.35)	609(90.76)	1328(46.94)	<0.0001
No	814(79.41)	144(37.02)	481(64.65)	62(9.24)	1501(53.06)	
**Smoking Status-Father**						
Yes	152(14.83)	45(11.57)	56(7.53)	59(8.79)	312(11.03)	<0.0001
No	873(85.17)	344(88.43)	688(92.47)	612(91.21)	2517(88.97)	
**Indoor smoking-Father**						
Yes	83(8.10)	21(5.40)	16(2.15)	43(6.41)	163(5.76)	<0.0001
No	942(91.90)	368(91.60)	728(97.85)	628(93.59)	2666(94.24)	
**Smoking Status-Family member**						
Yes	129(12.59)	55(14.14)	45(6.05)	102(15.20)	331(11.70)	<0.0001
No	896(87.41)	334(85.86)	699(93.95)	569(84.80)	2498(83.30)	
**Indoor smoking – Family member**						
Yes	84(8.20)	27(6.94)	27(3.63)	94(14.01)	232(8.20)	<0.0001
No	941(91.80)	362(93.06)	717(96.37)	577(85.99)	2597(91.80)	
**Clinical Variables at the time of admission at hospital**	**n, Mean± SD**	**n, Mean± SD**	**n, Mean± SD**	**n, Mean± SD**	**n, Mean± SD**	**p value**
Age (months)	1025, 14.53±13.88	389, 10.69±10.95	744, 10.26±11.35	671, 12.30±13.29	2829, 12.35±12.85	<0.0001
Height (cm)	303, 68.61±13.78	324, 70.66±13.75	34, 64.38±10.25	266, 70.46±12.14	927, 69.70±13.26	<0.018
Weight (Kg)	900, 7.89±3.02	387, 7.35±2.74	682, 7.07±2.79	643, 7.70±2.88	2612, 7.55±2.90	<0.0001
Fever Duration (days)	929, 4.46±2.71	321, 3.59±2.37	689, 4.25±2.52	569, 3.54±2.47	2508, 4.08±2.59	<0.0001
Oxygen saturation (%)	528, 93.68±5.56	343, 92.56±6.20	236, 92.23±5.28	319, 94.19±4.63	1426, 93.28±5.53	<0.0001
≤92 Oxygen Saturation Value	179, 88.26±6.37	132, 87.12±6.94	122, 88.57±4.69	70, 87.1±4.17	503, 87.87±5.98	0.13

For peer review only - http://bmjopen.bmj.com/site/about/guidelines.xhtml

Table 2 shows proportions of radiological pneumonia among cases hospitalized for CAP in four participating districts. We found higher proportion of radiological abnormalities in Patna district [38.58 (95% CI: 35.07-42.07)] and Lucknow district [37.95 (95%CI: 34.98-40.92)] which have a large and urban population. Lower proportion of radiological abnormalities were noted in Etawah district [29.31 (95%CI: 24.78-33.82)] and Darbhanga district [27.87 (95% CI: 24.47-31.26)] which in contrast had larger rural population. We also observed correspondingly higher proportion of PEP alone or with other infiltrates in districts of Lucknow [72.49 (95% CI: 68.05-76.93)]; and Patna [64.11 (95% CI: 58.56-69.66)] and lower in districts of Etawah [64.04 (95% CI: 55.22 -72.84)]; and Darbhanga [51.34 (95% CI: 44.17-58.50)].

Table 2 also describes univariate distribution of socio-demographic and clinical factors of CAP among hospitalized children aged 2-59 months. We observed statistically significant district-wise heterogeneity in radiological abnormalities. Statistically significantly higher proportion of females hospitalized for CAP had radiologically abnormal CXR. Likewise, statistically significantly higher proportion of abnormal vs. normal CXRs findings were reported in hospitalized cases who had symptoms of fever, pallor rhonchi and vomiting or were malnourished.

Table 2: Distribution of socio-demographic and clinical factors by chest radiograph findings among hospitalized children from January 2015-April 2017

Socio-demographic & clinical factors	Interpretable chest X rays				Abnormal chest X rays		
	N=2829	Normal 1852 n (%)	Abnormal 977 n (%)	p value	PEP* alone or with other infiltrate 635 n (%)	Other infiltrates 342 n (%)	p value
**Participating site**							
Lucknow	1025	636 (62.05)	389 (37.95)		282 (72.49)	107 (27.51)	

For peer review only - http://bmjopen.bmj.com/site/about/guidelines.xhtml

Etawah	389	275 (70.69)	114 (29.31)	<0.0001	73 (64.04)	41 (35.96)	<0.0001
Patna	744	457 (61.42)	287 (38.58)		184 (64.11)	103 (35.89)	
Darbhanga	671	484 (72.13)	187 (27.87)		96 (51.34)	91 (48.66)	
**Age-group (months)**							
2-11	1865	1223 (65.58)	642 (34.42)	0.86	409 (63.71)	233 (36.29)	0.26
12-59	964	629 (65.25)	335 (34.75)		226 (67.46)	109 (32.54)	
**Gender**							
Male	2005	1354 (67.53)	651 (32.46)	<0.0001	426 (65.44)	225 (34.56)	
Female	824	498 (60.43)	326 (39.56)		209 (64.44)	117 (35.89)	0.72
**Place of residence**							
Rural	1392	921 (66.16)	471 (33.83)	0.44	299 (63.48)	172 (36.52)	
Urban	1437	931 (64.78)	506 (35.21)		336 (66.40)	170 (33.60)	0.34
**Biomass fuel**							
**Yes**	1501	867 (65.29)	461 (34.71)	0.44	294(63.77)	167 (36.23)	0.24
**No**	1328	985 (65.62)	516 (34.38)		341 (66.09)	175 (33.91)	
**Immunization status**							
Complete for age	2347	1546 (68.87)	801 (34.12)	0.32	516 (64.42)	285 (35.58)	0.54
Incomplete	482	306 (63.48)	176 (36.51)		119 (67.61)	57 (32.39)	
**Symptoms of pneumonia**							
Fever	2499	1616 (64.66)	883 (35.33)	0.014	575 (65.12)	308 (34.88)	0.82
Cyanosis	62	39 (62.90)	23 (37.09)	0.34	16 (69.57)	7 (30.43)	0.64
Pallor	764	465 (60.86)	299 (39.13)	0.002	200 (66.89)	99 (33.11)	0.41
Rhonchi	2054	1377 (67.03)	677 (32.96)	0.005	415 (61.30)	262 (38.70)	0.0003
**Duration of illness fever in**	2508	3.91±2.51	4.40±2.70	<0.0001	4.57±2.82	4.08±2.44	0.011

For peer review only - http://bmjopen.bmj.com/site/about/guidelines.xhtml

**days (Mean ± SD)**							
**Danger Signs of pneumonia**							
Vomiting	899	605 (67.30)	294 (32.70)	0.17	174 (59.18)	120 (40.82)	0.01
Lethargy	1101	732 (66.49)	369 (33.51)	0.39	247 (66.94)	122 (33.06)	0.33
Difficulty in breathing	2705	1766 (65.29)	939 (34.71)	0.39	609 (64.86)	330 (35.14)	073
Inability to drink	937	612 (65.31)	325 (34.69)	0.46	211 (64.92)	114 (35.08)	0.97
Convulsion	148	98 (66.22)	50 (33.78)	0.93	33 (66.0)	17 (34.0)	0.87
Blue Lips	42	27 (64.29)	15 (35.71)	0.87	12 (80.0)	3 (20.0)	0.28
**Malnutrition Status**							
Normal *	1912	1312 (68.62)	600 (31.38)	< 0.0001	374 (62.33)	226 (37.67)	0.06
Malnutrition*	485	314 (64.74)	171 (35.26)		115 (67.25)	56 (32.75)	
Severe malnutrition*	432	226 (52.31)	206 (47.69)		146 (70.87)	60 (29.13)	

*Normal-weight of age z score> -2SD; Malnutrition-weight of age z ≤ -2SD and Severe malnutrition-weight of age z ≤ -3SD

Table 3 describes four multivariate logistic regression models to find associates of abnormal CXR findings. After controlling for age, gender, symptoms of pneumonia, duration of illness, biomass fuel and malnutrition status of cases, statistically significant district-wise heterogeneity remained in the first three models. Models I, II and III had similar associates for radiological abnormalities whereas Model IV was different. Across all the four models, female cases of CAP and those who had severe malnutrition had statistically significantly higher risk for having abnormal CXRs. A higher risk of radiological abnormalities was also observed in those children who had longer duration of illness.

For peer review only - http://bmjopen.bmj.com/site/about/guidelines.xhtml

Table 3: Independent Associations between Chest Radiograph Findings and demographic and clinical factors, among hospitalized children January 2015-April 2017

Variables	Model – I Abnormal/Normal Ref		Model – II PEP alone or with other infiltrate /Normal Ref		Model – III PEP alone Vs. Normal Ref		Model – IV Other infiltrate / Normal Ref	
	Adjusted Odd Ratio (95%CI)	p value	Adjusted Odd Ratio (95%CI)	p value	Adjusted Odd Ratio (95%CI)	p value	Adjusted Odd Ratio (95%CI)	p value
**Districts**								
Lucknow Vs. Others	1.58 (1.20-2.10)	<0.0001	2.07 (1.48-2.89)	<0.0001	2.20 (1.52-3.19)	<0.0001	0.98 (0.65-1.47)	0.93
Etawah Vs. Others	1.22 (0.88-1.70)	0.23	1.30 (0.87-1.95)	0.19	1.49 (0.95-2.30)	0.07	1.17 (0.74-1.87)	0.50
Patna Vs. Others	1.67 (1.27-2.20)	<0.0001	1.89 (1.36-2.64)	<0.0001	2.25 (1.56-3.24)	<0.0001	1.39 (0.95-2.07)	0.09
**Age – Group (months)**								
2-11 Ref								
12-59	0.92 (0.77-1.10)	0.34	0.95 (0.77-1.17)	0.62	1.03 (0.82-1.29)	0.79	0.86 (0.66-1.13)	0.27
**Gender**								
Male Ref								
Female	1.39 (1.16-1.66)	<0.0001	1.34 (1.08-1.65)	0.008	1.28 (1.01-1.61)	0.03	1.48 (1.14-1.92)	0.004
**Symptoms of pneumonia¶**								
Rhonchi	0.83 (0.68-1.01)	0.06	0.72 (0.57-0.90)	0.005	0.75 (0.59-0.96)	0.02	1.14 (0.83-1.55)	0.42
Pallor	1.30 (1.08-1.58)	0.006	1.28 (1.03-1.60)	0.02	1.22 (0.95-1.55)	0.12	1.34 (1.01-1.77)	0.04
Vomiting	0.90 (0.75-1.09)	0.28	0.80 (0.64-0.99)	0.04	0.78 (0.62-1.01)	0.05	1.09 (0.83-1.08)	0.51
**Duration of illness, fever (days)**	1.06 (1.04-1.09)	<0.0001	1.08 (1.04-1.12)	<0.0001	1.08 (1.04-1.12)	<0.0001	1.03 (0.98-1.48)	0.24
**Biomass fuel**	1.28 (1.05-1.57)	0.02	1.39 (1.10-1.76)	0.006	1.40 (1.14-1.88)	0.003	1.08 (0.79-1.45)	0.64
**Malnutrition Status**								
Normal *Ref								
Malnutrition*	1.16 (0.93-1.45)	0.20	1.19 (0.92-1.55)	0.19	1.19 (0.90-1.59)	0.22	1.06 (0.76-1.48)	0.69
Severe malnutrition*	1.64 (1.30-2.06)	<0.0001	1.80 (1.39-2.34)	<0.0001	1.86 (1.40-2.46)	<0.0001	1.33 (0.94-1.89)	<0.10

Abbreviations used: Ref Reference Category
Footnotes: ¶ No signs of pneumonia taken as a reference
*Normal-weight of age z score> -2SD; Malnutrition-weight of age z ≤ -2SD and Severe malnutrition-weight of age z ≤ -3SD

For peer review only - http://bmjopen.bmj.com/site/about/guidelines.xhtml

DISCUSSION

This prospective hospital-based surveillance study was conducted to assess the radiological abnormalities in children (2-59 months) residing in pre-specified districts of Uttar Pradesh and Bihar, India and hospitalized with CAP. The study was conducted from January 2015 to April 2017, prior to introduction of PCV in NIP of Government of India [9]. In our study, among interpretable CXRs, we found 22.44% (635/2829) children had PEP alone or with infiltrates, other infiltrates only 12.09% (342/2829) and 65.46% (1852/2829) had normal CXRs findings. Our study used WHO case definition for CAP [18]. A panel of three trained radiologists interpreted CXRs, adopting WHO recommended methodology [11 12]. These make our study methodology robust and results generalizable.

In our study, there were 88.54% (2829/3195) interpretable CXRs. This is similar to 83% (3587/3973) interpretable CXRs reported by Pneumonia Etiology Research for Child Health (PERCH) study conducted on 4232 children (1-59 months) to assess the etiology of CAP in nine sites of seven countries [24] . Consistent with PERCH findings, a vaccine probe trial conducted in Gambia found the proportion of interpretable CXRs among unvaccinated cases of pneumonia to be 84.32% (242/287) [25].

There have been several studies in the past two decades, which have reported CXRs findings in hospitalized cases of CAP. Almost all of these were conducted before the introduction of PCV in their respective regions. A small prospective study conducted in Ethiopia reported radiological abnormality in CXRs to be 48.3% (95% CI 39.49-57.22) in 122 children (3 months-14 years) clinically diagnosed with WHO-defined severe pneumonia [26]. Similar findings were reported

For peer review only - http://bmjopen.bmj.com/site/about/guidelines.xhtml

from the Gambian vaccine probe trial where the proportion of radiological abnormality was 45% (95% CI: 43.35-46.46) among unvaccinated hospitalized cases of clinical pneumonia. PERCH study found that 54% (95% CI: 52.31-55.57) of CXRs among cases of CAP were abnormal [24]. In all of these studies, proportion of cases with abnormal CXRs is higher than 34.5% (95% CI 32.8-36.3) found by us in the current study. However, our findings are similar to PERCH rural study site of Matlab, Bangladesh that reported radiological abnormality in 35.3% (95% CI: 29.77-40.85) CXRs of hospitalized cases of CAP [24]. Another PERCH urban site of Dhaka, Bangladesh reported 63.10% (95% CI 56.18 -70.02) cases with abnormal CXRs [24]. In our study, radiological abnormalities in CXRs were higher in cases from largely urban districts of Patna and Lucknow compared to rural districts of Darbhanga and Etawah. This is consistent with rural-urban differences in Bangladesh sites of PERCH. Variation in CXR findings among cases of CAP may be due to infecting organism, immune response of patient and prior duration of disease.

In 2016, WHO's Department of Immunization, Vaccines and Biologicals standardized the categorization of radiological pneumonia and established that PEP can be taken as a good surrogate marker of SP in epidemiological and vaccine efficacy studies [12]. In our study, 22.44 % (95% C.I. 20.90 -23.98), CXRs were having PEP alone or with other infiltrates. This is similar to PERCH study that reported PEP alone or with other infiltrates in 27% (95% C.I. 25.50 -28.40) hospitalized cases of CAP [24]. Another study conducted in Gambia reported that 45% (95% CI: 43.35-46.46) non-vaccinated children had PEP and/or other infiltrates [25]. PEP has been associated with increased risk of treatment failure (p=0.002), increased length of hospitalization

For peer review only - http://bmjopen.bmj.com/site/about/guidelines.xhtml

(p=0.0003) and more days of respiratory support (p=0.002) in Botswana when compared with cases reporting `no significant pathology` on CXRs [27] .

In our study, female gender (p<0.001) was at the higher risk of developing radiological abnormalities compared to males (**table 3**). The results are in concordance with a hospital-based case-control study carried out in Brazil that reported male gender as a protective factor against pneumonia (OR = 0.53; 95 % CI 0.39–0.72) [28]. Another study in Mozambique, Africa reported that male gender was not significantly associated with presence of radiological abnormalities (OR =0.77 (95 % CI 0.56–1.05) in children (0-59 months) suffering from severe pneumonia [29]. However, in contrast, a Gambian study reported male preponderance for all pneumonia that was most marked for `other infiltrates/abnormalities` pneumonia [25].

A systematic review with meta-analysis conducted in 2019 suggests that no one clinical feature is sufficient on its own to diagnose of radiological pneumonia [30]. However other socio-demographic and clinical correlates of abnormal CXRs found by us (Model 1), which increased the risk of radiological abnormalities, were presence of pallor, severe malnutrition, longer duration of illness and exposure to biomass fuel. Exposure to biomass fuel at the time of cooking is an important factor that impacts the severity of CAP in developing countries [31]. In rural India, majority of the households use biomass fuel like firewood, dung cakes and wood for cooking [32]. Young children are at risk to adverse effects of exposure to biomass fuel as either the households have no separate cooking space or have poor ventilation and sometimes young children stay with their mother while she cooks.

For peer review only - http://bmjopen.bmj.com/site/about/guidelines.xhtml

Specific correlates of PEP/Radiological Pneumonia (Models II and III) possibly due to SP, other than those mentioned above, were presence of vomiting and ronchi on auscultation, both of which were found to be protective. These symptoms/signs are more often reported in viral pneumonia [33]. No specific correlates of radiological abnormalities of `other infiltrates` (Model IV) were found by us. Hence it is difficult to attribute radiological findings of other infiltrates to either bacterial or viral etiology.

Based on our study, almost two-third hospitalized cases of CAP had normal CXRs and this could be perhaps of viral etiology. This is supported by a recent study that reported 61.4% (95% CI 57·3–65·6) cases to be viral [33]. Among one-third of cases of CAP had abnormal CXRs and thus were more likely to be bacterial in etiology, and two-thirds of which were possibly due to SP.

CONCLUSION

Among hospitalized cases of community-acquired pneumonia, almost one-third children had abnormal chest radiographs of which about two-thirds had abnormalities related with possible bacterial etiology (SP). Hence, the introduction of pneumococcal vaccination is likely to reduce the burden of childhood pneumonia in India. Since the study was done prior to the introduction of PCV in India, continued surveillance will be required to assess the impact of PCV on radiological findings in cases admitted with CAP. The impact of introduction of PCV in NIP on under-five mortality rate and burden of CAP needs to be assessed.

Acknowledgements: We thank the state governments of Uttar Pradesh and Bihar for giving us permission to conduct the study. We extend our gratitude to participating tertiary and

For peer review only - http://bmjopen.bmj.com/site/about/guidelines.xhtml

secondary care hospitals for their contribution. Members of the Lucknow Community-Acquired Pneumonia (CAP) Group: King George's Medical University (KGMU), Lucknow, India: Prof. Shally Awasthi, Department of Pediatrics, Prof. Neera Kohli, Department of Radio-diagnosis, Prof. Monica Agarwal, Department of Community Medicine; Mr. Puneet Dhasmana, Project Staff; Patna Medical College and Hospital, Patna, Bihar-Prof. Neelam Verma and Prof. Chandra Bhushan Kumar, Department of Pediatrics; Darbhanga Medical College and Hospital, Darbhanga, Bihar, India- Prof. Chittaranjan Roy, Department of Community Medicine, Prof. Kripanath Mishra, Department of Pediatrics; Uttar Pradesh University of Medical Sciences, Etawah, Uttar Pradesh, India- Prof. Pankaj Kumar Jain, Department of Community Medicine, Prof. Rajesh Yadav, Department of Pediatrics.

Contributors: The study was conceived and designed by SA. CAP study group performed data acquisition. CMP and NM[3] conducted the statistical analysis of the data. The paper was written by SA, TV, MA and CMP. AC, NM[5], RCS and NK interpreted chest x-rays. All authors were involved with drafting and revising the work and approved the final submission.

Funding statement: The study was supported by Bill & Melinda Gates Foundation (https://www.gatesfoundation.org/) via Grant No: OPP1118005**.** Funding agency had no role in the design of the study and collection, analysis and interpretation of data, or in writing the manuscript.

Competing interests: None declared.

Patient consent for publication: Not required.

Ethics approval: The Institutional Ethics Committee of King George's Medical University (Lucknow), The Uttar Pradesh University of Medical Sciences (Etawah), Patna Medical College

For peer review only - http://bmjopen.bmj.com/site/about/guidelines.xhtml

and Hospital (Patna) and Darbhanga Medical College and Hospital (Darbhanga) gave ethical approval for the conduct of study.

Provenance and peer review: Not commissioned; externally peer reviewed.

Data sharing statement: The data contained within this study can be obtained by writing to shally07@gmail.com

Copyright information: This is an Open Access article distributed in accordance with the Creative Commons Attribution Non Commercial (CC BY-NC 4.0) license, which permits others to distribute, remix, adapt, build upon this work non-commercially, and license their derivative works on different terms, provided the original work is properly cited and the use is non-commercial. See: http://creativecommons.org/licenses/by-nc/4.0/

Supporting Information

S1 Appendix: Chest radiograph interpretation form

REFERENCES:

1. UNICEF U. Levels and trends in child mortality. Estimates developed by the UN inter-agency group for child mortality estimation. 2011.
2. Amouzou A, Velez LC, Tarekegn H, *et al*. One is too many: ending child deaths from pneumonia and diarrhoea.
3. Mathew JL, Patwari AK, Gupta P, *et al*. Acute respiratory infection and pneumonia in India: a systematic review of literature for advocacy and action: UNICEF-PHFI series on newborn and child health, India. *Indian Pediatr* 2011; 48: 191–218.
4. Farooqui H, Jit M, Heymann DL, *et al*. Burden of severe pneumonia, pneumococcal pneumonia and pneumonia deaths in Indian states: modelling based estimates. *PLoS One* 2015

For peer review only - http://bmjopen.bmj.com/site/about/guidelines.xhtml

5. Rudan I, O'brien KL, Nair H, *et al*. Epidemiology and etiology of childhood pneumonia in 2010: estimates of incidence, severe morbidity, mortality, underlying risk factors and causative pathogens for 192 countries. *Journal of global health*. 2013 Jun;3(1).
6. Leung DT, Chisti MJ, Pavia AT. Prevention and control of childhood pneumonia and diarrhea. *Pediatric Clinics*. 2016 Feb 1;63 (1):67-79.
7. Virkki R, Juven T, Rikalainen H, *et al*. Differentiation of bacterial and viral pneumonia in children. *Thorax*. 2002 May 1;57(5):438-41.
8. World Health Organization. Pneumococcal conjugate vaccines in infants and children under 5 years of age: WHO position paper–February 2019. Accessed 12th July 2019.
9. Government of India. Introduction of Pneumococcal Conjugate Vaccine: Operational Guidelines. Ministry of Health and Family Welfare, 2017 Available at: https://nhm.gov.in/New_Updates_2018/NHM_Components/Immunization/Guildelines_for_immunization/Operational_Guidelines_for_PCV_introduction.pdf Assessed 12th July 2019.
10. Cherian T, Mulholland EK, Carlin JB, *et al*. Standardized interpretation of paediatric chest radiographs for the diagnosis of pneumonia in epidemiological studies. *Bulletin of the World Health Organization*. 2005;83:353-9
11. Pneumonia Vaccine Trial Investigators' Group. Standardization of Interpretation of Chest Radiographs for the Diagnosis of Pneumonia in Children. Geneva: Department of Vaccines and Biologicals, World Health Organization; 2001
12. Mahomed N, Fancourt N, De Campo J, *et al*. Preliminary report from the World Health Organization chest radiography in epidemiological studies project. *Pediatric radiology*. 2017 Oct 1;47 (11):1399-404.
13. Greenberg D, Givon-Lavi N, Ben-Shimol S, *et al*. Impact of PCV7/PCV13 introduction on community-acquired alveolar pneumonia in children< 5 years. *Vaccine*. 2015 Aug 26; 33(36):4623-9. 6.
14. Sutcliffe CG, Shet A, Varghese R, *et al*. Nasopharyngeal carriage of Streptococcus pneumoniae serotypes among children in India prior to the introduction of pneumococcal conjugate vaccines: a cross-sectional study. *BMC Infect Dis*. 2019 Dec;19(1):605.
15. Annual health survey (AHS) factsheet 2012–13 Uttar Pradesh [Internet]. New Delhi: Office of Registrar General & Census Commissioner, Ministry of Home Affairs, Government of India; 2010–2011. Available

For peer review only - http://bmjopen.bmj.com/site/about/guidelines.xhtml

from:http://www.censusindia.gov.in/vital_statistics/AHSBulletins/AHS_Factsheets_2012-13/FACTSHEET-UTTAR_PRADESH.pdf [cited 2019 Aug 8].

16. Annual health survey (AHS) factsheet 2012–13 Bihar [Internet]. New Delhi: Office of Registrar General & Census Commissioner, Ministry of Home Affairs, Government of India; 2010–2011. Available from:http://www.censusindia.gov.in/vital_statistics/AHSBulletins/AHS_Factsheets_2012-13/FACTSHEET-Bihar.pdf [cited 2019 Aug 8].
17. Awasthi S, Pandey CM, Verma T, *et al*. Incidence of community-acquired pneumonia in children aged 2-59 months of age in Uttar Pradesh and Bihar, India, in 2016: An indirect estimation. *PloS one*. 2019 Mar 20;14(3):e0214086.
18. World Health Organization. Revised WHO classification and treatment of childhood pneumonia at health facilities. Geneva: World Health Organization. 2014:6-14.
19. Awasthi S, Singh JV, Kohli N, *et al*. Hospital-based surveillance for radiological pneumonia in children under 5 years of age in Uttar Pradesh and Bihar. *Pediatric Infectious Disease*. 2016 Jun 30;8(2):52-7.
20. Bassignani MJ, Bubash-Faust L, Ciambotti J, *et al*. Conversion of teaching file cases from film to digital format: a comparison between use of a diagnostic-quality digitizer and use of a flatbed scanner with transparency adapter. *Acad Radiol*. 2003 May 1;10(5):536-42.
21. Dean AG, Arner AT, Sunki GG, *et al*. Epi Info™, a database and statistics program for public health professionals. Atlanta, GA, USA: CDC; 2011.
22. De Onis M, Blossner M, World Health Organization. WHO global database on child growth and malnutrition. Geneva: World Health Organization; 1997.
23. SPSS Inc. Released 2009. SPSS for Windows [software], Version 22·0. Chicago, SPSS Inc. Available from:www.spss.com
24. Fancourt N, Deloria Knoll M, Baggett HC, *et al*. Chest radiograph findings in childhood pneumonia cases from the multisite PERCH study. *Clin Infect Dis*. 2017 May 29;64(suppl_3):S262-70
25. Enwere G, Cheung YB, Zaman SM, *et al*. Epidemiology and clinical features of pneumonia according to radiographic findings in Gambian children. *Trop Med Int Health*. 2007 Nov;12(11):1377-85.

For peer review only - http://bmjopen.bmj.com/site/about/guidelines.xhtml

26. Meiron Hassen, Alemayehu Toma, Mulugeta Tesfay, *et al*. Radiologic Diagnosis and Hospitalization among Children with Severe Community Acquired Pneumonia: A Prospective Cohort Study. *Biomed Res Int*, vol. 2019, Article ID 6202405, 8 pages, 2019. https://doi.org/10.1155/2019/6202405.
27. Kelly MS, Crotty EJ, Rattan MS, *et al*. Chest radiographic findings and outcomes of pneumonia among children in Botswana. *Pediatr Infect Dis J*. 2016 Mar;35(3):257.
28. da Fonseca Lima EJ, Mello MJ, Lopes MI, *et al*. Risk factors for community-acquired pneumonia in children under five years of age in the post-pneumococcal conjugate vaccine era in Brazil: a case control study. *BMC Pediatr*. 2016 Dec;16(1):157.
29. Sigauque B, Roca A, Bassat Q, *et al*. Severe pneumonia in Mozambican young children: clinical and radiological characteristics and risk factors. *J Trop Pediatr*. 2009 Apr 28;55(6):379-87.
30. Rambaud-Althaus C, Althaus F, Genton B, *et al*. Clinical features for diagnosis of pneumonia in children younger than 5 years: a systematic review and meta-analysis. *Lancet Infect Dis*. 2015 Apr 1;15(4):439-50
31. Awasthi S, Glick AH, Fletcher RH. Effect of cooking fuels on Respiratory Diseases in Pre-school children in Lucknow, India. *Am J Trop Med Hyg* 1996; 55: 48-51
32. Tielsch JM, Katz J, Thulasiraj RD, *et al*. Exposure to indoor biomass fuel and tobacco smoke and risk of adverse reproductive outcomes, mortality, respiratory morbidity and growth among newborn infants in south India. *Int J Epidemiol*. 2009 Sep 16;38 (5):1351-63.
33. O'Brien KL, Baggett HC, Brooks WA, *et al*. Causes of severe pneumonia requiring hospital admission in children without HIV infection from Africa and Asia: the PERCH multi-country case-control study. *The Lancet*. 2019 Jun 27

Figure Legend

Figure 1: Flow diagram of cases of community acquired pneumonia recruited from participating districts before the introduction of pneumococcal conjugate vaccine (January 2015-April 2017)

For peer review only - http://bmjopen.bmj.com/site/about/guidelines.xhtml

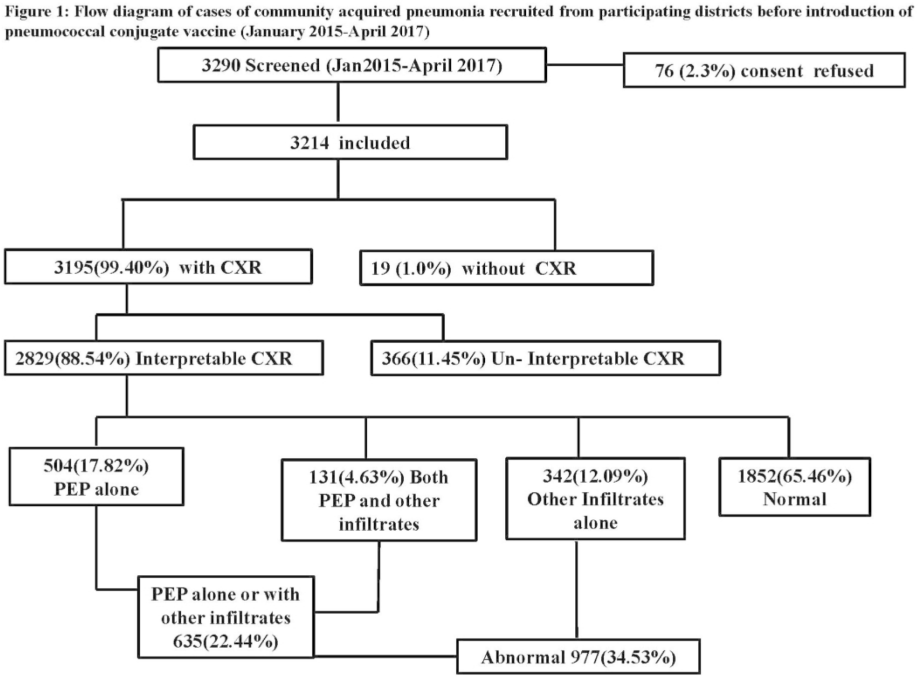

Figure 1: Flow diagram of cases of community acquired pneumonia recruited from participating districts before the introduction of pneumococcal conjugate vaccine (January 2015-April 2017)

254x190mm (300 x 300 DPI)

Childhood Pneumonia Surveillance

Department of Paediatrics, KGMU, Lucknow ,UP

Form-R

(RADIOLOGY REPORT FORM)

1	**Drs_ID**	[__][__][__][__]
	**Radiology Report**	**Patient Details**
2	**IDNo:**	[__] / [__] / [__][__][__]/[__][__][__][__]<br>State /District / Unit / Subject number<br>*(For office use)*
2	Date Of Report	[__][__]/[__][__]/[__][__][__][__] (DD/MM/YYYY)
	**Report Details**	**Findings (tick one)**
3	Image Quality	Adequate ☐ Suboptimal ☐ Un-interpretable ☐
4	Significant Pathology	Yes ☐ No ☐ Un-interpretable ☐
5	**End Point Consolidation**	
5a	Left	Yes ☐ No ☐ Un-interpretable ☐
5b	Right	Yes ☐ No ☐ Un-interpretable ☐<br>Uninterpretable
6	Other Infiltrates/Abnormalities	
6a	Left	Yes ☐ No ☐ Un-interpretable ☐
6b	Right	Yes ☐ No ☐ Un-interpretable ☐
7	Pleural Fluid	
7a	Left	Yes ☐ No ☐ Un-interpretable ☐
7b	Right	Yes ☐ No ☐ Un-interpretable ☐
8	Comments:	[ ]
9	Conclusion:	a) Primary endpoint pneumonia only ☐<br>b) Other infiltrate only ☐<br>c) Both PEP and other infiltrate ☐<br>d) Normal ☐<br>e) Un-interpretable for any findings☐

Chest Radiograph Findings in children aged 2-59 months hospitalized with Community-Acquired Pneumonia, prior to the introduction of Pneumococcal Conjugate Vaccine in India- A Prospective Multisite Observational Study

Journal:	*BMJ Open*
Manuscript ID	bmjopen-2019-034066.R1
Article Type:	Original research
Date Submitted by the Author:	02-Jan-2020

Complete List of Authors:	Awasthi, Shally; King George's Medical University, Pediatrics<br>Rastogi, Tuhina; King George's Medical University, Paediatrics<br>Mishra, Neha; King George's Medical University, Pediatrics<br>Chauhan, Abhishek ; Dr Ram Manohar Lohia Institute of Medical Sciences, Department of Radio-diagnosis<br>Mohindra, Namita; Sanjay Gandhi Post Graduate Institute of Medical Sciences, Department of Radio-diagnosis<br>Shukla, Ram ; Banaras Hindu University, Varanasi, India, Institute of Medical Sciences<br>Agarwal, Monica; King George's Medical University, Department of Community Medicine<br>Pandey, CM; Sanjay Gandhi Post Graduate Institute of Medical Sciences, Biostatistics & Health Informatics<br>Kohli, Neera ; King George's Medical University, Department of Radio-diagnosis<br>Study Group, CAP; CAP study Group

**Primary Subject Heading**:	Paediatrics
Secondary Subject Heading:	Global health
Keywords:	Chest radiographs, Hospitalized community-acquired pneumonia, under-five, Streptococcus pneumoniae, India

SCHOLARONE™
Manuscripts

I, the Submitting Author has the right to grant and does grant on behalf of all authors of the Work (as defined in the below author licence), an exclusive licence and/or a non-exclusive licence for contributions from authors who are: i) UK Crown employees; ii) where BMJ has agreed a CC-BY licence shall apply, and/or iii) in accordance with the terms applicable for US Federal Government officers or employees acting as part of their official duties; on a worldwide, perpetual, irrevocable, royalty-free basis to BMJ Publishing Group Ltd ("BMJ") its licensees and where the relevant Journal is co-owned by BMJ to the co-owners of the Journal, to publish the Work in this journal and any other BMJ products and to exploit all rights, as set out in our licence.

The Submitting Author accepts and understands that any supply made under these terms is made by BMJ to the Submitting Author unless you are acting as an employee on behalf of your employer or a postgraduate student of an affiliated institution which is paying any applicable article publishing charge ("APC") for Open Access articles. Where the Submitting Author wishes to make the Work available on an Open Access basis (and intends to pay the relevant APC), the terms of reuse of such Open Access shall be governed by a Creative Commons licence – details of these licences and which Creative Commons licence will apply to this Work are set out in our licence referred to above.

Other than as permitted in any relevant BMJ Author's Self Archiving Policies, I confirm this Work has not been accepted for publication elsewhere, is not being considered for publication elsewhere and does not duplicate material already published. I confirm all authors consent to publication of this Work and authorise the granting of this licence.

For peer review only

Title: *Chest Radiograph Findings in children aged 2-59 months hospitalized with Community-Acquired Pneumonia, prior to the introduction of Pneumococcal Conjugate Vaccine in India- A Prospective Multisite Observational Study*

Short Title: *Radiological Findings in Children hospitalized with Community-Acquired Pneumonia in India Pre-Pneumococcal Conjugate Vaccine introduction*

Shally Awasthi[1]*, Tuhina Rastogi[1], Neha Mishra[1], Abhishek Chauhan[2], Namita Mohindra[3], Ram Chandra Shukla[4], Monica Agarwal[5], Chandra Mani Pandey[6], Neera Kohli[7] and CAP Group^

[1]Department of Pediatrics, King George's Medical University, Lucknow, India
[2]Department of Radio-diagnosis, Dr Ram Manohar Lohia Institute of Medical Sciences, Lucknow, India
[3]Department of Radio-diagnosis, Sanjay Gandhi Post Graduate Institute of Medical Sciences, Lucknow, India
[4]Department of Radio-diagnosis, Institute of Medical Sciences, Banaras Hindu University, Varanasi, India
[5] Department of Community Medicine, King George's Medical University, Lucknow, India
[6]Department of Biostatistics and Health Informatics, Sanjay Gandhi Postgraduate Institute of Medical Sciences, Lucknow, India
[7]Department of Radio-diagnosis, King George's Medical University, Lucknow, India

^Members of the Lucknow CAP Group are given in the Acknowledgement

*Corresponding Author
Postal Address: Prof. Shally Awasthi, Head, Department of Paediatrics, King George's Medical University, Lucknow , India Pincode: 226003
Mobile: +91-9839221244
E-mail: shally07@gmail.com

Word Count: 4533 Tables: 3 Figure:1

For peer review only - http://bmjopen.bmj.com/site/about/guidelines.xhtml

ABSTRACT

Objectives: To assess radiological abnormalities in chest X-rays and to identify the demographic and clinical correlates of specific radiological abnormalities in children aged 2-59 months, hospitalized with World Health Organization defined community-acquired pneumonia, residing in pre-specified districts of Uttar Pradesh and Bihar, India.

Design: Prospective, hospital-based surveillance

Setting: Multi-site study conducted in a network of 117 secondary/tertiary care hospitals in four districts of Uttar Pradesh and Bihar, India.

Participants: Included were children aged 2-59 months hospitalized with community-acquired pneumonia, residing in project district, with duration of illness of <14 days and who were not hospitalized elsewhere for this episode nor had been recruited previously.

Main outcome measure: Radiological abnormalities in the chest X-rays, where there was concordance between two or more of the panel of three trained radiologists.

Results: From January 2015 to April 2017, 3214 cases were recruited and in 99.40 % (3195/3214) chest X-rays were available. Among 88.54 % (2829/3195) interpretable X-rays, 34.53 % (977/ 2829, 95% C.I. 32.78 -36.28) had some radiological abnormalities, while the rest were normal. Primary endpoint pneumonia alone or with other infiltrates was found in 22.44 % (635/2829, C.I. 20.90 %-23.98 %), other infiltrates only in 12.09% (342/ 2829; C.I. 10.88 %-13.29 %). There was a statistically significant inter-district variation in radiological abnormality. Statistically significantly higher proportion of abnormal chest X-ray was found among girls, those with weight-for-age z score ≤-3 SD, longer duration of fever, pallor and with exposure to biomass fuel.

For peer review only

For peer review only - http://bmjopen.bmj.com/site/about/guidelines.xhtml

Conclusions: Among hospitalized cases of community-acquired pneumonia, almost one-third children had abnormal chest radiographs of which about two-thirds had abnormalities related with possible bacterial etiology (*Streptococcus pneumoniae*). Hence introduction of pneumococcal vaccination is likely to reduce burden of childhood pneumonia in India.

Key words: Chest radiographs, Hospitalized community-acquired pneumonia, under-five, *Streptococcus pneumoniae*, India

Strengths and Limitations of the Study

- Prospective, multisite recruitments from a large hospital surveillance network established for the project in four districts in two states of India that have high under-five mortality rates
- Standard World Health Organization definition was used for identifying hospitalized cases of clinical pneumonia
- Radiological abnormalities interpreted by a panel of three trained radiologists at locations out of the surveillance network, blinded to each other as well as clinical features of the case
- Since pre-existing X-rays machines were used in this pragmatic study, there was a variation in the quality of images obtained, which were minimized by digitizing them centrally
- Since the objective of the study was to assess the radiological abnormalities in chest X-rays of recruited cases, clinical data was recorded by pre-existing hospital staff, there could be some inter-observer variations.

For peer review only - http://bmjopen.bmj.com/site/about/guidelines.xhtml

INTRODUCTION

Community-acquired pneumonia (CAP) is the single largest infectious cause of death in young children worldwide. Globally, pneumonia accounts for 16% of deaths in children under-five years of age and results in almost one million deaths (0.9 million children in 2016) every year[1 2]. Most deaths due to pneumonia occur in low and middle income countries particularly in sub-Saharan Africa and South Asia[2 3]. In India, there were approximately 0·44 million under-five deaths due to CAP in the year 2015 [4].

CAP could have viral or bacterial etiology[5 - 7]. Etiology varies from country to country and also across different time periods. Pediatric bacterial pneumonia is predominantly caused by *Streptococcus pneumoniae* (*SP*) and *Hemophilus influenzae Type B* (HiB) while Respiratory syncytial virus and Influenza A or B virus are important contributors of pediatric viral pneumonia[5 6]. The World Health Organization (WHO) recommends the introduction of Pneumococcal Conjugate Vaccine (PCV) in the national immunization programme of countries with high child mortality rates, which includes India [8]. Consequently, PCV-13 was launched in May 2017 under the national immunization programme of five Indian states (Uttar Pradesh, Bihar, Rajasthan, Madhya Pradesh and Himachal Pradesh) in a phased manner[9]. It is expected to be rolled out in other parts of the country in the near future. Vaccination against HiB is already under the national immunization programme since 2011.

Differentiating bacterial from viral etiology of CAP on clinical features or by investigations remains difficult [7 10 11]. Several PCV probe trials have used radiographically confirmed alveolar

For peer review only - http://bmjopen.bmj.com/site/about/guidelines.xhtml

pneumonia, also called end-point pneumonia, to be an outcome for vaccine efficacy and this has been endorsed by WHO [12-14].

The current study was a hospital-based surveillance to assess the radiological abnormalities in chest X-rays (CXRs) and to identify the demographic and clinical correlates of specific radiological abnormalities in children aged 2-59 months, hospitalized with WHO defined CAP, residing in pre-specified districts of Uttar Pradesh and Bihar, India.

METHODS

Study design and Setting

This prospective multi-site observational study was conducted in Lucknow and Etawah districts of Uttar Pradesh and Patna & Darbhanga districts of Bihar, India. Uttar Pradesh is the first most populated and Bihar third most populated state of India[15 16]. In Lucknow district 66.2% population resides in urban areas and in Patna district 43.07% [15 16]. In contrast, only 22.3% population of Etawah district and 9.74% population of Darbhanga district resides in urban areas [15 16]. All four project districts have alarmingly high infant and child mortality indicators [15-17]. The under-five mortality rates of Lucknow (58/1000), Etawah (85/1000), Patna (46/1000) and Darbhanga (77/1000) districts are above the national average (50/1000) [15-17]. Similarly, the infant mortality rates of Lucknow (44/1000), Etawah (56/1000), Patna (31/1000) and Darbhanga (44/1000) districts are also higher than the national average (41/1000) [15-17].

Study Population

For peer review only

For peer review only - http://bmjopen.bmj.com/site/about/guidelines.xhtml

A hospital-based surveillance system was established for this study[17 18]. Included in the surveillance were public and private hospitals of study districts which provided either secondary or tertiary level care to admitted children. All children (2-59 months), hospitalized in network hospitals between January 2015 to April 2017, with history of fast breathing with/without chest in-drawing were screened [18].

Included were children hospitalized with symptoms of WHO defined CAP and residing in the project district [18]. WHO has developed guidelines for hospital-based management of common childhood illness such as pneumonia [19]. According to these guidelines, fast breathing ≥ 50 breaths/minute in a child aged 2–11 months and ≥ 40 breaths/minute in a child aged 12-59 months along with chest indrawing was categorized as having `pneumonia`[19]. A child presenting with cough or difficulty in breathing with: (a) oxygen saturation < 90% or central cyanosis (b) severe respiratory distress (e.g. grunting, very severe chest indrawing) and (c) signs of pneumonia with a general danger sign (inability to breastfeed or drink, lethargy or reduced level of consciousness, convulsions) was categorized as having `severe pneumonia`[19]. Excluded were children with cough for ≥ 14 days or those that had been hospitalized in last 14 days [18].

Sample Size

We assumed that the incidence of radiological pneumonia is 3/100 child years of observations. Then for a margin of error of 1.5/100 child years of observation, incidence of pneumonia in the community of 20/100 child years of observation, alpha level of 0.05, and power of 90% when the estimated population of children under-five years of age in Lucknow district [20] is 750,000; 693 cases had to be included per district.

For peer review only - http://bmjopen.bmj.com/site/about/guidelines.xhtml

Data collection

Data was collected by surveillance officers who had postgraduate degree in social sciences and almost 10 years experience in community based health research. After recruitment, they were imparted six-day centralized training on project procedures and logistics. Class-room as well as practical skills-training was given by the coordinating centre in Lucknow. Pre and post tests were conducted to ascertain knowledge and skills acquired by them through the training to ensure quality in data collection. The coordinating centre provided annual refresher trainings to the surveillance officers from all four sites in Lucknow.

After obtaining written, informed consent of the caregivers, data was collected through face-to-face interviews with them as well as by abstraction from hospital records. Socio-demographic data, obtained by interviewing caregivers, was: child`s age, gender, residence, birth order, immunization status, current breastfeeding status, parental education and occupation, smoking status of parents, family type, housing infrastructure, use of biomass fuel etc. Caregivers were also asked about the symptoms of disease and its duration in days.

Clinical data, recorded by pre-existing hospital staff at the time of hospitalization, was abstracted. Where available, data was collected on anthropometry (weight and height), fever (axillary temperature ≥37.5°C), oxygen saturation by pulse oxymetry, pallor, central cyanosis, and danger signs of pneumonia and vital signs (heart rate and respiratory rate). Presence of wheezing on auscultation of chest was abstracted, when recorded. At the hospitals, clinicians generally used Integrated Management of Childhood Illness (IMCI) definitions[21] to identify pallor, cyanosis, wheeze on auscultation and general danger sign as it is incorporated in their

For peer review only - http://bmjopen.bmj.com/site/about/guidelines.xhtml

medical undergraduate training. Most doctors of public health sector also receive a formal in-service training on IMCI [21]. Clinical outcome (survival or mortality) was noted from hospital records on follow up.[17] [18].

Chest x-ray (CXR) image acquisition and archiving

CXR (poster-anterior view) was done when advised by the treating physician. These CXRs were obtained by the surveillance staff at the time of recruitment. CXRs were either analog or digital. In case of digital CXRs, second copy was obtained where possible. If only single analog image was available, then the hardcopy of CXR was obtained from the caregiver after the child was discharged. If this could not be done, image of the hardcopy was captured. CXR machines were not provided through the project.

CXRs of recruited cases were subsequently scanned and converted into digital format using a diagnostic-quality film image digitalizer (Microteck International Limited, model Medi 6000 plus) [22]. CXRs obtained/converted into digital image were stored as per the standard operating procedure and were subsequently archived for web-based radiological interpretation. Digital images were stored in JPEG format at 300 dpi resolution. Each CXR file was anonymized and given a unique identification number. Digital CXRs were uploaded on online data management software, developed especially for the project.

Interpretation of radiological images

A panel of radiologists was constituted for standardized interpretations of CXRs. Four radiologists were part of this panel, one of whom was Project co-investigator-Radiology (NK).

For peer review only - http://bmjopen.bmj.com/site/about/guidelines.xhtml

All radiologists are faculty in medical teaching institutes and also look after pediatric radiology. They all have more than fifteen years experience in interpreting pediatric CXRs.

Radiologists were trained according to the methodology developed by Department of Immunization, Vaccines, and Biologicals of the WHO [11]. An international WHO-certified trainer from the International Centre for Diarrhoeal Disease Research, Bangladesh imparted a two-day in-house training to the radiologists. Training objective was to standardize interpretation and coding of CXRs, to develop a CXR reporting form **[S1 Appendix]** and to provide training on web-based CXR retrieval and reporting system. During the training, 210 CXRs of WHO data set were used. For assessing concordance post training, another set of 48 CXRs was provided for interpretation to individual radiologists. Post-test agreement with WHO findings was calculated, which was about 80%. Inter-observer variation was about 25% and was for minor interpretation like quality of film, end point infiltrates etc. Repeat training was conducted on an additional set of 44 CXRs provided by WHO to ensure standardization in interpretation. Thereafter, concordance achieved by the radiologists was reviewed quarterly by the study arbitrator. Radiologists met annually to review key concepts and discuss challenges faced in interpreting CXRs.

After training, radiologists independently reviewed CXRs and registered their findings in an online standardized chest radiograph interpretation form **[S1 Appendix].** For optimal viewing of CXRs, all radiologists used similar workstations. Specifications were provided for the computer monitor and hardware to be used. It was ensured that monitors had the correct brightness and contrast adjustment for optimal viewing.

For peer review only - http://bmjopen.bmj.com/site/about/guidelines.xhtml

During online evaluation, radiologists recorded the quality of film, findings of CXRs and conclusion. Radiologists interpreted film quality as follows: (a) `*Adequate/optimal*` for features that allow confident interpretation of consolidation and pleural effusion as well as other infiltrates; (b) `*Suboptimal*` for features that allow interpretation of consolidation and pleural effusion, but not of other infiltrates or findings and (c) `*Un-interpretable*` pertaining to features of the image that are not interpretable with respect to presence or absence of consolidation or pleural effusion without additional images[12].

After interpreting film quality, radiologists interpreted the pathological findings. For each radiographic finding, there were two options to be chosen: `yes` for the presence of pathological findings and `no` for its absence. Pathological findings were classified into significant pathology (including pleural effusion) and the presence of consolidation and infiltrates. `*End-point consolidation*` for CXRs was defined as dense or confluent opacity that occupies a portion or whole of a lobe or the entire lung, that may or may not contain air bronchograms. Portion of the lung would mean the opacity covering the width of intercostal spaces plus the width of one adjacent rib. `*Other (non end-point) infiltrate*` for CXRs was defined as linear and patchy opacities (interstitial infiltrate) in a lacy pattern, featuring peri-bronchial thickening and multiple areas of atelectasis or minor patchy infiltrates that are not of sufficient magnitude to constitute endpoint consolidation, and small areas of atelectasis that in children may be difficult to distinguish from consolidation. `*Pleural effusion*` was defined as presence of fluid in the lateral pleural space between the lung and chest wall that is spatially associated with a pulmonary parenchymal infiltrate (including `other infiltrate`) or has obliterated enough of the hemithorax to

For peer review only - http://bmjopen.bmj.com/site/about/guidelines.xhtml

obscure any infiltrate. In most cases, this will be seen at the costo-phrenic angle or as a layer of fluid adjacent to the lateral chest-wall. This does not include fluid seen in the horizontal or oblique fissures [12].

Final conclusions were categorised into: (a) `*Primary End Point Pneumonia only*` (PEP) on the presence of consolidation or pleural effusion; (b) `*Other (non end-point) infiltrate only*` on the presence of other (non-consolidation) infiltrates as defined above in the absence of a pleural effusion (c) `*Both PEP and other infiltrate*` and (d) `*Normal*` when there were no findings consistent with 'endpoint consolidation' or 'other infiltrate' or `pleural effusion`[12].

After radiological interpretation, online data was archived, stored and checked for inconsistencies and completeness by the data manager. CXRs with concordant and discordant interpretations were identified. Interpretations were considered concordant when there was an agreement between two or more radiologists and discordant if all the three radiologists disagreed. Discordant interpretations were forwarded to the study arbitrator (NK). Arbitrator assessed discordant CXRs online and her interpretation was taken as final.

Data management and statistical analysis

Clinical data of hospital surveillance network was entered online in customized software. Primary entry was done by the four participating sites. Secondary data entry was done by the coordinating site in separate customized software. Anonymized CXRs were uploaded on customized software. Each of the three panelists assessed the CXRs online, blind to peer

For peer review only - http://bmjopen.bmj.com/site/about/guidelines.xhtml

182 assessments as well as clinical features of the case. CXR assessment data was downloaded from
183 the online software in MS Access database.

184

185 Exploratory data analysis was performed for detection of outlier and missing observations for all
186 the variables. Descriptive statistics was calculated for continuous variables as mean ± standard
187 deviation and categorical variables in percent. Un-interpretable CXRs were not analyzed. Among
188 interpretable CXRs, those radiological abnormalities where there was concordance between the
189 two radiologists, were taken as final. Weight-for-age (WAZ) z-score each child was calculated
190 using WHO Anthro Survey Analyser [23]. Weight of 7.59% (215/2829) children was missing in
191 our data. Missing weight of recruited children was estimated using regression based imputation
192 technique [24]. Kappa statistics was performed for agreement analysis among radiologists for
193 CXRs findings. Statistical analysis was performed using SPSS version 22.0 (Chicago, IL) [25]. A
194 p-value of <0·05 was taken as statistically significant using a two-tailed distribution.

195

196 Univariate analysis was performed to evaluate heterogeneity, stratified by four participating sites
197 for socio-demographic variables such as child`s age, gender, residence, birth order,
198 immunization status, current breastfeeding status, parental education and occupation, smoking
199 status of parents, family type, housing infrastructure, use of biomass fuel and for clinical
200 variables such as weight, height, duration of fever and oxygen saturation.

201

202 We report proportions of radiological abnormalities among hospitalized children for CAP by
203 four districts. Univariate analysis was performed to assess association of socio-demographic
204 variables and clinical signs of CAP with radiological abnormalities. Chi-square test was used for

For peer review only - http://bmjopen.bmj.com/site/about/guidelines.xhtml

categorical variables and student's t-test for continuous variables. ANOVA test was used for more than two groups to test the significance of continuous variables. Multivariate unconditional logistic regression was performed find association of presence of various radiological abnormalities, controlling for district of residence and other variables that had univariate association with radiological abnormalities (p value ≤ 0.2) and/or were clinically meaningful.

We developed four models and in these four models dependent (outcome) were:

Model I: Abnormal vs. Normal

Model II: Primary End Point Pneumonia (PEP) alone or with infiltrates vs. Normal

Model III: Primary End Point Pneumonia (PEP) alone vs. Normal

Model IV**:** Other infiltrates only vs. Normal

Independent variables that were kept across all the four models were : participating districts, age, gender, use of biomass fuel, symptoms of CAP such as duration of illness, presence of wheeze on ascultation, pallor, vomiting everything and malnutrition status of the case [WAZ$\leq$ -2 SD (malnourished) and WAZ $\leq$ -3 SD (severely malnourished)].

Patient and public involvement in research

Patients or public were not involved in the development of research question, study design or conducting the research. Reporting of this research conforms to the guidelines for Strengthening the Reporting of Observational Studies in Epidemiology (STROBE) [26]**.**

RESULTS

For peer review only - http://bmjopen.bmj.com/site/about/guidelines.xhtml

A total of 3290 hospitalized cases were screened in hospital surveillance network of Lucknow and Etawah districts of Uttar Pradesh and Patna & Darbhanga districts of Bihar. Out of these, 3214 cases fulfilling the WHO diagnosis of CAP were included **[Figure 1].** Among them, 3195 (99.40%) cases were enrolled with CXRs and only in 19 (1.0%) cases CXRs were not done. Out of these 88.54% (2829/3195) CXRs were found interpretable and remaining 11.45% (366/3195) were found un-interpretable by radiologists. Among interpretable CXRs, we found 22.44% (635/2829) children had primary end point pneumonia (PEP) alone or with infiltrates, 12.09% (342/2829) other infiltrates only and 65.46% (1852/2829) had normal CXRs findings [**Figure 1**]. Concordance among ≥ 2 radiologists for CXRs findings was 86.0%. Kappa statistics was calculated for agreement of CXRs findings between Reader 1 versus Reader 2 (K_1=0.31), Reader 2 versus Reader 3 (K_2=0.46) and Reader 3 versus Reader 1(K_3=0.42).

Table 1 shows univariate distribution of socio-demographic and clinical variables among hospitalized cases across four participating districts. A statistically significant variation was observed in all socio-demographic variables such as place of residence, type of house, type of family, maternal and paternal education and occupation, use of biomass fuel and parental smoking status across the four districts. We also report clinical variables of recruited cases across the four districts in **table 1**. Oxygen saturation by pulse-oxymetry was statistically significantly different across the sites, the proportion of cases with oxygen saturation ≤90 % was found also found statistically significant in children across four districts ($p < 0.0001$).

Table 1: Distribution of socio-demographic and clinical variables among hospitalized children for participating districts (Jan 2015-April 2017)

Characteristics	Lucknow	Etawah	Patna	Darbhanga	Total	
**Socio-demographic**	**n=1025**	**n=389**	**n=744**	**n=671**	**N=2829**	**p value**

For peer review only - http://bmjopen.bmj.com/site/about/guidelines.xhtml

Characteristics	(%)	(%)	(%)	(%)	(%)	
**Gender**						
Male	659(64.29)	287(73.78)	557(74.87)	502(74.81)	2005(70.87)	<0.0001
**Place of residence**						
Rural	195(19.02)	279(71.72)	304(40.86)	614(91.51)	1392(49.20)	<0.0001
Urban	830(80.98)	110(28.28)	440(59.14)	57(8.49)	1437(50.80)	
**Family Type**						
Joint	688(67.12)	360(92.54)	707(95.03)	383(57.08)	2138(75.57)	<0.0001
Nuclear	337(32.88)	29(7.46)	37(4.97)	287(42.77)	690(24.39)	
**House type**						
Mud	64(6.24)	54(13.88)	123(16.53)	374(55.74)	615(21.74)	
Bricks	854(83.32)	256(65.81)	453(60.89)	85(12.67)	1648(58.25)	<0.0001
Combined	107(10.4)	79(20.31)	168(22.58)	212(31.59)	566(20.01)	
**Mother's Education**						
No formal education	203(19.80)	56(14.40)	328(44.09)	496(73.92)	1083(38.28)	
Class I-V	108(10.54)	28(7.20)	82(11.02)	38(5.66)	256(9.05)	<0.0001
Class VI-XII	379(36.98)	176(45.24)	243(33.66)	112(16.69)	910(32.17)	
Graduate/ Post graduation	335(32.68)	129(33.16)	91(12.23)	25(3.73)	580(20.50)	
**Father's Education**						
No formal education	167(16.29)	29(7.46)	153(20.56)	345(51.42)	694(24.53)	
Class I-V	85(8.29)	19(4.88)	91(12.23)	82(12.22)	277(9.79)	
Class VI-XII	437(42.63)	206(52.96)	328(44.09)	205(30.55)	1176(41.57)	<0.0001
Graduate/ Post graduation	336(32.78)	135(34.70)	172(23.12)	39(5.81)	682(24.11)	
**Birth Order**						
1st	435(42.44)	187(48.07)	315(42.34)	192(28.61)	1129(39.91)	
2nd	343(33.46)	120(30.85)	235(31.59)	258(38.45)	956(33.79)	<0.0001
3rd	153(14.93)	47(12.08)	129(17.34)	137(20.42)	466(16.47)	
More than 3rd	93(9.07)	35(9.00)	62(8.33)	83(12.37)	273(9.65)	
**Immunization Status**						
Complete for age	792(77.27)	300(77.12)	711(95.56)	544(81.07)	2347(82.96)	
Incomplete for age	220(21.46)	84(21.59)	25(3.36)	126(18.78)	455(16.08)	<0.0001
Unimmunized	13(1.27)	5(1.29)	8(1.08)	1(0.15)	27(0.95)	
**Currently Breast Feeding**						
Yes	653(63.71)	256(65.81)	589(79.17)	537(80.03)	2035(71.93)	<0.0001
No	372(36.29)	133(34.19)	155(20.83)	134(19.97)	794(28.07)	
**Father's Occupation**						
Unemployed	13(1.27)	20(5.14)	27(3.63)	63(9.39)	123(4.35)	
Daily wages	329(32.10)	81(20.82)	165(22.18)	474(70.64)	1049(37.08)	
Salaried/ Professional	397(38.73)	104(26.74)	245(32.93)	55(8.20)	801(28.31)	<0.0001
Self-Employment	286(27.90)	184(47.30)	307(41.26)	79(11.77)	856(30.26)	
**Mother's Occupation**						
Home maker	961(93.76)	376(96.66)	701(94.22)	484(72.13)	2522(89.15)	
Daily wages	17(1.66)	3(0.77)	17(2.28)	171(25.48)	208(7.35)	
Salaried/Professionals	47(4.59)	9(2.31)	18(2.42)	7(1.04)	81(2.86)	<0.0001
Self-Employment	0(0.0)	1(0.26)	8(1.08)	9(1.34)	18(0.64)	
**Biomass fuel**						
Yes	211(20.59)	245(62.98)	263(35.35)	609(90.76)	1328(46.94)	<0.0001
No	814(79.41)	144(37.02)	481(64.65)	62(9.24)	1501(53.06)	
**Smoking Status-Father**						

For peer review only - http://bmjopen.bmj.com/site/about/guidelines.xhtml

Yes	152(14.83)	45(11.57)	56(7.53)	59(8.79)	312(11.03)	<0.0001
No	873(85.17)	344(88.43)	688(92.47)	612(91.21)	2517(88.97)	
**Indoor smoking-Father**						
Yes	83(8.10)	21(5.40)	16(2.15)	43(6.41)	163(5.76)	<0.0001
No	942(91.90)	368(91.60)	728(97.85)	628(93.59)	2666(94.24)	
**Smoking Status-Family member**						
Yes	129(12.59)	55(14.14)	45(6.05)	102(15.20)	331(11.70)	<0.0001
No	896(87.41)	334(85.86)	699(93.95)	569(84.80)	2498(83.30)	
**Indoor smoking – Family member**						
Yes	84(8.20)	27(6.94)	27(3.63)	94(14.01)	232(8.20)	<0.0001
No	941(91.80)	362(93.06)	717(96.37)	577(85.99)	2597(91.80)	
**Clinical Variables at the time of admission at hospital**	**n, Mean± SD**	**n, Mean± SD**	**n, Mean± SD**	**n, Mean± SD**	**n, Mean± SD**	**p value**
Age (months)	1025, 14.53±13.88	389, 10.69±10.95	744, 10.26±11.35	671, 12.30±13.29	2829, 12.35±12.85	<0.0001
Height (cm)	303, 68.61±13.78	324, 70.66±13.75	34, 64.38±10.25	266, 70.46±12.14	927, 69.70±13.26	<0.018
Weight (Kg)	1025, 7.96±2.97	389, 7.34±2.73	744, 7.11±2.78	671, 7.78±2.93	2829, 7.61±2.90	<0.0001
Fever Duration (days)	929, 4.46±2.71	321, 3.59±2.37	689, 4.25±2.52	569, 3.54±2.47	2508, 4.08±2.59	<0.0001
Oxygen saturation done n (%)	528(51.51)	343(88.17)	236(34.25)	319(56.06)	1426(50.40)	<0.0001
Oxygen Saturation ≤90 % n (%)	90 (17.04)	86 (26.79)	76(32.20)	49(15.36)	301(20.58)	<0.0001

Table 2 shows proportions of radiological pneumonia among cases hospitalized for CAP in four participating districts. We observed statistically significant district-wise heterogeneity in radiological abnormalities. We found higher proportion of radiological abnormalities in Patna and Lucknow districts, which have a larger urban population, and lower proportion in Etawah and Darbhanga districts, which in contrast have a larger rural population. We also observed correspondingly higher proportion of PEP alone or with other infiltrates in districts of Lucknow and Patna and lower in districts of Etawah and Darbhanga.

For peer review only

For peer review only - http://bmjopen.bmj.com/site/about/guidelines.xhtml

Table 2 also describes univariate distribution of socio-demographic and clinical factors of CAP among hospitalized children (2-59 months). Statistically significantly higher proportion of females hospitalized for CAP had radiologically abnormal CXR. Likewise, statistically significantly higher proportion of abnormal versus normal CXRs findings were reported in hospitalized cases who had symptoms of fever, pallor, wheezing on auscultation, vomiting everything or were malnourished.

Table 2: Distribution of socio-demographic and clinical factors by chest radiograph findings among hospitalized children from January 2015-April 2017

	Interpretable chest X rays				Abnormal chest X rays		
	**N=2829**	**Normal 1852 n (%)**	**Abnormal 977 n (%)**	**p value**	**PEP* alone or with other infiltrate 635 n (%)**	**Other infiltrates 342 n (%)**	**p value**
**Participating site** (row %)							
Lucknow	1025	636 (62.05)	389 (37.95)	<0.0001	282 (72.49)	107 (27.51)	<0.0001
Etawah	389	275 (70.69)	114 (29.31)		73 (64.04)	41 (35.96)	
Patna	744	457 (61.42)	287 (38.58)		184 (64.11)	103 (35.89)	
Darbhanga	671	484 (72.13)	187 (27.87)		96 (51.34)	91 (48.66)	
**Socio-demographic & clinical factors** (column %)							
**Age-group (months)**							
2-11	1865	1223 (66.04)	642 (65.71)	0.86	409 (64.41)	233 (68.13)	0.26
12-59	964	629 (33.96)	335 (34.29)		226 (35.59)	109 (31.87)	
**Gender**							

For peer review only

For peer review only - http://bmjopen.bmj.com/site/about/guidelines.xhtml

Male	2005	1354 (73.11)	651 (66.63)	<0.0001	426 (67.09)	225 (65.79)	0.72
Female	824	498 (26.89)	326 (33.37)		209 (32.91)	117 (34.21)	
**Place of residence**							
Rural	1392	921 (49.73)	471 (48.21)	0.44	299 (47.09)	172 (50.29)	0.34
Urban	1437	931 (50.27)	506 (51.79)		336 (52.91)	170 (49.71)	
**Biomass fuel**							
Yes	1501	867 (46.81)	461 (47.19)	0.44	294 (42.30)	167 (48.83)	0.24
No	1328	985 (53.19)	516 (52.81)		341 (53.70)	175 (51.17)	
**Immunization status**							
Complete for age	2347	1546 (83.48)	801 (81.99)	0.32	516 (81.26)	285 (83.33)	0.54
Incomplete	482	306 (16.52)	176 (18.01)		119 (18.74)	57 (16.67)	
**Symptoms of pneumonia**							
Fever	2499	1616 (87.26)	883 (90.38)	0.014	575 (90.55)	308 (90.06)	0.82
Cyanosis	62	39 (2.11)	23 (2.35)	0.34	16 (2.52)	7 (2.05)	0.64
Pallor	764	465 (25.11)	299 (30.60)	0.002	200 (31.50)	99 (28.95)	0.41
Wheeze on auscultation	2054	1377 (74.35)	677 (69.29)	0.005	415 (65.35)	262 (76.61)	0.0003
Duration of illness fever [days] (n, Mean ± SD)	2499	1611 3.91±2.51	888, 4.40±2.70	<0.0001	577, 4.57±2.82	342, 4.08±2.44	0.011
**Respiratory Rate and Fast Breathing**							
Respiratory Rate [2-11 months] (n, Mean ± SD)	1865	1243, 55.52±11.29	642, 57.99±11.70	<0.0001	409, 58.12±11.88	233, 57.74±11.40	0.69
Respiratory Rate [12-59 months] (n, Mean ± SD)	964	629, 49.78±12.41	335, 51.28±13.37	0.08	226, 51.35±13.31	109, 51.12±13.35	0.88
Fast Breathing for age (2-11 months)	1735	1130 (61.02)	605 (61.92)	0.11	384 (60.47)	221 (64.62)	0.69
Fast Breathing for	862	562	300	0.92	204	96	0.53

For peer review only

For peer review only - http://bmjopen.bmj.com/site/about/guidelines.xhtml

age (12-59 months)		(30.35)	(30.71)		(32.13)	(28.07)	
**Danger Signs of pneumonia**							
Lethargy	1101	732 (39.52)	369 (37.77)	0.39	247 (38.90)	122 (35.67)	0.33
Inability to drink	937	612 (33.05)	325 (33.27)	0.46	211 (33.23)	114 (33.33)	0.97
Convulsion	148	98 (5.29)	50 (5.12)	0.93	33 (5.20)	17 (4.97)	0.87
Cyanosis	42	27 (1.46)	15 (1.54)	0.87	12 (1.89)	3 (0.88)	0.28
**Malnutrition Status**							
Normal *	1880	1293 (69.82)	587 (60.08)		367 (57.80)	220 (64.33)	
Malnutrition*	517	333 (17.98)	184 (18.83)	< 0.0001	122 (19.21)	62 (18.13)	0.06
Severe malnutrition*	432	226 (12.20)	206 (21.08)		146 (22.99)	60 (17.54)	

*Normal-weight of age z score > -2SD; Malnutrition-weight-for-age z ≤ -2SD and Severe malnutrition-weight-for- age z ≤ -3SD

Table 3 describes four multivariate unconditional logistic regression models to find associates of abnormal CXR findings. After controlling for age, gender, symptoms of pneumonia, duration of illness, biomass fuel and malnutrition status of cases, statistically significant district-wise heterogeneity remained in the first three models. Models I, II and III had similar associates for radiological abnormalities whereas Model IV was different. Across all the four models, female gender and those with severe malnutrition had statistically significantly higher risk for having abnormal CXRs. A higher risk of radiological abnormalities was also observed in those children with longer duration of illness.

For peer review only - http://bmjopen.bmj.com/site/about/guidelines.xhtml

Table 3: Independent Associations between Chest Radiograph Findings and demographic and clinical factors, among hospitalized children January 2015-April 2017

Variables	Model – I Abnormal/Normal Ref		Model – II PEP alone or with other infiltrate /Normal Ref		Model – III PEP alone / Normal Ref		Model – IV Other infiltrate / Normal Ref	
	Adjusted Odd Ratio (95%CI)	p value	Adjusted Odd Ratio (95%CI)	p value	Adjusted Odd Ratio (95%CI)	p value	Adjusted Odd Ratio (95%CI)	p value
**Districts**								
Lucknow vs. Others	1.58 (1.20-2.10)	<0.0001	2.07 (1.48-2.89)	<0.0001	2.20 (1.52-3.19)	<0.0001	0.98 (0.65-1.47)	0.93
Etawah vs. Others	1.22 (0.88-1.70)	0.23	1.30 (0.87-1.95)	0.19	1.49 (0.95-2.30)	0.07	1.17 (0.74-1.87)	0.50
Patna vs. Others	1.67 (1.27-2.20)	<0.0001	1.89 (1.36-2.64)	<0.0001	2.25 (1.56-3.24)	<0.0001	1.39 (0.95-2.07)	0.09
**Age – Group (months)**								
2-11 Ref								
12-59	0.92 (0.77-1.10)	0.34	0.95 (0.77-1.17)	0.62	1.03 (0.82-1.29)	0.79	0.86 (0.66-1.13)	0.27
**Gender**								
Male Ref								
Female	1.39 (1.16-1.66)	<0.0001	1.34 (1.08-1.65)	0.008	1.28 (1.01-1.61)	0.03	1.48 (1.14-1.92)	0.004
**Symptoms of pneumonia¶**								
Wheezing	0.83 (0.68-1.01)	0.06	0.72 (0.57-0.90)	0.005	0.75 (0.59-0.96)	0.02	1.14 (0.83-1.55)	0.42
Pallor	1.30 (1.08-1.58)	0.006	1.28 (1.03-1.60)	0.02	1.22 (0.95-1.55)	0.12	1.34 (1.01-1.77)	0.04
Vomiting everything	0.90 (0.75-1.09)	0.28	0.80 (0.64-0.99)	0.04	0.78 (0.62-1.01)	0.05	1.09 (0.83-1.08)	0.51
**Duration of illness, fever (days)**	1.06 (1.04-1.09)	<0.0001	1.08 (1.04-1.12)	<0.0001	1.08 (1.04-1.12)	<0.0001	1.03 (0.98-1.48)	0.24
**Biomass fuel**	1.28 (1.05-1.57)	0.02	1.39 (1.10-1.76)	0.006	1.40 (1.14-1.88)	0.003	1.08 (0.79-1.45)	0.64
**Malnutrition Status**								
Normal *Ref								
Malnutrition*	1.18 (0.93-1.45)	0.15	1.17 (0.91-1.52)	0.23	1.17 (0.88-1.55)	0.27	1.12 (0.82-1.52)	0.47
Severe malnutrition*	1.65 (1.31-2.09)	<0.0001	1.82 (1.34-2.36)	<0.0001	1.87 (1.41-2.47)	<0.0001	1.62 (1.71-2.23)	0.003

Abbreviations used: Ref Reference Category

Footnotes: ¶ No signs of pneumonia taken as a reference
*Normal: weight-for-age z score> -2SD; Malnutrition: weight-for -age z ≤ -2SD; Severe malnutrition: weight-for-age z ≤ -3SD

For peer review only

For peer review only - http://bmjopen.bmj.com/site/about/guidelines.xhtml

DISCUSSION

This prospective hospital-based surveillance study was conducted to assess the radiological abnormalities in children (2-59 months) residing in pre-specified districts of Uttar Pradesh and Bihar, India and hospitalized with CAP. The study was conducted from January 2015 to April 2017, prior to introduction of PCV in the national immunization programme of the Government of India[9].

In our study, among interpretable CXRs, we found 22.44% (635/2829) children had PEP alone or with infiltrates, other infiltrates only 12.09%(342/2829) and 65.46% (1852/2829) had normal CXRs findings. Our study used WHO case definition for CAP [19]. A panel of three trained radiologists interpreted CXRs, adopting WHO recommended methodology [11 12]. These make our study methodology robust and results generalizable.

There were 88.54% (2829/3195) interpretable CXRs in the current study. This is similar to 83% (3587/3973) interpretable CXRs reported by Pneumonia Etiology Research for Child Health (PERCH) study conducted on 4232 children (1-59 months) to assess the etiology of CAP in nine sites of seven countries [27]. Consistent with PERCH findings, a vaccine probe trial conducted in Gambia found the proportion of interpretable CXRs among unvaccinated cases of pneumonia to be 84.32% (242/287)[28].

There have been several studies in the past two decades, which have reported CXRs findings in hospitalized cases of CAP. Almost all of these were conducted before the introduction of PCV in their respective regions. A small prospective study conducted in Ethiopia reported radiological

For peer review only - http://bmjopen.bmj.com/site/about/guidelines.xhtml

abnormality in CXRs in 48.3% (95% CI 39.49-57.22) among 122 children (3 months-14 years) clinically diagnosed with WHO-defined severe pneumonia[29]. Similar findings were reported from the Gambian vaccine probe trial where the proportion of radiological abnormality was 45% (95% CI: 43.35-46.46) among unvaccinated hospitalized cases of clinical pneumonia[28]. PERCH study found that 54% (95% CI: 52.31-55.57) of CXRs among cases of CAP were abnormal [27]. In all of these studies, proportion of cases with abnormal CXRs is higher than 34.5% (95% CI 32.8-36.3) found by us in the current study. However, our findings are similar to PERCH rural study site of Matlab, Bangladesh that reported radiological abnormality in 35.3% (95% CI: 29.77-40.85) CXRs of hospitalized cases of CAP [27]. Another PERCH urban site of Dhaka, Bangladesh reported 63.10% (95% CI 56.18 -70.02) cases with abnormal CXRs [27]. In our study, radiological abnormalities in CXRs were higher in cases from largely urban districts of Patna and Lucknow compared to rural districts of Darbhanga and Etawah. This is consistent with rural-urban differences in Bangladesh sites of PERCH. Variation in CXR findings among cases of CAP may be due to infecting organism, immune response of patient and prior duration of disease.

In 2016, WHO's Department of Immunization, Vaccines and Biologicals standardized the categorization of radiological pneumonia and established that PEP can be taken as a good surrogate marker of SP in epidemiological and vaccine efficacy studies[12]. In our study, 22.44 % (95% C.I. 20.90 -23.98), CXRs were having PEP alone or with other infiltrates. This is similar to PERCH study that reported PEP alone or with other infiltrates in 27% (95% C.I. 25.50 -28.40) hospitalized cases of CAP[27]. Another study conducted in Gambia reported that 45% (95% CI: 43.35-46.46) non-vaccinated children had PEP and/or other infiltrates [28]. PEP has been

For peer review only - http://bmjopen.bmj.com/site/about/guidelines.xhtml

associated with increased risk of treatment failure (p=0.002), increased length of hospitalization (p=0.0003) and more days of respiratory support (p=0.002) in Botswana when compared with cases reporting` no significant pathology` on CXRs[30].

In our study, female gender (p<0.001) was at the higher risk of developing radiological abnormalities compared to males (**table 3**). The results are in concordance with a hospital-based case-control study carried out in Brazil that reported male gender as a protective factor against pneumonia (OR = 0.53; 95 % CI 0.39–0.72) [31]. Another study in Mozambique, Africa reported that male gender was not significantly associated with presence of radiological abnormalities (OR =0.77; 95 % CI 0.56–1.05) in children (0-59 months) suffering from severe pneumonia [32]. However, in contrast, a Gambian study reported male preponderance for all pneumonia that was most marked for `other infiltrates/abnormalities` pneumonia[28].

In our study, it was observed that there was differential care-seeking by gender for CAP in all four project sites. Although females admitted with CAP were at higher risk of having radiological abnormalities, lower proportion were hospitalized for it. Gender inequality in health care seeking for females is common in India, as in other South Asian countries [33] [34]. Since there is no health-care financing or health insurance provision in India, in case of severe illness, parents are less likely to incur out-of-pocket expenditure or incur debts to pay expenses on medical treatment of their daughters compared to sons [35]. Another Indian study found that male children were five times more likely to be taken early for medical care and three times more likely to be seen by qualified medical doctors compared to female children [36]. We also found

For peer review only - http://bmjopen.bmj.com/site/about/guidelines.xhtml

that majority of hospitalized cases of pneumonia were from urban areas, in contrast with observations of other researchers who report poor health care seeking from rural areas[37].

A systematic review with meta-analysis conducted in 2019 suggests that no one clinical feature is sufficient on its own to diagnose radiological pneumonia [38]. However other socio-demographic and clinical correlates of abnormal CXRs found by us (Model 1), which increased the risk of radiological abnormalities, were presence of pallor, severe malnutrition, longer duration of illness and exposure to biomass fuel. Exposure to biomass fuel used for cooking is an important factor that increases the risk of CAP in developing countries[39]. In rural India, majority of the households use biomass fuel like firewood, dung cakes and wood for cooking [40]. Young children are at risk to adverse effects of exposure to biomass fuel as either the households have no separate cooking space or have poor ventilation and sometimes young children stay with their mother while she cooks.

Other correlates of PEP/Radiological Pneumonia (Models II and III) possibly due to SP, besides those found in Model I, were presence of vomiting everything and wheeze on auscultation, both of which were found to be protective. These symptoms/signs are more often reported in viral pneumonia[41]. Correlates of radiological abnormalities of `other infiltrates` (Model IV), which increased the risk, were again female gender, pallor and severe malnutrition. Hence it is difficult to attribute radiological findings of other infiltrates to either bacterial or viral etiology.

Based on our study, almost two-third hospitalized cases of CAP had normal CXRs and this could be perhaps of viral etiology. This is supported by a recent study that reported 61.4% (95% CI

For peer review only - http://bmjopen.bmj.com/site/about/guidelines.xhtml

57·3–65·6) cases to be viral[41]. Among one-third of cases of CAP had abnormal CXRs and thus were more likely to be bacterial in etiology, two-thirds of which were possibly due to SP.

In India, 13-valent PCV is given using a three dose schedule (2 primary and one booster) at 6 weeks, 14 weeks and 9 months of age. PCV 13 provides coverage against 13 serotypes (1, 3, 4, 5, 6A, 6B, 7F, 9V, 14, 18C, 19A, 19F and 23F) causing pneumococcal pneumonia[42]. Several studies have assessed serotype distribution of pneumococcal disease among children in India. A study conducted in Vellore, India found that the most common serotypes causing invasive infections among under-five children were 14, 19F, 5, 6A and 6B, all of which were covered by the 13-valent PCV [43]. Another population-based surveillance study conducted in rural Bangladesh found that the most common serotypes were 1, 5, 14, 18C and 19A and 38 and these comprised more than three-fourth of the *S. pneumoniae* isolates [44]. A systematic review and meta-analysis of data collected on Invasive Pneumococcal Disease serotypes from under-five children during the pre-PCV period (between 1980–2007) found that serotypes included in both the 10-valent and 13-valent PCVs accounted for 10 million cases and 600,000 deaths worldwide[45].

Several strengths of the study are worth-noting. This was a prospective, multi-site study where recruitments were done from a large hospital surveillance network established especially for the study in four districts of two Indian states that have high under-five mortality rates. Standard WHO definition was used to identify hospitalized cases of CAP. Radiological abnormalities were interpreted by a panel of three trained radiologists at locations out of the surveillance network, blinded to each other as well as clinical features of the case. Despite these strengths,

For peer review only - http://bmjopen.bmj.com/site/about/guidelines.xhtml

our study findings have certain limitations. First, in our study, pre-existing x-rays machines which were not of uniform specification, were used. This might have caused variation in quality of CXR images, though this error was minimized by digitizing the CXR images centrally. Secondly, in our study, clinical data collection was recorded by physicians in the network hospitals and this could be subject to observer bias. However, the primary outcome of the study was radiological findings of CXRs of cases admitted with CAP. This was not subject to bias. In this study, we have not collected information on use of antibiotic prior to hospitalization; as such information is not available reliably. However, in another study, done in one of the network hospitals of Lucknow in the recent past, it was found that 70.5% children tested positive for antibiotics on urine examnation[46].

CONCLUSION

Among hospitalized cases of community-acquired pneumonia, almost one-third children had abnormal chest radiographs of which about two-thirds had abnormalities related with possible bacterial etiology (SP). Hence, the introduction of pneumococcal vaccination is likely to reduce the burden of childhood pneumonia in India. Since the study was done prior to the introduction of PCV in India, continued surveillance will be required to assess the impact of PCV on radiological findings in cases admitted with CAP. The impact of introduction of PCV in the national immunization programme on under-five mortality rate and burden of CAP needs to be assessed.

For peer review only - http://bmjopen.bmj.com/site/about/guidelines.xhtml

Acknowledgements: We thank the state governments of Uttar Pradesh and Bihar for giving us permission to conduct the study. We extend our gratitude to participating tertiary and secondary care hospitals for their contribution in the study. We offer thanks to Dr. Kamrun Nahar from the International Centre for Diarrhoeal Disease Research, Bangladesh for imparting training to the panel of study radiologists. Members of the Lucknow Community-Acquired Pneumonia (CAP) Group: King George's Medical University (KGMU), Lucknow, India: Prof. Shally Awasthi, Department of Pediatrics, Prof. Neera Kohli, Department of Radio-diagnosis, Prof. Monica Agarwal, Department of Community Medicine; Mr. Puneet Dhasmana, Project Staff; Patna Medical College and Hospital, Patna, Bihar-Prof. Neelam Verma and Prof. Chandra Bhushan Kumar, Department of Pediatrics; Darbhanga Medical College and Hospital, Darbhanga, Bihar, India- Prof. Chittaranjan Roy, Department of Community Medicine, Prof. Kripanath Mishra, Department of Pediatrics; Uttar Pradesh University of Medical Sciences, Etawah, Uttar Pradesh, India- Prof. Pankaj Kumar Jain, Department of Community Medicine, Prof. Rajesh Yadav, Department of Pediatrics.

Contributors: The study was conceived and designed by SA. CAP study group performed data acquisition.CMP and NM[3]conducted the statistical analysis of the data. The paper was written by SA, TR, MA and CMP. AC, NM[5], RCS and NK interpreted chest x-rays. All authors were involved with drafting and revising the work and approved the final submission.

Funding statement: The study was supported by Bill & Melinda Gates Foundation (https://www.gatesfoundation.org/) via Grant No: OPP1118005**.** Funding agency had no role in the design of the study and collection, analysis and interpretation of data, or in writing the manuscript.

Competing interests: None declared.

For peer review only - http://bmjopen.bmj.com/site/about/guidelines.xhtml

Patient consent for publication: Not required.

Ethics approval: The Institutional Ethics Committee of King George's Medical University (Lucknow), The Uttar Pradesh University of Medical Sciences (Etawah), Patna Medical College and Hospital (Patna) and Darbhanga Medical College and Hospital (Darbhanga) gave ethical approval for the conduct of study.

Provenance and peer review: Not commissioned; externally peer reviewed.

Data sharing statement: The data contained within this study can be obtained by writing to shally07@gmail.com

Copyright information: This is an Open Access article distributed in accordance with the Creative Commons Attribution Non Commercial (CC BY-NC 4.0) license, which permits others to distribute, remix, adapt, build upon this work non-commercially, and license their derivative works on different terms, provided the original work is properly cited and the use is non-commercial. See: http://creativecommons.org/licenses/by-nc/4.0/

Supporting Information

S1 Appendix: Chest radiograph interpretation form

REFERENCES:

1. UNICEF U. Levels and trends in child mortality. Estimates developed by the UN inter-agency group for child mortality estimation. 2011.
2. Amouzou A, Velez LC, Tarekegn H, *et al*. One is too many: ending child deaths from pneumonia and diarrhoea. New York: Unicef, 2016. pp 73–75

For peer review only - http://bmjopen.bmj.com/site/about/guidelines.xhtml

3. Mathew JL, Patwari AK, Gupta P, *et al*. Acute respiratory infection and pneumonia in India: a systematic review of literature for advocacy and action: UNICEF-PHFI series on newborn and child health, India. *Indian Pediatr*2011; 48: 191–218.

4. Farooqui H, Jit M, Heymann DL, *et al*. Burden of severe pneumonia, pneumococcal pneumonia and pneumonia deaths in Indian states: modelling based estimates. *PLoS One* 2015

5. Rudan I, O'brien KL, Nair H,*et al*. Epidemiology and etiology of childhood pneumonia in 2010: estimates of incidence, severe morbidity, mortality, underlying risk factors and causative pathogens for 192 countries. *Journal of global health*. 2013 Jun;3(1).

6. Leung DT, Chisti MJ, Pavia AT. Prevention and control of childhood pneumonia and diarrhea. *Pediatric Clinics*. 2016 Feb 1;63(1):67-79.

7. Virkki R, Juven T, Rikalainen H, *et al*.Differentiation of bacterial and viral pneumonia in children. *Thorax*. 2002 May 1;57(5):438-41.

8. World Health Organization. Pneumococcal conjugate vaccines in infants and children under 5 years of age: WHO position paper–February 2019. Accessed 12th July 2019.

9. Government of India. Introduction of Pneumococcal Conjugate Vaccine: Operational Guidelines. Ministry of Health and Family Welfare, 2017 Available at: https://nhm.gov.in/New_Updates_2018/NHM_Components/Immunization/Guildelines_for_immunization/Operational_Guidelines_for_PCV_introduction.pdf. Assessed 12 July 2019.

10. Cherian T, Mulholland EK, Carlin JB,*et al*. Standardized interpretation of paediatric chest radiographs for the diagnosis of pneumonia in epidemiological studies. *Bulletin of the World Health Organization*. 2005;83:353-9

For peer review only

For peer review only - http://bmjopen.bmj.com/site/about/guidelines.xhtml

11. Pneumonia Vaccine Trial Investigators' Group. Standardization of Interpretation of Chest Radiographs for the Diagnosis of Pneumonia in Children. Geneva: Department of Vaccines and Biologicals, World Health Organization; 2001

12. Mahomed N, Fancourt N, De Campo J, *et al*. Preliminary report from the World Health Organization chest radiography in epidemiological studies project. *Pediatric radiology*. 2017 Oct 1;47(11):1399-404.

13. Greenberg D, Givon-Lavi N, Ben-Shimol S,*et al*. Impact of PCV7/PCV13 introduction on community-acquired alveolar pneumonia in children< 5 years. *Vaccine*. 2015 Aug 26;33(36):4623-9. 6.

14. Sutcliffe CG, Shet A, Varghese R, *et al*. Nasopharyngeal carriage of Streptococcus pneumoniae serotypes among children in India prior to the introduction of pneumococcal conjugate vaccines: a cross-sectional study. *BMC Infect Dis*. 2019 Dec;19(1):605.

15. Annual health survey (AHS) factsheet 2012–13 Uttar Pradesh [Internet]. New Delhi: Office of Registrar General & Census Commissioner, Ministry of Home Affairs, Government of India; 2010–2011. Available from:http://www.censusindia.gov.in/vital_statistics/AHSBulletins/AHS_Factsheets_2012-13/FACTSHEET-UTTAR_PRADESH.pdf. Assessed 5th August, 2019.

16. Annual health survey (AHS) factsheet 2012–13 Bihar [Internet]. New Delhi: Office of Registrar General & Census Commissioner, Ministry of Home Affairs, Government of India; 2010–2011. Available from:http://www.censusindia.gov.in/vital_statistics/AHSBulletins/AHS_Factsheets_2012-13/FACTSHEET-Bihar.pdf. Assessed 5th August, 2019.

For peer review only

17. Awasthi S, Pandey CM, Verma T, *et al*. Incidence of community-acquired pneumonia in children aged 2-59 months of age in Uttar Pradesh and Bihar, India, in 2016: An indirect estimation. *PloS one*. 2019 Mar 20;14(3):e0214086.

18. Awasthi S, Singh JV, Kohli N, *et al*. Hospital-based surveillance for radiological pneumonia in children under 5 years of age in Uttar Pradesh and Bihar. *Pediatric Infectious Disease*. 2016 Jun 30;8(2):52-7.

19. World Health Organization. Pocket Book of Hospital Care for Children: Guidelines for the Management of Common Childhood Illnesses. 2nd edn. Geneva, Switzerland: World Health Organization, 2013.

20. Charan J, Biswas T. How to calculate sample size for different study designs in medical research?. Indian journal of psychological medicine. 2013 Apr;35(2):121.

21. Integrated Management of Childhood Illness (IMCI) Chart Booklet [online] Geneva: World Health Organization. Available at: https://apps.who.int/iris/bitstream/handle/10665/104772/9789241506823_Chartbook_eng.pdf Accessed:19th December, 2019.

22. Bassignani MJ, Bubash-Faust L, Ciambotti J, *et al*. Conversion of teaching file cases from film to digital format: a comparison between use of a diagnostic-quality digitizer and use of a flatbed scanner with transparency adapter. *Acad Radiol*. 2003 May 1;10(5):536-42.

23. World Health Organization. The WHO Anthro Survey Analyser. In: World Health Organization [Internet]. Available: https://www.who.int/nutgrowthdb/software/en/. Accessed: 10th June, 2019.

For peer review only - http://bmjopen.bmj.com/site/about/guidelines.xhtml

24. Horton NJ, Kleinman KP. Much ado about nothing: A comparison of missing data methods and software to fit incomplete data regression models. The American Statistician. 2007 Feb 1;61(1):79-90.

25. SPSS Inc. Released 2009. SPSS for Windows [software], Version 22·0. Chicago, SPSS Inc. Available from:www.spss.com

26. von Elm E, Altman DG, Egger M, et al. The strengthening the reporting of observational studies in epidemiology (STROBE) statement: guidelines for reporting observational studies. J Clin Epidemiol 2008;61:344–9

27. Fancourt N, Deloria Knoll M, Baggett HC, *et al*. Chest radiograph findings in childhood pneumonia cases from the multisite PERCH study. *Clin Infect Dis*. 2017 May 29;64(suppl_3):S262-70

28. Enwere G, Cheung YB, Zaman SM, *et al*. Epidemiology and clinical features of pneumonia according to radiographic findings in Gambian children. *Trop Med Int Health*. 2007 Nov;12(11):1377-85.

29. Meiron Hassen, Alemayehu Toma, Mulugeta Tesfay, *et al*. Radiologic Diagnosis and Hospitalization among Children with Severe Community Acquired Pneumonia: A Prospective Cohort Study. *Biomed Res Int*, vol. 2019, Article ID 6202405, 8 pages, 2019. https://doi.org/10.1155/2019/6202405.

30. Kelly MS, Crotty EJ, Rattan MS, *et al*. Chest radiographic findings and outcomes of pneumonia among children in Botswana. *Pediatr Infect Dis J*. 2016 Mar;35(3):257.

31. da Fonseca Lima EJ, Mello MJ, Lopes MI, *et al*. Risk factors for community-acquired pneumonia in children under five years of age in the post-pneumococcal conjugate vaccine era in Brazil: a case control study. *BMC Pediatr*. 2016 Dec;16(1):157.

For peer review only

For peer review only - http://bmjopen.bmj.com/site/about/guidelines.xhtml

32. Sigauque B, Roca A, Bassat Q, *et al*. Severe pneumonia in Mozambican young children: clinical and radiological characteristics and risk factors. *J Trop Pediatr*. 2009 Apr 28;55(6):379-87.

33. Ismail SA, McCullough A, Guo S, *et al*. Gender-related differences in care-seeking behaviour for newborns: a systematic review of the evidence in South Asia. BMJ global health. 2019 May 1;4(3):e001309.

34. Fikree FF, Pasha O. Role of gender in health disparity: the South Asian context. BMJ 2004;328:823–6. 10.1136/bmj.328.7443.823

35. Asfaw A, Lamanna F, Klasen S. Gender gap in parents' financing strategy for hospitalization of their children: evidence from India. Health economics. 2010 Mar;19(3):265-79.

36. Pandey A, Sengupta PG, Mondal SK, *et al*. Gender differences in healthcare-seeking during common illnesses in a rural community of West Bengal, India. Journal of Health, Population and Nutrition. 2002 Dec 1:306-11.

37. Jana A, Basu R. Examining the changing health care seeking behavior in the era of health sector reforms in India: evidences from the National Sample Surveys 2004 & 2014. Global health research and policy. 2017 Dec;2(1):6.

38. Rambaud-Althaus C, Althaus F, Genton B, *et al*. Clinical features for diagnosis of pneumonia in children younger than 5 years: a systematic review and meta-analysis. *Lancet Infect Dis*. 2015 Apr 1;15(4):439-50

39. Awasthi S, Glick AH, Fletcher RH. Effect of cooking fuels on Respiratory Diseases in Pre-school children in Lucknow, India. *Am J Trop Med Hyg* 1996; 55: 48-51

For peer review only - http://bmjopen.bmj.com/site/about/guidelines.xhtml

40. Tielsch JM, Katz J, Thulasiraj RD, *et al*. Exposure to indoor biomass fuel and tobacco smoke and risk of adverse reproductive outcomes, mortality, respiratory morbidity and growth among newborn infants in south India. *Int J Epidemiol*. 2009 Sep 16;38(5):1351-63.

41. O'Brien KL, Baggett HC, Brooks WA, *et al*. Causes of severe pneumonia requiring hospital admission in children without HIV infection from Africa and Asia: the PERCH multi-country case-control study. *The Lancet*. 2019 Jun 27

42. World Health Organization. Pneumococcal conjugate vaccines in infants and children under 5 years of age: WHO position paper–February 2019. Accessed 12th March. 2019.

43. Balaji V, Jayaraman R, Verghese VP, Baliga PR, Kurien T. Pneumococcal serotypes associated with invasive disease in under five children in India & implications for vaccine policy. The Indian journal of medical research. 2015 Sep;142(3):286.

44. Arifeen SE, Saha SK, Rahman S, Rahman KM, Rahman SM, Bari S, Naheed A, Mannan I, Seraji MH, Ahmed NU, Shameem Hassan M. Invasive pneumococcal disease among children in rural Bangladesh: results from a population-based surveillance. Clinical Infectious Diseases. 2009 Mar 1;48 (Supplement_2):S103-13.

45. Johnson HL, Deloria-Knoll M, Levine OS, Stoszek SK, Hance LF, Reithinger R, Muenz LR, O'Brien KL. Systematic evaluation of serotypes causing invasive pneumococcal disease among children under five: the pneumococcal global serotype project. PLoS medicine. 2010 Oct 5;7(10):e1000348

46. Bénet T, Picot VS, Awasthi S, Pandey N, Bavdekar A, Kawade A, Robinson A, Rakoto-Andrianarivelo M, Sylla M, Diallo S, Russomando G. Severity of pneumonia in under 5-year-old children from developing countries: a multicenter, prospective, observational study. The American journal of tropical medicine and hygiene. 2017 Jul 12;97(1):68-76

For peer review only - http://bmjopen.bmj.com/site/about/guidelines.xhtml

Figure Legend

Figure 1: Flow diagram of cases of community acquired pneumonia recruited from participating districts before the introduction of pneumococcal conjugate vaccine (January 2015-April 2017)

For peer review only

For peer review only - http://bmjopen.bmj.com/site/about/guidelines.xhtml

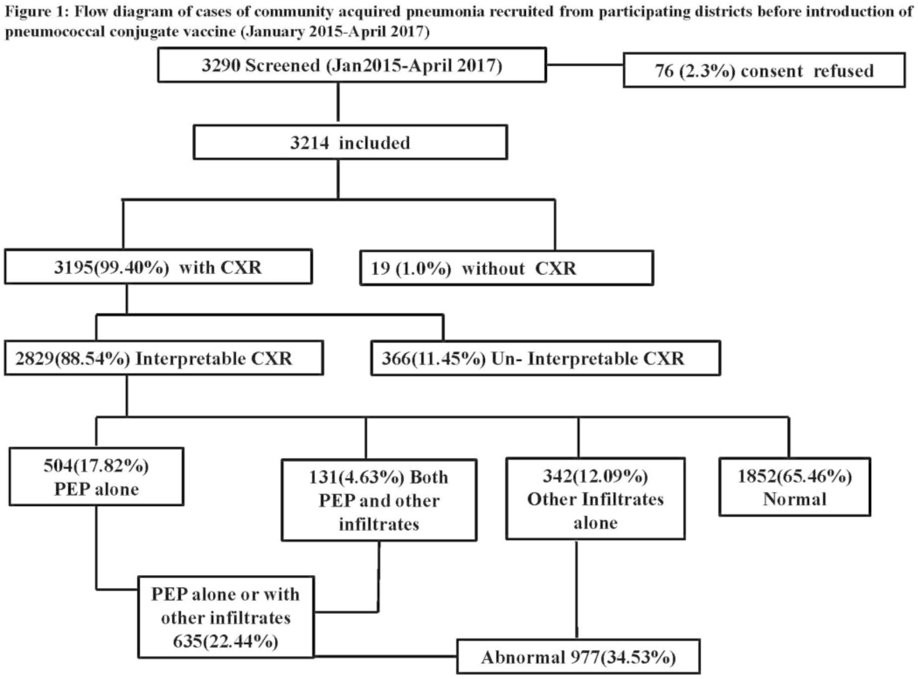

Figure 1: Flow diagram of cases of community acquired pneumonia recruited from participating districts before the introduction of pneumococcal conjugate vaccine (January 2015-April 2017)

254x190mm (300 x 300 DPI)

Childhood Pneumonia Surveillance

Department of Paediatrics, KGMU, Lucknow ,UP

Form-R

(RADIOLOGY REPORT FORM)

1	**Drs_ID**	[__][__][__][__]
	**Radiology Report**	**Patient Details**
2	**IDNo:**	[__] / [__] / [__][__][__]/[__][__][__][__]<br>State /District / Unit / Subject number<br>*(For office use)*
2	Date Of Report	[__][__]/[__][__]/[__][__][__][__] (DD/MM/YYYY)
	**Report Details**	**Findings (tick one)**
3	Image Quality	Adequate ☐ Suboptimal ☐ Un-interpretable ☐
4	Significant Pathology	Yes ☐ No ☐ Un-interpretable ☐
5	**End Point Consolidation**	
5a	Left	Yes ☐ No ☐ Un-interpretable ☐
5b	Right	Yes ☐ No ☐ Un-interpretable ☐<br>Uninterpretable
6	Other Infiltrates/Abnormalities	
6a	Left	Yes ☐ No ☐ Un-interpretable ☐
6b	Right	Yes ☐ No ☐ Un-interpretable ☐
7	Pleural Fluid	
7a	Left	Yes ☐ No ☐ Un-interpretable ☐
7b	Right	Yes ☐ No ☐ Un-interpretable ☐
8	Comments:	
9	Conclusion:	a) Primary endpoint pneumonia only ☐<br>b) Other infiltrate only ☐<br>c) Both PEP and other infiltrate ☐<br>d) Normal ☐<br>e) Un-interpretable for any findings☐

STROBE Statement—Checklist of items that should be included in reports of ***cross-sectional studies***

	Item No	Recommendation	Page Number
**Title and abstract**	1	(*a*) Indicate the study's design with a commonly used term in the title or the abstract	1
		(*b*) Provide in the abstract an informative and balanced summary of what was done and what was found	2-3
**Introduction**			
Background/rationale	2	Explain the scientific background and rationale for the investigation being reported	4
Objectives	3	State specific objectives, including any pre-specified hypotheses	5
**Methods**			
Study design	4	Present key elements of study design early in the paper	5
Setting	5	Describe the setting, locations, and relevant dates, including periods of recruitment, exposure, follow-up, and data collection	5
Participants	6	(*a*) Give the eligibility criteria, and the sources and methods of selection of participants	5-6
Variables	7	Clearly define all outcomes, exposures, predictors, potential confounders, and effect modifiers. Give diagnostic criteria, if applicable	5-6
Data sources/ measurement	8*	For each variable of interest, give sources of data and details of methods of assessment (measurement). Describe comparability of assessment methods if there is more than one group	7-8
Bias	9	Describe any efforts to address potential sources of bias	25
Study size	10	Explain how the study size was arrived at	6
Quantitative variables	11	Explain how quantitative variables were handled in the analyses. If applicable, describe which groupings were chosen and why	11-13
Statistical methods	12	(*a*) Describe all statistical methods, including those used to control for confounding	11-13
		(*b*) Describe any methods used to examine subgroups and interactions	Table 1, 2 &3
		(*c*) Explain how missing data were addressed	12 reference 24
		(*d*) If applicable, describe analytical methods taking account of sampling strategy	11-13
		(*e*) Describe any sensitivity analyses	NA
**Results**			
Participants	13*	(a) Report numbers of individuals at each stage of study—eg numbers potentially eligible, examined for eligibility, confirmed eligible, included in the study, completing follow-up, and analysed	14
		(b) Give reasons for non-participation at each stage	Figure 1
		(c) Consider use of a flow diagram	Figure 1
Descriptive data	14*	(a) Give characteristics of study participants (eg demographic, clinical, social) and information on exposures and potential confounders	14-15 Table 1
		(b) Indicate number of participants with missing data for each variable of interest	Figure 1

For peer review only

Outcome data	15*	Report numbers of outcome events or summary measures	
Main results	16	(*a*) Give unadjusted estimates and, if applicable, confounder-adjusted estimates and their precision (eg, 95% confidence interval). Make clear which confounders were adjusted for and why they were included	Table 3 (adjusted odds ratio)
		(*b*) Report category boundaries when continuous variables were categorized	Table 3
		(*c*) If relevant, consider translating estimates of relative risk into absolute risk for a meaningful time period	NA (calculated only odds ratio)
Other analyses	17	Report other analyses done—eg analyses of subgroups and interactions, and sensitivity analyses	Table 3
**Discussion**			
Key results	18	Summarise key results with reference to study objectives	21-23
Limitations	19	Discuss limitations of the study, taking into account sources of potential bias or imprecision. Discuss both direction and magnitude of any potential bias	25-26
Interpretation	20	Give a cautious overall interpretation of results considering objectives, limitations, multiplicity of analyses, results from similar studies, and other relevant evidence	21-23
Generalisability	21	Discuss the generalisability (external validity) of the study results	21
**Other information**			
Funding	22	Give the source of funding and the role of the funders for the present study and, if applicable, for the original study on which the present article is based	27

*Give information separately for exposed and unexposed groups.

Note: An Explanation and Elaboration article discusses each checklist item and gives methodological background and published examples of transparent reporting. The STROBE checklist is best used in conjunction with this article (freely available on the Web sites of PLoS Medicine at http://www.plosmedicine.org/, Annals of Internal Medicine at http://www.annals.org/, and Epidemiology at http://www.epidem.com/). Information on the STROBE Initiative is available at www.strobe-statement.org.

For peer review only

BMJ Open

Chest Radiograph Findings in children aged 2-59 months hospitalized with Community-Acquired Pneumonia, prior to the introduction of Pneumococcal Conjugate Vaccine in India- A Prospective Multisite Observational Study

Journal:	*BMJ Open*
Manuscript ID	bmjopen-2019-034066.R2
Article Type:	Original research
Date Submitted by the Author:	19-Mar-2020
Complete List of Authors:	Awasthi, Shally; King George's Medical University, Pediatrics<br>Rastogi, Tuhina; King George's Medical University, Paediatrics<br>Mishra, Neha; King George's Medical University, Pediatrics<br>Chauhan, Abhishek ; Dr Ram Manohar Lohia Institute of Medical Sciences, Department of Radio-diagnosis<br>Mohindra, Namita; Sanjay Gandhi Post Graduate Institute of Medical Sciences, Department of Radio-diagnosis<br>Shukla, Ram ; Banaras Hindu University, Varanasi, India, Institute of Medical Sciences<br>Agrawal, Monika; King George's Medical University, Social and Preventive Medicine<br>Pandey, CM; Sanjay Gandhi Post Graduate Institute of Medical Sciences, Biostatistics & Health Informatics<br>Kohli, Neera ; King George's Medical University, Department of Radio-diagnosis<br>Study Group, CAP; CAP study Group
**Primary Subject Heading**:	Paediatrics
Secondary Subject Heading:	Global health
Keywords:	Chest radiographs, Hospitalized community-acquired pneumonia, under-five, Streptococcus pneumoniae, India

SCHOLARONE™
Manuscripts

I, the Submitting Author has the right to grant and does grant on behalf of all authors of the Work (as defined in the below author licence), an exclusive licence and/or a non-exclusive licence for contributions from authors who are: i) UK Crown employees; ii) where BMJ has agreed a CC-BY licence shall apply, and/or iii) in accordance with the terms applicable for US Federal Government officers or employees acting as part of their official duties; on a worldwide, perpetual, irrevocable, royalty-free basis to BMJ Publishing Group Ltd ("BMJ") its licensees and where the relevant Journal is co-owned by BMJ to the co-owners of the Journal, to publish the Work in this journal and any other BMJ products and to exploit all rights, as set out in our licence.

The Submitting Author accepts and understands that any supply made under these terms is made by BMJ to the Submitting Author unless you are acting as an employee on behalf of your employer or a postgraduate student of an affiliated institution which is paying any applicable article publishing charge ("APC") for Open Access articles. Where the Submitting Author wishes to make the Work available on an Open Access basis (and intends to pay the relevant APC), the terms of reuse of such Open Access shall be governed by a Creative Commons licence – details of these licences and which Creative Commons licence will apply to this Work are set out in our licence referred to above.

Other than as permitted in any relevant BMJ Author's Self Archiving Policies, I confirm this Work has not been accepted for publication elsewhere, is not being considered for publication elsewhere and does not duplicate material already published. I confirm all authors consent to publication of this Work and authorise the granting of this licence.

For peer review only

Title: ***Chest Radiograph Findings in children aged 2-59 months hospitalized with Community-Acquired Pneumonia, prior to the introduction of Pneumococcal Conjugate Vaccine in India- A Prospective Multisite Observational Study***

Short Title: ***Radiological Findings in Children hospitalized with Community-Acquired Pneumonia in India Pre-Pneumococcal Conjugate Vaccine introduction***

Shally Awasthi[1]*, Tuhina Rastogi[1], Neha Mishra[1], Abhishek Chauhan[2], Namita Mohindra[3], Ram Chandra Shukla[4], Monika Agarwal[5], Chandra Mani Pandey[6], Neera Kohli[7] and CAP Group^

[1]Department of Pediatrics, King George's Medical University, Lucknow, India
[2]Department of Radio-diagnosis, Dr Ram Manohar Lohia Institute of Medical Sciences, Lucknow, India
[3]Department of Radio-diagnosis, Sanjay Gandhi Post Graduate Institute of Medical Sciences, Lucknow, India
[4]Department of Radio-diagnosis, Institute of Medical Sciences, Banaras Hindu University, Varanasi, India
[5] Department of Community Medicine, King George's Medical University, Lucknow, India
[6]Department of Biostatistics and Health Informatics, Sanjay Gandhi Postgraduate Institute of Medical Sciences, Lucknow, India
[7]Department of Radio-diagnosis, King George's Medical University, Lucknow, India

^Members of the Lucknow CAP Group are given in the Acknowledgement

*Corresponding Author
Postal Address: Prof. Shally Awasthi, Head, Department of Paediatrics, King George's Medical University, Lucknow , India Pincode: 226003
Mobile: +91-9839221244
E-mail: shally07@gmail.com

Word Count: 4643 Tables: 3 Figure:1

For peer review only - http://bmjopen.bmj.com/site/about/guidelines.xhtml

ABSTRACT

Objectives: To assess radiological abnormalities in chest X-rays and to identify the demographic and clinical correlates of specific radiological abnormalities in children aged 2-59 months, hospitalized with World Health Organization defined community-acquired pneumonia, residing in pre-specified districts of Uttar Pradesh and Bihar, India.

Design: Prospective, hospital-based surveillance

Setting: Multi-site study conducted in a network of 117 secondary/tertiary care hospitals in four districts of Uttar Pradesh and Bihar, India.

Participants: Included were children aged 2-59 months hospitalized with community-acquired pneumonia, residing in project district, with duration of illness of <14 days and who were not hospitalized elsewhere for this episode nor had been recruited previously.

Main outcome measure: Radiological abnormalities in the chest X-rays, where there was concordance between two or more of the panel of three trained radiologists.

Results: From January 2015 to April 2017, 3214 cases were recruited and in 99.40 % (3195/3214) chest X-rays were available. Among 88.54 % (2829/3195) interpretable X-rays, 34.53 % (977/ 2829, 95% C.I. 32.78 -36.28) had some radiological abnormalities, while the rest were normal. Primary endpoint pneumonia alone or with other infiltrates was found in 22.44 % (635/2829, C.I. 20.90 %-23.98 %), other infiltrates only in 12.09% (342/ 2829; C.I. 10.88 %-13.29 %). There was a statistically significant inter-district variation in radiological abnormality. Statistically significantly higher proportion of abnormal chest X-ray was found among girls, those with weight-for-age z score ≤-3 SD, longer duration of fever, pallor and with exposure to biomass fuel.

For peer review only - http://bmjopen.bmj.com/site/about/guidelines.xhtml

Conclusions: Among hospitalized cases of community-acquired pneumonia, almost one-third children had abnormal chest radiographs, which were higher in females, malnourished children and those with longer illnesses; and an intra-district variation was observed.

Key words: Chest radiographs, Hospitalized community-acquired pneumonia, under-five, *Streptococcus pneumoniae*, India

Strengths and Limitations of the Study

- Prospective, multisite recruitments from a large hospital surveillance network established for the project in four districts in two states of India that have high under-five mortality rates
- Standard World Health Organization definition was used for identifying hospitalized cases of clinical pneumonia
- Radiological abnormalities interpreted by a panel of three trained radiologists at locations out of the surveillance network, blinded to each other as well as clinical features of the case
- Since pre-existing X-rays machines were used, there was a variation in the quality of images obtained, which were minimized by digitizing them centrally
- Since data of clinical examination was abstracted from hospital records, it could have resulted in inter-observer variation.

For peer review only - http://bmjopen.bmj.com/site/about/guidelines.xhtml

INTRODUCTION

Community-acquired pneumonia (CAP) is the single largest infectious cause of death in young children worldwide. Globally, pneumonia accounts for 16% of deaths in children under-five years of age and results in almost one million deaths (0.9 million children in 2016) every year[1 2]. Most deaths due to pneumonia occur in low and middle income countries particularly in sub-Saharan Africa and South Asia[2 3]. In India, there were approximately 0·44 million under-five deaths due to CAP in the year 2015 [4].

CAP could have viral or bacterial etiology[5 - 7]. Etiology varies from country to country and also across different time periods. Pediatric bacterial pneumonia is predominantly caused by *Streptococcus pneumoniae* and *Hemophilus influenzae Type B* while Respiratory syncytial virus and Influenza A or B virus are important contributors of pediatric viral pneumonia[5 6]. The World Health Organization (WHO) recommends the introduction of Pneumococcal Conjugate Vaccine (PCV) in the national immunization programme of countries with high child mortality rates, which includes India [8]. Consequently, PCV-13 was launched in May 2017 under the national immunization programme of five Indian states (Uttar Pradesh, Bihar, Rajasthan, Madhya Pradesh and Himachal Pradesh) in a phased manner[9]. It is expected to be rolled out in other parts of the country in the near future. Vaccination against *Hemophilus influenzae Type B* is already under the national immunization programme since 2011.

Differentiating bacterial from viral etiology of CAP on clinical features or by investigations remains difficult [7 10 11]. Several PCV probe trials have used radiographically confirmed end-point pneumonia, to be an outcome for vaccine efficacy and this has been endorsed by WHO[12-14].

For peer review only - http://bmjopen.bmj.com/site/about/guidelines.xhtml

The current study was a hospital-based surveillance to assess the radiological abnormalities in chest X-rays (CXRs) and to identify the demographic and clinical correlates of specific radiological abnormalities in children aged 2-59 months, hospitalized with WHO defined CAP, residing in pre-specified districts of Uttar Pradesh and Bihar, India.

METHODS

Study design and Setting

This prospective multi-site observational study was conducted in Lucknow and Etawah districts of Uttar Pradesh and Patna & Darbhanga districts of Bihar, India. Uttar Pradesh is the first most populated and Bihar third most populated state of India[15 16]. In Lucknow district 66.2% population resides in urban areas and in Patna district 43.07% [15 16]. In contrast, only 22.3% population of Etawah district and 9.74% population of Darbhanga district resides in urban areas [15 16]. All four project districts have alarmingly high infant and child mortality indicators [15-17]. The under-five mortality rates of Lucknow (58/1000), Etawah (85/1000), Patna (46/1000) and Darbhanga (77/1000) districts are above the national average (50/1000) [15-17]. Similarly, the infant mortality rates of Lucknow (44/1000), Etawah (56/1000), Patna (31/1000) and Darbhanga (44/1000) districts are also higher than the national average (41/1000) [15-17].

Study Population

A prospective, active, hospital-based surveillance system was established for this study [17 18]. Included in the surveillance were 117 public and private hospitals of four study districts which

For peer review only

provided either secondary or tertiary level care to admitted children. Surveillance officers visited the hospital every 48-72 hours to screen and recruit eligible cases. In between the visits they telephonically contacted the hospitals and made additional visits, if required. All children (2-59 months), hospitalized in network hospitals between January 2015 to April 2017, with history of fast breathing with/without chest in-drawing were screened [18].

Included were children hospitalized with symptoms of WHO defined CAP and residing in the project district [18]. WHO has developed guidelines for hospital-based management of common childhood illness such as pneumonia [19]. According to these guidelines, fast breathing $\geq$ 50 breaths/minute in a child aged 2–11 months and $\geq$ 40 breaths/minute in a child aged 12-59 months along with chest indrawing was categorized as having `pneumonia`[19]. A child presenting with cough or difficulty in breathing plus atleast one of the following: (a) oxygen saturation $<$ 90% or central cyanosis or (b) severe respiratory distress (e.g. grunting, very severe chest indrawing) or (c) signs of pneumonia with a general danger sign (inability to breastfeed or drink, lethargy or reduced level of consciousness, convulsions) was categorized as having `severe pneumonia`[19]. Excluded were children with cough for $\geq$ 14 days or those that had been hospitalized in last 14 days [18].

For peer review only

Sample Size

We assumed that the incidence of radiological pneumonia is 3/100 child years of observations. Then for a margin of error of 1.5/100 child years of observation, incidence of pneumonia in the community of 20/100 child years of observation, alpha level of 0.05, and power of 90% when the

For peer review only - http://bmjopen.bmj.com/site/about/guidelines.xhtml

estimated population of children under-five years of age in Lucknow district [20] is 750,000; 693 cases had to be included per district.

Data collection

Data was collected by surveillance officers who had postgraduate degree in social sciences and atleast 10 years experience in community based health research. After recruitment, they were imparted six-day centralized training on project procedures and logistics. Class-room as well as practical skills-training was given by the coordinating centre in Lucknow. Pre and post tests were conducted to ascertain knowledge and skills acquired by them through the training to ensure quality in data collection. The coordinating centre provided annual refresher trainings to the surveillance officers from all four sites in Lucknow.

After obtaining written, informed consent of the caregivers, data was collected through face-to-face interviews with them as well as by abstraction from hospital records. Socio-demographic data, obtained by interviewing caregivers, was: child`s age, gender, residence, birth order, immunization status, current breastfeeding status, parental education and occupation, smoking status of parents, family type, housing infrastructure, use of biomass fuel etc. Caregivers were also asked about the symptoms of disease and its duration in days.

Clinical data, recorded by pre-existing hospital staff at the time of hospitalization, was abstracted. Data was collected on anthropometry (weight and height), fever (axillary temperature ≥37.5°C), oxygen saturation by pulse oxymetry, pallor, central cyanosis, signs of pneumonia with a general danger sign and vital signs (heart rate and respiratory rate). Presence of wheezing

For peer review only - http://bmjopen.bmj.com/site/about/guidelines.xhtml

on auscultation of chest was abstracted or inquired from the treating physician. At the hospitals, clinicians generally used Integrated Management of Childhood Illness (IMCI) definitions[21] to identify pallor, cyanosis, wheeze on auscultation and general danger sign as it is incorporated in their medical undergraduate training. Most doctors of public health sector also receive a formal in-service training on IMCI [21]. Clinical outcome (survival or mortality) was noted from hospital records on follow up.[17 18].

Chest x-ray (CXR) image acquisition and archiving

CXR (poster-anterior view) was done when advised by the treating physician. These CXRs were obtained by the surveillance staff at the time of recruitment. CXRs were either analog or digital. In case of digital CXRs, second copy was obtained where possible. If only single analog image was available, then the hardcopy of CXR was obtained from the caregiver after the child was discharged. If the caregiver was not ready to give the hardcopy of CXR (in <1% cases), image of the same was captured by surveillance officers using 16 megapixel cell phone camera and portable CXR view-box.

CXRs of recruited cases were subsequently scanned and converted into digital format using a diagnostic-quality film image digitalizer (Microteck International Limited, model Medi 6000 plus) [22]. CXRs obtained/converted into digital image were stored as per the standard operating procedure and were subsequently archived for web-based radiological interpretation. Digital images were stored in JPEG format at 300 dpi resolution. Each CXR file was anonymized and given a unique identification number. Digital CXRs were uploaded on online data management software, developed especially for the project.

For peer review only - http://bmjopen.bmj.com/site/about/guidelines.xhtml

Interpretation of radiological images

A panel of radiologists was constituted for standardized interpretations of CXRs. Four radiologists were part of this panel, one of whom was Project co-investigator-Radiology (NK). All radiologists are faculty in medical teaching institutes and also look after pediatric radiology. They all have more than fifteen years experience in interpreting pediatric CXRs.

Radiologists were trained according to the methodology developed by Department of Immunization, Vaccines, and Biologicals of the WHO for research purpose [11]. An international WHO-certified trainer from the International Centre for Diarrhoeal Disease Research, Bangladesh imparted a two-day in-house training to the radiologists. Training objective was to standardize interpretation and coding of CXRs, to develop a CXR reporting form **[S1 Appendix]** and to provide training on web-based CXR retrieval and reporting system. During the training, 210 CXRs of WHO data set were used. For assessing concordance post training, another set of 48 CXRs was provided for interpretation to individual radiologists. Post-test agreement with WHO findings was calculated, which was about 80%. Inter-observer variation was about 25% and was for minor interpretation like quality of film, end point infiltrates etc. Repeat training was conducted on an additional set of 44 CXRs provided by WHO to ensure standardization in interpretation. Thereafter, concordance achieved by the radiologists was reviewed quarterly by the study arbitrator. Radiologists met annually to review key concepts and discuss challenges faced in interpreting CXRs.

For peer review only - http://bmjopen.bmj.com/site/about/guidelines.xhtml

For peer review only

After training, radiologists independently reviewed CXRs and registered their findings in an online standardized chest radiograph interpretation form **[S1 Appendix].** For optimal viewing of CXRs, all radiologists used similar workstations. Specifications were provided for the computer monitor and hardware to be used. It was ensured that monitors had the correct brightness and contrast adjustment for optimal viewing.

During online evaluation, radiologists reported the quality of film as `*interpretable*` or `*un-interpretable*`. Radiologists categorized `*interpretable*` CXRs as either `*adequate/optimal*` for features that allowed confident interpretation of consolidation and pleural effusion as well as other infiltrates or `*suboptimal*` for features that allowed interpretation of consolidation and pleural effusion, but not of other infiltrates or findings. In `*un-interpretable*` CXRs, no comment was possible for radiological abnormality with respect to presence or absence of consolidation or pleural effusion or other infiltrates [12].

After interpreting film quality, radiologists evaluated interpretable CXRs for abnormal radiological findings. For each CXR evaluated, radiological abnormality could be presence of consolidation, other infiltrates or pleural effusion. `*Consolidation*` was defined as a dense or confluent opacity that occupies a portion or whole of a lobe or the entire lung, that may or may not contain air bronchograms. `*Other infiltrates*` were defined as linear and patchy opacities (interstitial infiltrate) in a lacy pattern, featuring peri-bronchial thickening and multiple areas of atelectasis; also including minor patchy infiltrates that are not of sufficient magnitude to constitute endpoint consolidation, and small areas of atelectasis that in children may be difficult to distinguish from consolidation. `*Pleural effusion*` was defined as the fluid in the lateral

For peer review only - http://bmjopen.bmj.com/site/about/guidelines.xhtml

pleural space between the lung and chest wall that is spatially associated with a pulmonary parenchymal infiltrate (including `other infiltrates`) or has obliterated enough of the hemithorax to obscure any infiltrates. In most cases, this will be seen at the costo-phrenic angle or as a layer of fluid adjacent to the lateral chest-wall and this does not include fluid seen in the horizontal or oblique fissures [12]. Primary end point pneumonia (PEP) for research purpose was the presence of consolidation or pleural effusion which could be with or without other infiltrates.

Final conclusions were categorised as: (a) "*Abnormal*" when it was `*PEP only*` or `*Other infiltrates only*` or `*Both PEP and other infiltrates*` and (b) `*Normal*` when no findings were abnormal[12].

After radiological interpretation, online data was archived, stored and checked for inconsistencies and completeness by the data manager. CXRs with concordant and discordant interpretations were identified. Interpretations were considered concordant when there was an agreement between two or more radiologists on final conclusions and discordant if all the three radiologists disagreed. Discordant interpretations were forwarded to the study arbitrator (NK). Arbitrator assessed discordant CXRs online and her interpretation was taken as final.

Data management and statistical analysis

Clinical data of hospital surveillance network was entered online in customized software. Primary entry was done by the four participating sites. Secondary data entry was done by the coordinating site in separate customized software. Anonymized CXRs were uploaded on customized software. Each of the three panelists assessed the CXRs online, blind to peer

For peer review only - http://bmjopen.bmj.com/site/about/guidelines.xhtml

assessments as well as clinical features of the case. CXR assessment data was downloaded from the online software in MS Access database.

Exploratory data analysis was performed for detection of outlier and missing observations for all the variables. Descriptive statistics was calculated for continuous variables as mean ± standard deviation and categorical variables in percent. Un-interpretable CXRs were not analyzed. Among interpretable CXRs, those radiological abnormalities where there was concordance between the two radiologists, were taken as final. Weight-for-age (WAZ) z-score each child was calculated using WHO Anthro Survey Analyser [23]. Weight of 7.59% (215/2829) children was missing in our data. Missing weight of recruited children was estimated using regression based imputation technique [24]. Kappa statistics was performed for agreement analysis among radiologists for CXRs findings. Statistical analysis was performed using SPSS version 22.0 (Chicago, IL) [25]. A p-value of <0·05 was taken as statistically significant using a two-tailed distribution.

Univariate analysis was performed to evaluate heterogeneity, stratified by four participating sites for socio-demographic variables such as child`s age, gender, residence, birth order, immunization status, current breastfeeding status, parental education and occupation, smoking status of parents, family type, housing infrastructure, use of biomass fuel and for clinical variables such as weight, height, duration of fever and oxygen saturation.

We report proportions of radiological abnormalities among hospitalized children for CAP by four districts. Univariate analysis was performed to assess association of socio-demographic variables and clinical signs of CAP with radiological abnormalities. Chi-square test was used for

For peer review only - http://bmjopen.bmj.com/site/about/guidelines.xhtml

categorical variables and student's t-test for continuous variables. ANOVA test was used for more than two groups to test the significance of continuous variables. Multivariate unconditional logistic regression was performed find association of presence of various radiological abnormalities, controlling for district of residence and other variables that had univariate association with radiological abnormalities (p value $\leq$0.2) and/or were clinically meaningful.

We developed four models and in these four models dependent (outcome) were:

Model I: Abnormal vs. Normal

Model II: Primary End Point Pneumonia alone or with other infiltrates vs. Normal

Model III: Primary End Point Pneumonia alone vs. Normal

Model IV**:** Other infiltrates only vs. Normal

Independent variables that were kept across all the four models were : participating districts, age, gender, use of biomass fuel, symptoms of CAP such as duration of illness, presence of wheeze on ascultation, pallor, vomiting everything and malnutrition status of the case [WAZ$\leq$ -2 SD (malnourished) and WAZ $\leq$ -3 SD (severely malnourished)].

Patient and public involvement in research

Patients or public were not involved in the development of research question, study design or conducting the research. Reporting of this research conforms to the guidelines for Strengthening the Reporting of Observational Studies in Epidemiology (STROBE) [26]**.**

RESULTS

For peer review only - http://bmjopen.bmj.com/site/about/guidelines.xhtml

For peer review only

A total of 3290 hospitalized cases were screened in hospital surveillance network of Lucknow and Etawah districts of Uttar Pradesh and Patna & Darbhanga districts of Bihar. Out of these, 3214 cases fulfilling the WHO diagnosis of CAP were included **[Figure 1].** Among them, 3195 (99.40%) cases were enrolled with CXRs and only in 19 (1.0%) cases CXRs were not done. Out of these 88.54% (2829/3195) CXRs were found interpretable and remaining 11.45% (366/3195) were found un-interpretable by radiologists. Among interpretable CXRs, 99.11 % (2804/2829) children had `severe pneumonia` as per the WHO criteria [19].

Among interpretable CXRs, we found 22.44% (635/2829) children had PEP alone or with infiltrates, 12.09% (342/2829) had other infiltrates only and 65.46% (1852/2829) had normal CXRs findings [**Figure 1**]. Concordance among ≥ 2 radiologists on final conclusion of CXRs findings was 86.0%. Kappa statistics was calculated for agreement of CXRs findings between Reader 1 versus Reader 2 (K_1=0.31), Reader 2 versus Reader 3 (K_2=0.46) and Reader 3 versus Reader 1(K_3=0.42).

Table 1 shows univariate distribution of socio-demographic and clinical variables among hospitalized cases across four participating districts. A statistically significant variation was observed in all socio-demographic variables such as place of residence, type of house, type of family, maternal and paternal education and occupation, use of biomass fuel and parental smoking status across the four districts. We also report other clinical variables of recruited cases across the four districts in **table 1**. Oxygen saturation by pulse-oxymetry was statistically significantly different across the sites, the proportion of cases with oxygen saturation < 90 % was found also found statistically significant in children across four districts (p < 0.0001).

For peer review only - http://bmjopen.bmj.com/site/about/guidelines.xhtml
For peer review only

Table 1: Distribution of socio-demographic and clinical variables among hospitalized children for participating districts (Jan 2015-April 2017)

Characteristics	Lucknow	Etawah	Patna	Darbhanga	Total
**Socio-demographic Characteristics**	**n=1025 (%)**	**n=389 (%)**	**n=744 (%)**	**n=671 (%)**	**N=2829 (%)**
**Gender**					
Male	659(64.29)	287(73.78)	557(74.87)	502(74.81)	2005(70.87)
**Place of residence**					
Rural	195(19.02)	279(71.72)	304(40.86)	614(91.51)	1392(49.20)
Urban	830(80.98)	110(28.28)	440(59.14)	57(8.49)	1437(50.80)
**Family Type**					
Joint	688(67.12)	360(92.54)	707(95.03)	383(57.08)	2138(75.57)
Nuclear	337(32.88)	29(7.46)	37(4.97)	287(42.77)	690(24.39)
**House type**					
Mud	64(6.24)	54(13.88)	123(16.53)	374(55.74)	615(21.74)
Bricks	854(83.32)	256(65.81)	453(60.89)	85(12.67)	1648(58.25)
Combined	107(10.4)	79(20.31)	168(22.58)	212(31.59)	566(20.01)
**Mother's Education**					
No formal education	203(19.80)	56(14.40)	328(44.09)	496(73.92)	1083(38.28)
Class I-V	108(10.54)	28(7.20)	82(11.02)	38(5.66)	256(9.05)
Class VI-XII	379(36.98)	176(45.24)	243(33.66)	112(16.69)	910(32.17)
Graduate/ Post graduation	335(32.68)	129(33.16)	91(12.23)	25(3.73)	580(20.50)
**Father's Education**					
No formal education	167(16.29)	29(7.46)	153(20.56)	345(51.42)	694(24.53)
Class I-V	85(8.29)	19(4.88)	91(12.23)	82(12.22)	277(9.79)
Class VI-XII	437(42.63)	206(52.96)	328(44.09)	205(30.55)	1176(41.57)
Graduate/ Post graduation	336(32.78)	135(34.70)	172(23.12)	39(5.81)	682(24.11)
**Birth Order**					
1st	435(42.44)	187(48.07)	315(42.34)	192(28.61)	1129(39.91)
2nd	343(33.46)	120(30.85)	235(31.59)	258(38.45)	956(33.79)
3rd	153(14.93)	47(12.08)	129(17.34)	137(20.42)	466(16.47)
More than 3rd	93(9.07)	35(9.00)	62(8.33)	83(12.37)	273(9.65)
**Immunization Status**					
Complete for age	792(77.27)	300(77.12)	711(95.56)	544(81.07)	2347(82.96)
Incomplete for age	220(21.46)	84(21.59)	25(3.36)	126(18.78)	455(16.08)
Unimmunized	13(1.27)	5(1.29)	8(1.08)	1(0.15)	27(0.95)
**Currently Breast Feeding**					
Yes	653(63.71)	256(65.81)	589(79.17)	537(80.03)	2035(71.93)
No	372(36.29)	133(34.19)	155(20.83)	134(19.97)	794(28.07)
**Father's Occupation**					
Unemployed	13(1.27)	20(5.14)	27(3.63)	63(9.39)	123(4.35)
Daily wages	329(32.10)	81(20.82)	165(22.18)	474(70.64)	1049(37.08)
Salaried/ Professional	397(38.73)	104(26.74)	245(32.93)	55(8.20)	801(28.31)

For peer review only

For peer review only - http://bmjopen.bmj.com/site/about/guidelines.xhtml

Self-Employment	286(27.90)	184(47.30)	307(41.26)	79(11.77)	856(30.26)
**Mother's Occupation**					
Home maker	961(93.76)	376(96.66)	701(94.22)	484(72.13)	2522(89.15)
Daily wages	17(1.66)	3(0.77)	17(2.28)	171(25.48)	208(7.35)
Salaried/Professionals	47(4.59)	9(2.31)	18(2.42)	7(1.04)	81(2.86)
Self-Employment	0(0.0)	1(0.26)	8(1.08)	9(1.34)	18(0.64)
**Biomass fuel**					
Yes	211(20.59)	245(62.98)	263(35.35)	609(90.76)	1328(46.94)
No	814(79.41)	144(37.02)	481(64.65)	62(9.24)	1501(53.06)
**Smoking Status-Father**					
Yes	152(14.83)	45(11.57)	56(7.53)	59(8.79)	312(11.03)
No	873(85.17)	344(88.43)	688(92.47)	612(91.21)	2517(88.97)
**Indoor smoking-Father**					
Yes	83(8.10)	21(5.40)	16(2.15)	43(6.41)	163(5.76)
No	942(91.90)	368(91.60)	728(97.85)	628(93.59)	2666(94.24)
**Smoking Status-Family member**					
Yes	129(12.59)	55(14.14)	45(6.05)	102(15.20)	331(11.70)
No	896(87.41)	334(85.86)	699(93.95)	569(84.80)	2498(83.30)
**Indoor smoking – Family member**					
Yes	84(8.20)	27(6.94)	27(3.63)	94(14.01)	232(8.20)
No	941(91.80)	362(93.06)	717(96.37)	577(85.99)	2597(91.80)
**Clinical Variables at the time of admission at hospital**	**n Mean± SD**	**n Mean± SD**	**n Mean± SD**	**n Mean± SD**	**n Mean± SD**
Age (in months)	1025 14.53±13.88	389 10.69±10.95	744 10.26±11.35	671 12.30±13.29	2829 12.35±12.85
Height (in cm)	303 68.61±13.78	324 70.66±13.75	34 64.38±10.25	266 70.46±12.14	927 69.70±13.26
Weight (in kg)	1025 7.96±2.97	389 7.34±2.73	744 7.11±2.78	671 7.78±2.93	2829 7.61±2.90
Fever Duration (in days)	929 4.46±2.71	321 3.59±2.37	689 4.25±2.52	569 3.54±2.47	2508 4.08±2.59
**Respiratory Rate**					
Respiratory Rate [2-11 months]	602 53.38±14.05	272 60.87±9.60	540 53.82±10.16	451 60.78±7.26	1864 56.37±11.49
Respiratory Rate [12-59 months]	423 47.75±14.17	117 53.22±13.17	204 45.59±10.11	220 58.03±6.83	964 50.30±12.76
	**n (%)**	**n (%)**	**n (%)**	**n (%)**	**n (%)**
Oxygen saturation done	528 (51.51)	343 (88.17)	236 (34.25)	319 (56.06)	1426 (50.40)
Oxygen saturation < 90%	61 (11.53)	57 (16.61)	49 (20.76)	43 (13.47)	210 (14.72)

For peer review only - http://bmjopen.bmj.com/site/about/guidelines.xhtml

Grunting	461 (44.98)	353 (90.75)	687 (92.34)	649 (96.72)	2150 (76.00)
Very severe chest in-drawing	953 (92.97)	352 (90.49)	739 (99.33)	651 (97.02)	2695 (95.26)
**Signs of Pneumonia with a general danger sign**					
Lethargy or reduced level of consciousness	423 (41.27)	259 (66.58)	6 (0.81)	412 (61.40)	1100 (38.88)
Inability to breastfeed or drink	291 (28.39)	259 (66.58)	75 (10.08)	312 (46.50)	937 (33.12)
Convulsions	16 (1.56)	19 (4.58)	13 (1.75)	100 (14.90)	148 (5.23)
Central cyanosis	15 (1.46)	7 (1.80)	26 (3.49)	14 (2.09)	62 (2.19)

Table 2 shows proportions of radiological pneumonia which includes PEP alone or with other infiltrate and other infiltrates among cases hospitalized for CAP in four participating districts. We observed statistically significant district-wise heterogeneity in radiological abnormalities. We found higher proportion of radiological abnormalities in Patna and Lucknow districts, which have a larger urban population, and lower proportion in Etawah and Darbhanga districts, which in contrast have a larger rural population. We also observed correspondingly higher proportion of PEP alone or with other infiltrates in districts of Lucknow and Patna and lower in districts of Etawah and Darbhanga.

Table 2 also describes univariate distribution of socio-demographic and clinical factors of CAP among hospitalized children (2-59 months). Statistically significantly higher proportion of females hospitalized for CAP had radiologically abnormal CXR. Likewise, statistically significantly higher proportion of abnormal versus normal CXRs findings were reported in

For peer review only - http://bmjopen.bmj.com/site/about/guidelines.xhtml

hospitalized cases who had symptoms of fever, pallor, wheezing on auscultation, vomiting everything or were malnourished.

Table 2: Distribution of socio-demographic and clinical factors by chest radiograph findings among hospitalized children from January 2015-April 2017

	Interpretable chest X rays				Abnormal chest X rays		
	**N=2829**	**Normal 1852 n (%)**	**Abnormal 977 n (%)**	**p value**	**PEP* alone or with other infiltrate 635 n (%)**	**Other infiltrates 342 n (%)**	**p value**
**Participating site** (row %)							
Lucknow	1025	636 (62.05)	389 (37.95)	<0.0001	282 (72.49)	107 (27.51)	<0.0001
Etawah	389	275 (70.69)	114 (29.31)		73 (64.04)	41 (35.96)	
Patna	744	457 (61.42)	287 (38.58)		184 (64.11)	103 (35.89)	
Darbhanga	671	484 (72.13)	187 (27.87)		96 (51.34)	91 (48.66)	
**Socio-demographic & clinical factors** (column %)							
**Age-group (months)**							
2-11	1865	1223 (66.04)	642 (65.71)	0.86	409 (64.41)	233 (68.13)	0.26
12-59	964	629 (33.96)	335 (34.29)		226 (35.59)	109 (31.87)	
**Gender**							
Male	2005	1354 (73.11)	651 (66.63)	<0.0001	426 (67.09)	225 (65.79)	0.72
Female	824	498 (26.89)	326 (33.37)		209 (32.91)	117 (34.21)	
**Place of residence**							
Rural	1392	921 (49.73)	471 (48.21)	0.44	299 (47.09)	172 (50.29)	0.34
Urban	1437	931 (50.27)	506 (51.79)		336 (52.91)	170 (49.71)	

For peer review only - http://bmjopen.bmj.com/site/about/guidelines.xhtml

**Biomass fuel**							
Yes	1501	867 (46.81)	461 (47.19)	0.44	294 (42.30)	167 (48.83)	0.24
No	1328	985 (53.19)	516 (52.81)		341 (53.70)	175 (51.17)	
**Immunization status**							
Complete for age	2347	1546 (83.48)	801 (81.99)	0.32	516 (81.26)	285 (83.33)	0.54
Incomplete	482	306 (16.52)	176 (18.01)		119 (18.74)	57 (16.67)	
**Clinical Features**							
Fever	2499	1616 (87.26)	883 (90.38)	0.014	575 (90.55)	308 (90.06)	0.82
Pallor	764	465 (25.11)	299 (30.60)	0.002	200 (31.50)	99 (28.95)	0.41
Wheeze on auscultation	2054	1377 (74.35)	677 (69.29)	0.005	415 (65.35)	262 (76.61)	0.0003
Duration of illness fever [days] (n, Mean ± SD)	2499	1611 3.91±2.51	888, 4.40±2.70	<0.0001	577, 4.57±2.82	342, 4.08±2.44	0.011
**Respiratory Rate**							
Respiratory Rate [2-11 months] (n, Mean ± SD)	1865	1243 55.52±11.29	642 57.99±11.70	<0.0001	409 58.12±11.88	233 57.74±11.40	0.69
Respiratory Rate [12-59 months] (n, Mean ± SD)	964	629 49.78±12.41	335 51.28±13.37	0.08	226 51.35±13.31	109 51.12±13.35	0.88
Fast Breathing for age [2-11 months]	1735	1130 (61.02)	605 (61.92)	0.11	384 (60.47)	221 (64.62)	0.69
Fast Breathing for age [12-59 months]	862	562 (30.35)	300 (30.71)	0.92	204 (32.13)	96 (28.07)	0.53
**Signs of Pneumonia with a general danger sign n (%)**							
Lethargy or reduced level of consciousness	1101	732 (39.52)	369 (37.77)	0.39	247 (38.90)	122 (35.67)	0.33
Inability to breastfeed or drink	937	612 (33.05)	325 (33.27)	0.46	211 (33.23)	114 (33.33)	0.97
Convulsions	148	98 (5.29)	50 (5.12)	0.93	33 (5.20)	17 (4.97)	0.87
Central Cyanosis	62	39	23	0.34	16	7	0.64

For peer review only - http://bmjopen.bmj.com/site/about/guidelines.xhtml

		(2.11)	(2.35)		(2.52)	(2.05)	
**Malnutrition Status**							
Normal *	1880	1293 (69.82)	587 (60.08)		367 (57.80)	220 (64.33)	
Malnutrition*	517	333 (17.98)	184 (18.83)	< 0.0001	122 (19.21)	62 (18.13)	0.06
Severe malnutrition*	432	226 (12.20)	206 (21.08)		146 (22.99)	60 (17.54)	

*Normal-weight of age z score > -2SD; Malnutrition-weight-for-age z ≤ -2SD and Severe malnutrition-weight-for- age z ≤ -3SD

Table 3 describes four multivariate unconditional logistic regression models to find associates of abnormal CXR findings. After controlling for age, gender, symptoms of pneumonia, duration of illness, biomass fuel and malnutrition status of cases, statistically significant district-wise heterogeneity remained in the first three models. Models I, II and III had similar associates for radiological abnormalities whereas Model IV was different. Across all the four models, female gender and those with severe malnutrition had statistically significantly higher risk for having abnormal CXRs. A higher risk of radiological abnormalities was also observed in those children with longer duration of illness.

Table 3: Independent Associations between Chest Radiograph Findings and demographic and clinical factors, among hospitalized children January 2015-April 2017

**Variables**	**Model – I Abnormal/Normal [Ref]**		**Model – II PEP alone or with other infiltrate /Normal [Ref]**		**Model – III PEP alone / Normal [Ref]**		**Model – IV Other infiltrate / Normal [Ref]**	
	**Adjusted Odd Ratio (95%CI)**	**p value**	**Adjusted Odd Ratio (95%CI)**	**p value**	**Adjusted Odd Ratio (95%CI)**	**p value**	**Adjusted Odd Ratio (95%CI)**	**p value**
**Districts**								
Lucknow vs. Others	1.58 (1.20-2.10)	<0.0001	2.07 (1.48-2.89)	<0.0001	2.20 (1.52-3.19)	<0.0001	0.98 (0.65-1.47)	0.93
Etawah vs. Others	1.22 (0.88-1.70)	0.23	1.30 (0.87-1.95)	0.19	1.49 (0.95-2.30)	0.07	1.17 (0.74-1.87)	0.50
Patna vs. Others	1.67 (1.27-2.20)	<0.0001	1.89 (1.36-2.64)	<0.0001	2.25 (1.56-3.24)	<0.0001	1.39 (0.95-2.07)	0.09

For peer review only - http://bmjopen.bmj.com/site/about/guidelines.xhtml

**Age – Group (months)**								
2-11 [Ref]								
12-59	0.92 (0.77-1.10)	0.34	0.95 (0.77-1.17)	0.62	1.03 (0.82-1.29)	0.79	0.86 (0.66-1.13)	0.27
**Gender**								
Male [Ref]								
Female	1.39 (1.16-1.66)	<0.0001	1.34 (1.08-1.65)	0.008	1.28 (1.01-1.61)	0.03	1.48 (1.14-1.92)	0.004
**Symptoms of pneumonia¶**								
Wheezing	0.83 (0.68-1.01)	0.06	0.72 (0.57-0.90)	0.005	0.75 (0.59-0.96)	0.02	1.14 (0.83-1.55)	0.42
Pallor	1.30 (1.08-1.58)	0.006	1.28 (1.03-1.60)	0.02	1.22 (0.95-1.55)	0.12	1.34 (1.01-1.77)	0.04
Vomiting everything	0.90 (0.75-1.09)	0.28	0.80 (0.64-0.99)	0.04	0.78 (0.62-1.01)	0.05	1.09 (0.83-1.08)	0.51
**Duration of illness, fever (days)**	1.06 (1.04-1.09)	<0.0001	1.08 (1.04-1.12)	<0.0001	1.08 (1.04-1.12)	<0.0001	1.03 (0.98-1.48)	0.24
**Biomass fuel**	1.28 (1.05-1.57)	0.02	1.39 (1.10-1.76)	0.006	1.40 (1.14-1.88)	0.003	1.08 (0.79-1.45)	0.64
**Malnutrition Status**								
Normal *[Ref]								
Malnutrition*	1.18 (0.93-1.45)	0.15	1.17 (0.91-1.52)	0.23	1.17 (0.88-1.55)	0.27	1.12 (0.82-1.52)	0.47
Severe malnutrition*	1.65 (1.31-2.09)	<0.0001	1.82 (1.34-2.36)	<0.0001	1.87 (1.41-2.47)	<0.0001	1.62 (1.71-2.23)	0.003

Abbreviations used: [Ref] Reference Category

Footnotes: ¶ No signs of pneumonia taken as a reference
*Normal: weight-for-age z score> -2SD; Malnutrition: weight-for -age z ≤ -2SD; Severe malnutrition: weight-for-age z ≤ -3SD

DISCUSSION

This active, prospective hospital-based surveillance study was conducted to assess the radiological abnormalities in children (2-59 months) residing in pre-specified districts of Uttar Pradesh and Bihar, India and hospitalized with CAP. The study was conducted from January 2015 to April 2017, prior to introduction of PCV in the national immunization programme of the Government of India[9].

For peer review only - http://bmjopen.bmj.com/site/about/guidelines.xhtml

For peer review only

In our study, among interpretable CXRs, we found that 22.44% (635/2829) children had PEP alone or with infiltrates, 12.09% (342/2829) had other infiltrates only and 65.46% (1852/2829) had normal CXRs findings. Our study used WHO case definition for CAP [19]. A panel of three trained radiologists interpreted CXRs, adopting WHO recommended methodology [11 12]. These make our study methodology robust and results generalizable.

There were 88.54% (2829/3195) interpretable CXRs in the current study. This is similar to 83% (3587/3973) interpretable CXRs reported by Pneumonia Etiology Research for Child Health (PERCH) study conducted on 4232 children (1-59 months) to assess the etiology of CAP in nine sites of seven countries [27]. Consistent with PERCH findings, a vaccine probe trial conducted in Gambia found the proportion of interpretable CXRs among unvaccinated cases of pneumonia to be 84.32% (242/287)[28].

There have been several studies in the past two decades, which have reported CXRs findings in hospitalized cases of CAP. Almost all of these were conducted before the introduction of PCV in their respective regions. A small prospective study conducted in Ethiopia reported radiological abnormality in CXRs in 48.3% (95% CI 39.49-57.22) among 122 children (3 months-14 years) clinically diagnosed with WHO-defined severe pneumonia[29]. Similar findings were reported from the Gambian vaccine probe trial where the proportion of radiological abnormality was 45% (95% CI: 43.35-46.46) among unvaccinated hospitalized cases of clinical pneumonia[28]. PERCH study found that 54% (95% CI: 52.31-55.57) of CXRs among cases of CAP were abnormal [27]. In all of these studies, proportion of cases with abnormal CXRs is higher than 34.5% (95% CI 32.8-36.3) found by us in the current study. However, our findings are similar to PERCH rural

For peer review only - http://bmjopen.bmj.com/site/about/guidelines.xhtml

study site of Matlab, Bangladesh that reported radiological abnormality in 35.3% (95% CI: 29.77-40.85) CXRs of hospitalized cases of CAP [27]. Another PERCH urban site of Dhaka, Bangladesh reported 63.10% (95% CI 56.18 -70.02) cases with abnormal CXRs [27]. In our study, radiological abnormalities in CXRs were higher in cases from largely urban districts of Patna and Lucknow compared to rural districts of Darbhanga and Etawah. This is consistent with rural-urban differences in Bangladesh sites of PERCH. Variation in CXR findings among cases of CAP may be due to infecting organism, immune response of patient and prior duration of disease.

In 2016, WHO's Department of Immunization, Vaccines and Biologicals standardized the categorization of radiological pneumonia and established that PEP can be taken as a good surrogate marker of bacterial pneumonia in epidemiological and vaccine efficacy studies[12]. In our study, 22.44 % (95% C.I. 20.90 -23.98), CXRs were having PEP alone or with other infiltrates. This is similar to PERCH study that reported PEP alone or with other infiltrates in 27% (95% C.I. 25.50 -28.40) hospitalized cases of CAP[27]. Another study conducted in Gambia reported that 45% (95% CI: 43.35-46.46) non-vaccinated children had PEP and/or other infiltrates [28]. PEP has been associated with increased risk of treatment failure (p=0.002), increased length of hospitalization (p=0.0003) and more days of respiratory support (p=0.002) in Botswana when compared with cases reporting` no significant pathology` on CXRs[30].

In our study, female gender (p<0.001) was at the higher risk of developing radiological abnormalities compared to males (**table 3**). The results are in concordance with a hospital-based case-control study carried out in Brazil that reported male gender as a protective factor against

For peer review only - http://bmjopen.bmj.com/site/about/guidelines.xhtml

pneumonia (OR = 0.53; 95 % CI 0.39–0.72) [31]. Another study in Mozambique, Africa reported that male gender was not significantly associated with presence of radiological abnormalities (OR =0.77; 95 % CI 0.56–1.05) in children (0-59 months) suffering from severe pneumonia [32]. However, in contrast, a Gambian study reported male preponderance for all pneumonia that was most marked for `other infiltrates/abnormalities` pneumonia[28].

In our study, it was observed that there was differential care-seeking by gender for CAP in all four project sites. Although females admitted with CAP were at higher risk of having radiological abnormalities, lower proportion were hospitalized for it. Gender inequality in health care seeking for females is common in India, as in other South Asian countries [33 34]. Since there is no health-care financing or health insurance provision in India, in case of severe illness, parents are less likely to incur out-of-pocket expenditure or incur debts to pay expenses on medical treatment of their daughters compared to sons [35]. Another Indian study found that male children were five times more likely to be taken early for medical care and three times more likely to be seen by qualified medical doctors compared to female children [36]. We also found that majority of hospitalized cases of pneumonia were from urban areas, in contrast with observations of other researchers who report poor health care seeking from rural areas[37].

A systematic review with meta-analysis conducted in 2019 suggests that no one clinical feature is sufficient on its own to diagnose radiological pneumonia [38]. However other socio-demographic and clinical correlates of abnormal CXRs found by us (Model 1), which increased the risk of radiological abnormalities, were presence of pallor, severe malnutrition, longer duration of illness and exposure to biomass fuel. Exposure to biomass fuel used for cooking is an

For peer review only

important factor that increases the risk of CAP in developing countries[39]. In rural India, majority of the households use biomass fuel like firewood, dung cakes and wood for cooking [40]. Young children are at risk to adverse effects of exposure to biomass fuel as either the households have no separate cooking space or have poor ventilation and sometimes young children stay with their mother while she cooks.

Other correlates of PEP/Radiological Pneumonia (Models II and III), besides those found in Model I, were presence of vomiting everything and wheeze on auscultation, both of which were found to be protective. These symptoms/signs are more often reported in viral pneumonia[41]. Correlates of radiological abnormalities of `other infiltrates` (Model IV), which increased the risk, were again female gender, pallor and severe malnutrition. Hence it is difficult to attribute radiological findings of other infiltrates to either bacterial or viral etiology.

Based on our study, almost two-third hospitalized cases of CAP had normal CXRs and this could be perhaps of viral etiology. This is supported by a recent study that reported 61.4% (95% CI 57·3–65·6) cases to be viral[41]. Among one-third of cases of CAP had abnormal CXRs and thus were more likely to be bacterial in etiology.

In India, 13-valent PCV is given using a three dose schedule (2 primary and one booster) at 6 weeks, 14 weeks and 9 months of age. PCV 13 provides coverage against 13 serotypes (1, 3, 4, 5, 6A, 6B, 7F, 9V, 14, 18C, 19A, 19F and 23F) causing pneumococcal pneumonia[42]. Several studies have assessed serotype distribution of pneumococcal disease among children in India. A study conducted in Vellore, India found that the most common serotypes causing invasive

For peer review only - http://bmjopen.bmj.com/site/about/guidelines.xhtml

infections among under-five children were 14, 19F, 5, 6A and 6B, all of which were covered by the 13-valent PCV [43]. Another population-based surveillance study conducted in rural Bangladesh found that the most common serotypes were 1, 5, 14, 18C and 19A and 38 and these comprised more than three-fourth of the *S. pneumoniae* isolates [44]. A systematic review and meta-analysis of data collected on Invasive Pneumococcal Disease serotypes from under-five children during the pre-PCV period (between 1980–2007) found that serotypes included in both the 10-valent and 13-valent PCVs accounted for 10 million cases and 600,000 deaths worldwide[45].

Several strengths of the study are worth-noting. This was an active, prospective, multi-site study where recruitments were done from a large hospital surveillance network established especially for the study in four districts of two Indian states that have high under-five mortality rates. Standard WHO definition was used to identify hospitalized cases of CAP. Radiological abnormalities were interpreted by a panel of three trained radiologists at locations out of the surveillance network, blinded to each other as well as clinical features of the case. Despite these strengths, our study findings have certain limitations. First, in our study, pre-existing x-rays machines which were not of uniform specification, were used. This might have caused variation in quality of CXR images, though this error was minimized by digitizing the CXR images centrally. Secondly, in our study, clinical data collection was recorded by physicians in the network hospitals and this could be subject to observer bias. This could also have lead to possibly over reporting of presence of wheezing. In this study, we have not collected information on use of antibiotic prior to hospitalizations, as such information is not available reliably. However, in another study, done in one of the network hospitals of Lucknow in the recent past, it

For peer review only - http://bmjopen.bmj.com/site/about/guidelines.xhtml

was found that 70.5% children tested positive for antibiotics on urine examnation[46]. Prior use of antibiotics could have possibly lead to underestimation of radiological pneumonia. We also observed that pulse oxymetry was routinely done in the network hospitals. This could have an impact on the case management but would not have affected the radiological findings of CXRs.

CONCLUSION

Among hospitalized cases of community-acquired pneumonia, almost one-third children had abnormal chest radiographs of which about two-thirds had abnormalities related with possible bacterial etiology *(Streptococcus pneumonia).* Hence, the introduction of pneumococcal vaccination is likely to reduce the burden of childhood pneumonia in India. Since the study was done prior to the introduction of PCV in India, continued surveillance will be required to assess the impact of PCV on radiological findings in cases admitted with CAP. The impact of introduction of PCV in the national immunization programme on under-five mortality rate and burden of CAP needs to be assessed.

Acknowledgements: We thank the state governments of Uttar Pradesh and Bihar for giving us permission to conduct the study. We extend our gratitude to participating tertiary and secondary care hospitals for their contribution in the study. We offer thanks to Dr. Kamrun Nahar from the International Centre for Diarrhoeal Disease Research, Bangladesh for imparting training to the panel of study radiologists. Members of the Lucknow Community-Acquired Pneumonia (CAP) Group: King George's Medical University (KGMU), Lucknow, India: Prof. Shally Awasthi, Department of Pediatrics, Prof. Neera Kohli, Department of Radio-diagnosis,

For peer review only

For peer review only - http://bmjopen.bmj.com/site/about/guidelines.xhtml

Prof. Monika Agarwal, Department of Community Medicine; Mr. Puneet Dhasmana, Project Staff; Patna Medical College and Hospital, Patna, Bihar-Prof. Neelam Verma and Prof. Chandra Bhushan Kumar, Department of Pediatrics; Darbhanga Medical College and Hospital, Darbhanga, Bihar, India- Prof. Chittaranjan Roy, Department of Community Medicine, Prof. Kripanath Mishra, Department of Pediatrics; Uttar Pradesh University of Medical Sciences, Etawah, Uttar Pradesh, India- Prof. Pankaj Kumar Jain, Department of Community Medicine, Prof. Rajesh Yadav, Department of Pediatrics.

Contributors: The study was conceived and designed by SA. CAP study group performed data acquisition.CMP and NM[1]conducted the statistical analysis of the data. The paper was written by SA, TR, MA and CMP. AC, NM[3], RCS and NK interpreted chest x-rays. All authors were involved with drafting and revising the work and approved the final submission.

Funding statement: The study was supported by Bill & Melinda Gates Foundation (https://www.gatesfoundation.org/) via Grant No: OPP1118005**.** Funding agency had no role in the design of the study and collection, analysis and interpretation of data, or in writing the manuscript.

Competing interests: None declared.

Patient consent for publication: Not required.

Ethics approval: The Institutional Ethics Committee of King George's Medical University (Lucknow), The Uttar Pradesh University of Medical Sciences (Etawah), Patna Medical College and Hospital (Patna) and Darbhanga Medical College and Hospital (Darbhanga) gave ethical approval for the conduct of study.

Provenance and peer review: Not commissioned; externally peer reviewed.

For peer review only

For peer review only - http://bmjopen.bmj.com/site/about/guidelines.xhtml

Data sharing statement: The data contained within this study can be obtained by writing to shally07@gmail.com

Copyright information: This is an Open Access article distributed in accordance with the Creative Commons Attribution Non Commercial (CC BY-NC 4.0) license, which permits others to distribute, remix, adapt, build upon this work non-commercially, and license their derivative works on different terms, provided the original work is properly cited and the use is non-commercial. See: http://creativecommons.org/licenses/by-nc/4.0/

Supporting Information

S1 Appendix: Chest radiograph interpretation form

REFERENCES:

1. UNICEF U. Levels and trends in child mortality. Estimates developed by the UN inter-agency group for child mortality estimation. 2011.
2. Amouzou A, Velez LC, Tarekegn H, *et al*. One is too many: ending child deaths from pneumonia and diarrhoea. New York: Unicef, 2016. pp 73–75
3. Mathew JL, Patwari AK, Gupta P, *et al*. Acute respiratory infection and pneumonia in India: a systematic review of literature for advocacy and action: UNICEF-PHFI series on newborn and child health, India. *Indian Pediatr*2011; 48: 191–218.
4. Farooqui H, Jit M, Heymann DL, *et al*. Burden of severe pneumonia, pneumococcal pneumonia and pneumonia deaths in Indian states: modelling based estimates. *PLoS One* 2015

For peer review only

5. Rudan I, O'brien KL, Nair H,*et al*. Epidemiology and etiology of childhood pneumonia in 2010: estimates of incidence, severe morbidity, mortality, underlying risk factors and causative pathogens for 192 countries. *Journal of global health*. 2013 Jun;3(1).

6. Leung DT, Chisti MJ, Pavia AT. Prevention and control of childhood pneumonia and diarrhea. *Pediatric Clinics*. 2016 Feb 1;63(1):67-79.

7. Virkki R, Juven T, Rikalainen H, *et al*. Differentiation of bacterial and viral pneumonia in children. *Thorax*. 2002 May 1;57(5):438-41.

8. World Health Organization. Pneumococcal conjugate vaccines in infants and children under 5 years of age: WHO position paper–February 2019. Accessed 12th July 2019.

9. Government of India. Introduction of Pneumococcal Conjugate Vaccine: Operational Guidelines. Ministry of Health and Family Welfare, 2017 Available at: https://nhm.gov.in/New_Updates_2018/NHM_Components/Immunization/Guildelines_for_immunization/Operational_Guidelines_for_PCV_introduction.pdf. Accessed 12 July 2019.

10. Cherian T, Mulholland EK, Carlin JB, *et al*. Standardized interpretation of paediatric chest radiographs for the diagnosis of pneumonia in epidemiological studies. *Bulletin of the World Health Organization*. 2005;83:353-9

11. Pneumonia Vaccine Trial Investigators' Group. Standardization of Interpretation of Chest Radiographs for the Diagnosis of Pneumonia in Children. Geneva: Department of Vaccines and Biologicals, World Health Organization; 2001

12. Mahomed N, Fancourt N, De Campo J, *et al*. Preliminary report from the World Health Organization chest radiography in epidemiological studies project. *Pediatric radiology*. 2017 Oct 1;47(11):1399-404.

For peer review only - http://bmjopen.bmj.com/site/about/guidelines.xhtml

13. Greenberg D, Givon-Lavi N, Ben-Shimol S,*et al*. Impact of PCV7/PCV13 introduction on community-acquired alveolar pneumonia in children< 5 years. *Vaccine*. 2015 Aug 26;33(36):4623-9. 6.

14. Sutcliffe CG, Shet A, Varghese R, *et al*. Nasopharyngeal carriage of Streptococcus pneumoniae serotypes among children in India prior to the introduction of pneumococcal conjugate vaccines: a cross-sectional study. *BMC Infect Dis*. 2019 Dec;19(1):605.

15. Annual health survey (AHS) factsheet 2012–13 Uttar Pradesh [Internet]. New Delhi: Office of Registrar General & Census Commissioner, Ministry of Home Affairs, Government of India; 2010–2011. Available from:http://www.censusindia.gov.in/vital_statistics/AHSBulletins/AHS_Factsheets_2012-13/FACTSHEET-UTTAR_PRADESH.pdf. Accessed 5th August, 2019.

16. Annual health survey (AHS) factsheet 2012–13 Bihar [Internet]. New Delhi: Office of Registrar General & Census Commissioner, Ministry of Home Affairs, Government of India; 2010–2011. Available from:http://www.censusindia.gov.in/vital_statistics/AHSBulletins/AHS_Factsheets_2012-13/FACTSHEET-Bihar.pdf. Accessed 5th August, 2019.

17. Awasthi S, Pandey CM, Verma T, *et al*. Incidence of community-acquired pneumonia in children aged 2-59 months of age in Uttar Pradesh and Bihar, India, in 2016: An indirect estimation. *PloS one*. 2019 Mar 20;14(3):e0214086.

18. Awasthi S, Singh JV, Kohli N, *et al*. Hospital-based surveillance for radiological pneumonia in children under 5 years of age in Uttar Pradesh and Bihar. *Pediatric Infectious Disease*. 2016 Jun 30;8(2):52-7.

For peer review only

For peer review only - http://bmjopen.bmj.com/site/about/guidelines.xhtml

19. World Health Organization. Pocket Book of Hospital Care for Children: Guidelines for the Management of Common Childhood Illnesses. 2nd edn. Geneva, Switzerland: World Health Organization, 2013.

20. Charan J, Biswas T. How to calculate sample size for different study designs in medical research?. Indian journal of psychological medicine. 2013 Apr;35(2):121.

21. Integrated Management of Childhood Illness (IMCI) Chart Booklet [online] Geneva: World Health Organization. Available at: https://apps.who.int/iris/bitstream/handle/10665/104772/9789241506823_Chartbook_eng.pdf Accessed:19th December, 2019.

22. Bassignani MJ, Bubash-Faust L, Ciambotti J, *et al*. Conversion of teaching file cases from film to digital format: a comparison between use of a diagnostic-quality digitizer and use of a flatbed scanner with transparency adapter. *Acad Radiol*. 2003 May 1;10(5):536-42.

23. World Health Organization. The WHO Anthro Survey Analyser. In: World Health Organization [Internet]. Available: https://www.who.int/nutgrowthdb/software/en/. Accessed: 10th June, 2019.

24. Horton NJ, Kleinman KP. Much ado about nothing: A comparison of missing data methods and software to fit incomplete data regression models. The American Statistician. 2007 Feb 1;61(1):79-90.

25. SPSS Inc. Released 2009. SPSS for Windows [software], Version 22·0. Chicago, SPSS Inc. Available from:www.spss.com

26. von Elm E, Altman DG, Egger M, et al. The strengthening the reporting of observational studies in epidemiology (STROBE) statement: guidelines for reporting observational studies. J Clin Epidemiol 2008;61:344–9

For peer review only - http://bmjopen.bmj.com/site/about/guidelines.xhtml

547 27. Fancourt N, Deloria Knoll M, Baggett HC, *et al*. Chest radiograph findings in childhood pneumonia cases from the multisite PERCH study. *Clin Infect Dis*. 2017 May 29;64(suppl_3):S262-70

550 28. Enwere G, Cheung YB, Zaman SM, *et al*. Epidemiology and clinical features of pneumonia according to radiographic findings in Gambian children. *Trop Med Int Health*. 2007 Nov;12(11):1377-85.

553 29. Meiron Hassen, Alemayehu Toma, Mulugeta Tesfay, *et al*. Radiologic Diagnosis and Hospitalization among Children with Severe Community Acquired Pneumonia: A Prospective Cohort Study. *Biomed Res Int*, vol. 2019, Article ID 6202405, 8 pages, 2019. https://doi.org/10.1155/2019/6202405.

557 30. Kelly MS, Crotty EJ, Rattan MS, *et al*. Chest radiographic findings and outcomes of pneumonia among children in Botswana. *Pediatr Infect Dis J*. 2016 Mar;35(3):257.

559 31. da Fonseca Lima EJ, Mello MJ, Lopes MI, *et al*. Risk factors for community-acquired pneumonia in children under five years of age in the post-pneumococcal conjugate vaccine era in Brazil: a case control study. *BMC Pediatr*. 2016 Dec;16(1):157.

562 32. Sigauque B, Roca A, Bassat Q, *et al*. Severe pneumonia in Mozambican young children: clinical and radiological characteristics and risk factors. *J Trop Pediatr*. 2009 Apr 28;55(6):379-87.

565 33. Ismail SA, McCullough A, Guo S, *et al*. Gender-related differences in care-seeking behaviour for newborns: a systematic review of the evidence in South Asia. BMJ global health. 2019 May 1;4(3):e001309.

568 34. Fikree FF, Pasha O. Role of gender in health disparity: the South Asian context. BMJ 2004;328:823–6. 10.1136/bmj.328.7443.823

For peer review only - http://bmjopen.bmj.com/site/about/guidelines.xhtml

35. Asfaw A, Lamanna F, Klasen S. Gender gap in parents' financing strategy for hospitalization of their children: evidence from India. Health economics. 2010 Mar;19(3):265-79.

36. Pandey A, Sengupta PG, Mondal SK, *et al*. Gender differences in healthcare-seeking during common illnesses in a rural community of West Bengal, India. Journal of Health, Population and Nutrition. 2002 Dec 1:306-11.

37. Jana A, Basu R. Examining the changing health care seeking behavior in the era of health sector reforms in India: evidences from the National Sample Surveys 2004 & 2014. Global health research and policy. 2017 Dec;2(1):6.

38. Rambaud-Althaus C, Althaus F, Genton B, *et al*. Clinical features for diagnosis of pneumonia in children younger than 5 years: a systematic review and meta-analysis. *Lancet Infect Dis*. 2015 Apr 1;15(4):439-50

39. Awasthi S, Glick AH, Fletcher RH. Effect of cooking fuels on Respiratory Diseases in Pre-school children in Lucknow, India. *Am J Trop Med Hyg* 1996; 55: 48-51

40. Tielsch JM, Katz J, Thulasiraj RD, *et al*. Exposure to indoor biomass fuel and tobacco smoke and risk of adverse reproductive outcomes, mortality, respiratory morbidity and growth among newborn infants in south India. *Int J Epidemiol*. 2009 Sep 16;38(5):1351-63.

41. O'Brien KL, Baggett HC, Brooks WA, *et al*. Causes of severe pneumonia requiring hospital admission in children without HIV infection from Africa and Asia: the PERCH multi-country case-control study. *The Lancet*. 2019 Jun 27

42. World Health Organization. Pneumococcal conjugate vaccines in infants and children under 5 years of age: WHO position paper–February 2019. Accessed 12th March. 2019.

For peer review only - http://bmjopen.bmj.com/site/about/guidelines.xhtml

43. Balaji V, Jayaraman R, Verghese VP, Baliga PR, Kurien T. Pneumococcal serotypes associated with invasive disease in under five children in India & implications for vaccine policy. The Indian journal of medical research. 2015 Sep;142(3):286.

44. Arifeen SE, Saha SK, Rahman S, Rahman KM, Rahman SM, Bari S, Naheed A, Mannan I, Seraji MH, Ahmed NU, Shameem Hassan M. Invasive pneumococcal disease among children in rural Bangladesh: results from a population-based surveillance. Clinical Infectious Diseases. 2009 Mar 1;48 (Supplement_2):S103-13.

45. Johnson HL, Deloria-Knoll M, Levine OS, Stoszek SK, Hance LF, Reithinger R, Muenz LR, O'Brien KL. Systematic evaluation of serotypes causing invasive pneumococcal disease among children under five: the pneumococcal global serotype project. PLoS medicine. 2010 Oct 5;7(10):e1000348

46. Bénet T, Picot VS, Awasthi S, Pandey N, Bavdekar A, Kawade A, Robinson A, Rakoto-Andrianarivelo M, Sylla M, Diallo S, Russomando G. Severity of pneumonia in under 5-year-old children from developing countries: a multicenter, prospective, observational study. The American journal of tropical medicine and hygiene. 2017 Jul 12;97(1):68-76

Figure Legend

Figure 1: Flow diagram of cases of community acquired pneumonia recruited from participating districts before the introduction of pneumococcal conjugate vaccine (January 2015-April 2017)

For peer review only

For peer review only - http://bmjopen.bmj.com/site/about/guidelines.xhtml

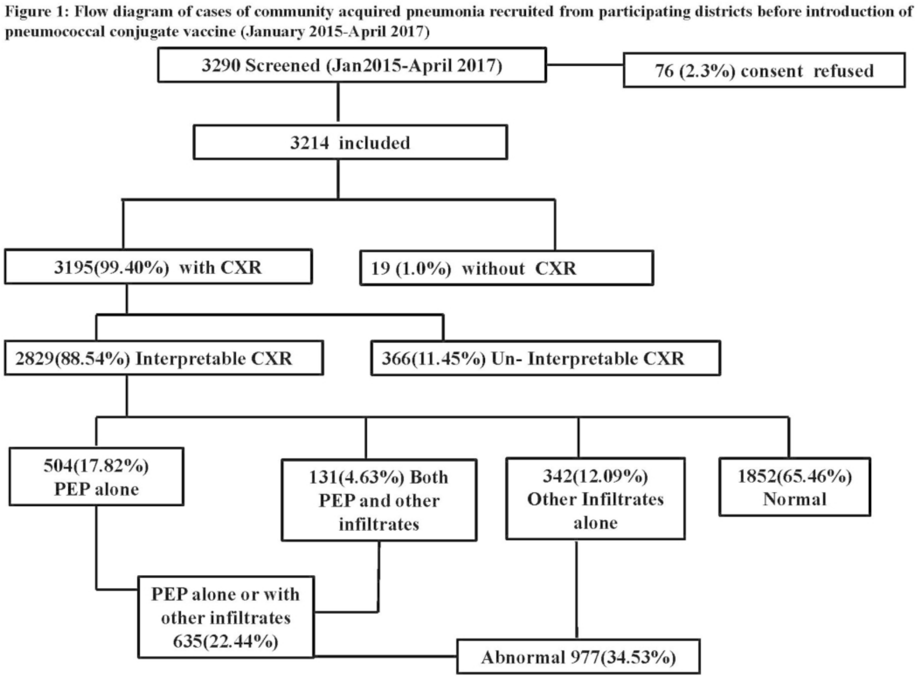

Figure 1: Flow diagram of cases of community acquired pneumonia recruited from participating districts before the introduction of pneumococcal conjugate vaccine (January 2015-April 2017)

254x190mm (300 x 300 DPI)

Childhood Pneumonia Surveillance

Department of Paediatrics, KGMU, Lucknow ,UP

<u>Form-R</u>

(RADIOLOGY REPORT FORM)

1	**Drs_ID**	[__][__][__][__]
	**Radiology Report**	**Patient Details**
2	**IDNo:**	[__] / [__] / [__][__][__]/[__][__][__][__]<br>State /District / Unit / Subject number<br>*(For office use)*
2	Date Of Report	[__][__]/[__][__]/[__][__][__][__] (DD/MM/YYYY)
	**Report Details**	**Findings (tick one)**
3	Image Quality	Adequate ☐ Suboptimal ☐ Un-interpretable ☐
4	Significant Pathology	Yes ☐ No ☐ Un-interpretable ☐
5	**End Point Consolidation**	
5a	Left	Yes ☐ No ☐ Un-interpretable ☐
5b	Right	Yes ☐ No ☐ Un-interpretable ☐<br>Uninterpretable
6	Other Infiltrates/Abnormalities	
6a	Left	Yes ☐ No ☐ Un-interpretable ☐
6b	Right	Yes ☐ No ☐ Un-interpretable ☐
7	Pleural Fluid	
7a	Left	Yes ☐ No ☐ Un-interpretable ☐
7b	Right	Yes ☐ No ☐ Un-interpretable ☐
8	Comments:	[ ]
9	Conclusion:	a) Primary endpoint pneumonia only ☐<br>b) Other infiltrate only ☐<br>c) Both PEP and other infiltrate ☐<br>d) Normal ☐<br>e) Un-interpretable for any findings☐

y only

STROBE Statement—Checklist of items that should be included in reports of ***cross-sectional studies***

	Item No	Recommendation	Page Number
**Title and abstract**	1	(*a*) Indicate the study's design with a commonly used term in the title or the abstract	1
		(*b*) Provide in the abstract an informative and balanced summary of what was done and what was found	2-3
**Introduction**			
Background/rationale	2	Explain the scientific background and rationale for the investigation being reported	4
Objectives	3	State specific objectives, including any pre-specified hypotheses	5
**Methods**			
Study design	4	Present key elements of study design early in the paper	5
Setting	5	Describe the setting, locations, and relevant dates, including periods of recruitment, exposure, follow-up, and data collection	5
Participants	6	(*a*) Give the eligibility criteria, and the sources and methods of selection of participants	5-6
Variables	7	Clearly define all outcomes, exposures, predictors, potential confounders, and effect modifiers. Give diagnostic criteria, if applicable	5-6
Data sources/ measurement	8*	For each variable of interest, give sources of data and details of methods of assessment (measurement). Describe comparability of assessment methods if there is more than one group	7-8
Bias	9	Describe any efforts to address potential sources of bias	25
Study size	10	Explain how the study size was arrived at	6
Quantitative variables	11	Explain how quantitative variables were handled in the analyses. If applicable, describe which groupings were chosen and why	11-13
Statistical methods	12	(*a*) Describe all statistical methods, including those used to control for confounding	11-13
		(*b*) Describe any methods used to examine subgroups and interactions	Table 1, 2 &3
		(*c*) Explain how missing data were addressed	12<br>reference 24
		(*d*) If applicable, describe analytical methods taking account of sampling strategy	11-13
		(*e*) Describe any sensitivity analyses	NA
**Results**			
Participants	13*	(a) Report numbers of individuals at each stage of study—eg numbers potentially eligible, examined for eligibility, confirmed eligible, included in the study, completing follow-up, and analysed	14
		(b) Give reasons for non-participation at each stage	Figure 1
		(c) Consider use of a flow diagram	Figure 1
Descriptive data	14*	(a) Give characteristics of study participants (eg demographic, clinical, social) and information on exposures and potential confounders	14-15<br>Table 1
		(b) Indicate number of participants with missing data for each variable of interest	Figure 1

For peer review only - http://bmjopen.bmj.com/site/about/guidelines.xhtml

Outcome data	15*	Report numbers of outcome events or summary measures	
Main results	16	(*a*) Give unadjusted estimates and, if applicable, confounder-adjusted estimates and their precision (eg, 95% confidence interval). Make clear which confounders were adjusted for and why they were included	Table 3 (adjusted odds ratio)
		(*b*) Report category boundaries when continuous variables were categorized	Table 3
		(*c*) If relevant, consider translating estimates of relative risk into absolute risk for a meaningful time period	NA (calculated only odds ratio)
Other analyses	17	Report other analyses done—eg analyses of subgroups and interactions, and sensitivity analyses	Table 3
**Discussion**			
Key results	18	Summarise key results with reference to study objectives	21-23
Limitations	19	Discuss limitations of the study, taking into account sources of potential bias or imprecision. Discuss both direction and magnitude of any potential bias	25-26
Interpretation	20	Give a cautious overall interpretation of results considering objectives, limitations, multiplicity of analyses, results from similar studies, and other relevant evidence	21-23
Generalisability	21	Discuss the generalisability (external validity) of the study results	21
**Other information**			
Funding	22	Give the source of funding and the role of the funders for the present study and, if applicable, for the original study on which the present article is based	27

*Give information separately for exposed and unexposed groups.

Note: An Explanation and Elaboration article discusses each checklist item and gives methodological background and published examples of transparent reporting. The STROBE checklist is best used in conjunction with this article (freely available on the Web sites of PLoS Medicine at http://www.plosmedicine.org/, Annals of Internal Medicine at http://www.annals.org/, and Epidemiology at http://www.epidem.com/). Information on the STROBE Initiative is available at www.strobe-statement.org.

For peer review only

BMJ Open

Chest Radiograph Findings in children aged 2-59 months hospitalized with Community-Acquired Pneumonia, prior to the introduction of Pneumococcal Conjugate Vaccine in India- A Prospective Multisite Observational Study

Journal:	*BMJ Open*
Manuscript ID	bmjopen-2019-034066.R3
Article Type:	Original research
Date Submitted by the Author:	12-Apr-2020
Complete List of Authors:	Awasthi, Shally; King George's Medical University, Pediatrics<br>Rastogi, Tuhina; King George's Medical University, Paediatrics<br>Mishra, Neha; King George's Medical University, Pediatrics<br>Chauhan, Abhishek ; Dr Ram Manohar Lohia Institute of Medical Sciences, Department of Radio-diagnosis<br>Mohindra, Namita; Sanjay Gandhi Post Graduate Institute of Medical Sciences, Department of Radio-diagnosis<br>Shukla, Ram ; Banaras Hindu University, Varanasi, India, Institute of Medical Sciences<br>Agrawal, Monika; King George's Medical University, Community Medicine and Public Health<br>Pandey, CM; Sanjay Gandhi Post Graduate Institute of Medical Sciences, Biostatistics & Health Informatics<br>Kohli, Neera ; King George's Medical University, Department of Radio-diagnosis<br>Study Group, CAP; CAP study Group
**Primary Subject Heading**:	Paediatrics
Secondary Subject Heading:	Global health, Public health, Radiology and imaging
Keywords:	Chest radiographs, Hospitalized community-acquired pneumonia, under-five, Streptococcus pneumoniae, India

SCHOLARONE™
Manuscripts

I, the Submitting Author has the right to grant and does grant on behalf of all authors of the Work (as defined in the below author licence), an exclusive licence and/or a non-exclusive licence for contributions from authors who are: i) UK Crown employees; ii) where BMJ has agreed a CC-BY licence shall apply, and/or iii) in accordance with the terms applicable for US Federal Government officers or employees acting as part of their official duties; on a worldwide, perpetual, irrevocable, royalty-free basis to BMJ Publishing Group Ltd ("BMJ") its licensees and where the relevant Journal is co-owned by BMJ to the co-owners of the Journal, to publish the Work in this journal and any other BMJ products and to exploit all rights, as set out in our licence.

The Submitting Author accepts and understands that any supply made under these terms is made by BMJ to the Submitting Author unless you are acting as an employee on behalf of your employer or a postgraduate student of an affiliated institution which is paying any applicable article publishing charge ("APC") for Open Access articles. Where the Submitting Author wishes to make the Work available on an Open Access basis (and intends to pay the relevant APC), the terms of reuse of such Open Access shall be governed by a Creative Commons licence – details of these licences and which Creative Commons licence will apply to this Work are set out in our licence referred to above.

Other than as permitted in any relevant BMJ Author's Self Archiving Policies, I confirm this Work has not been accepted for publication elsewhere, is not being considered for publication elsewhere and does not duplicate material already published. I confirm all authors consent to publication of this Work and authorise the granting of this licence.

For peer review only

Title: *Chest Radiograph Findings in children aged 2-59 months hospitalized with Community-Acquired Pneumonia, prior to the introduction of Pneumococcal Conjugate Vaccine in India - A Prospective Multisite Observational Study*

Short Title: *Radiological Findings in Children hospitalized with Community-Acquired Pneumonia in India Pre-Pneumococcal Conjugate Vaccine introduction*

Shally Awasthi[1]*, Tuhina Rastogi[1], Neha Mishra[1], Abhishek Chauhan[2], Namita Mohindra[3], Ram Chandra Shukla[4], Monika Agarwal[5], Chandra Mani Pandey[6], Neera Kohli[7] and CAP Group^

[1]Department of Pediatrics, King George's Medical University, Lucknow, India
[2]Department of Radio-diagnosis, Dr. Ram Manohar Lohia Institute of Medical Sciences, Lucknow, India
[3]Department of Radio-diagnosis, Sanjay Gandhi Post Graduate Institute of Medical Sciences, Lucknow, India
[4]Department of Radio-diagnosis, Institute of Medical Sciences, Banaras Hindu University, Varanasi, India
[5] Department of Community Medicine and Public Health, King George's Medical University, Lucknow, India
[6]Department of Biostatistics and Health Informatics, Sanjay Gandhi Postgraduate Institute of Medical Sciences, Lucknow, India
[7]Department of Radio-diagnosis, King George's Medical University, Lucknow, India

^Members of the Lucknow CAP Group are given in the Acknowledgement

*Corresponding Author
Postal Address: Prof. Shally Awasthi, Head, Department of Pediatrics, King George's Medical University, Lucknow, India. Pincode: 226003
Mobile: +91-9839221244
E-mail: shally07@gmail.com

Word Count: 4808 Tables: 3 Figure: 1

For peer review only - http://bmjopen.bmj.com/site/about/guidelines.xhtml

ABSTRACT

Objectives: The current study was a hospital-based surveillance of cases hospitalized with World Health Organization defined community-acquired pneumonia in children, aged 2-59 months, to assess the radiological abnormalities in chest X-rays and to identify the demographic and clinical correlates of specific radiological abnormalities, in residents of pre-specified districts of Uttar Pradesh and Bihar, India.

Design: Prospective, active, hospital-based surveillance.

Setting: Multisite study conducted in a network of 117 secondary/tertiary care hospitals in four districts of Uttar Pradesh and Bihar, India.

Participants: Included were children aged 2-59 months, hospitalized with community-acquired pneumonia, residing in the project district, with duration of illness <14 days and who had not been hospitalized elsewhere for this episode nor had been recruited previously.

Main outcome measure: Concordant radiological abnormalities in the chest X-rays.

Results: From January 2015 to April 2017, 3214 cases were recruited and in 99.40 % (3195/3214) chest X-rays were available, among which 88.54 % (2829/3195) were interpretable. Relevant radiological abnormalities were found in 34.53 % (977/ 2829, 95% C.I. 32.78 -36.28). These were primary end-point pneumonia alone or with other infiltrates in 22.44 % (635/2829, C.I. 20.90 %-23.98 %) and other infiltrates in 12.09% (342/ 2829; C.I. 10.88 %- 13.29 %). There was a statistically significant inter-district variation in radiological abnormalities. Statistically significantly higher proportion of abnormal chest X-ray were found in girls, those with weight-for-age z score ≤-3 SD, longer duration of fever, pallor and with exposure to biomass fuel.

For peer review only - http://bmjopen.bmj.com/site/about/guidelines.xhtml

Conclusions: Among hospitalized cases of community-acquired pneumonia, almost one-third children had abnormal chest radiographs, which were higher in females, malnourished children and those with longer illnesses; and an intra-district variation was observed.

Key words: Chest radiographs, Hospitalized community-acquired pneumonia, under-five, *Streptococcus pneumoniae*, India

Strengths and Limitations of the Study

- Prospective, multisite study recruiting cases from a large hospital surveillance network established for the project in four districts in two states of India that have high under-five mortality rates.
- World Health Organization definition of clinical pneumonia was used for identifying hospitalized cases for generalizability.
- Radiological abnormalities were interpreted by a panel of three independent, trained radiologists outside the surveillance network, blinded to each other as well as clinical features of the case.
- Since pre-existing X-rays machines were used, there were variations in the quality of images, which was, however, minimized by digitizing them centrally.
- Since data of clinical examination was abstracted from hospital records, inter-observer variation in documentation was possible.

For peer review only - http://bmjopen.bmj.com/site/about/guidelines.xhtml

INTRODUCTION

Community-acquired pneumonia (CAP) is the leading cause of death in young children worldwide. Globally, pneumonia accounts for 16% of deaths in children under-five years of age, which translates into almost one million deaths annually, with 0.9 million deaths reported in 2016. [1 2] Most deaths due to pneumonia occur in low and middle income countries, particularly in sub-Saharan Africa and South Asia. [2 3] In India, there were approximately 0·44 million under-five deaths due to CAP in the year 2015. [4]

CAP could be of either viral or bacterial etiology. [5 - 7] In young children, bacteria associated with pneumonia are predominantly *Streptococcus pneumoniae* and *Hemophilus influenzae Type B*, while viruses are Respiratory Syncytial Virus and Influenza A or B. [6] However, etiology varies from country to country and also across different time periods. To reduce the incidence of bacterial pneumonia, vaccination against *Hemophilus influenzae Type B* is already under the national immunization programme of India since 2011. Thereafter, World Health Organization (WHO) introduced Pneumococcal Conjugate Vaccine (PCV) in countries, such as India, with high child mortality rates. [8] Consequently, PCV-13 was launched in May 2017 under the national immunization programme of five Indian states (Uttar Pradesh, Bihar, Rajasthan, Madhya Pradesh and Himachal Pradesh) in a phased manner. [9] It is expected to be rolled out in other parts of the country in the near future.

Differentiating bacterial from viral etiology of CAP based on clinical features or investigations remains difficult. [7 10 11] Therefore, several PCV probe trials have used radiographically

For peer review only - http://bmjopen.bmj.com/site/about/guidelines.xhtml

confirmed end-point pneumonia to be a surrogate marker of bacterial etiology and hence used this as an outcome measure for vaccine efficacy. This approach has been endorsed by WHO. [12-14]

The current study was conducted to assess the radiological abnormalities in chest X-rays (CXRs) and to identify the demographic and clinical correlates of specific radiological abnormalities in children aged 2-59 months, hospitalized with WHO defined CAP, residing in pre-specified districts of Uttar Pradesh and Bihar.

METHODS

Study design and Setting

This was a prospective, multisite observational study conducted in the northern Indian states of Uttar Pradesh and Bihar. Uttar Pradesh is the first most populated and Bihar third most populated state of the country.[15 16] This study was conducted in Lucknow and Etawah districts of Uttar Pradesh and Patna and Darbhanga districts of Bihar, India. In Lucknow district 66.2% population is urban and in Patna district 43.07%. [15 16] In contrast, only 22.3% population of Etawah district and 9.74% population of Darbhanga district is urban. [15 16] All four project districts have high infant and child mortality rates. [15-17] Infant mortality rate per 1000 live births of Lucknow district is 44, Etawah district 56, Patna district 31 and Darbhanga district 44, all being higher than the national average 41. [15-17] Similarly, under-five mortality rates per 1000 live births of districts included in this study are above the national average 50, being 58 for Lucknow, 85 for Etawah, 46 for Patna and 77 for Darbhanga. [15-17]

For peer review only - http://bmjopen.bmj.com/site/about/guidelines.xhtml

Study Population

This study was conducted after obtaining institutional ethical clearance from all four participating academic institutions, one in each district. Each institution then established a prospective, active, hospital-based surveillance system for this study. [17,18] After obtaining written informed consent from the private hospital management and district administration for public hospitals, included in the surveillance were 117 public and private hospitals of four study districts which provided either secondary or tertiary level care to admitted children.

Surveillance officers of the project visited these hospitals every 48-72 hours to screen and recruit eligible cases. In between the scheduled visits they telephonically contacted the hospitals daily to inquire about hospitalization of any potentially eligible case and made additional visits, if required. All children between the ages of 2-59 months, hospitalized in network hospitals with history of fast breathing with/without chest in-drawing were screened. [18]

Included were children hospitalized with symptoms of WHO defined CAP and residing in the project district. [18] WHO defined CAP was categorized into pneumonia and severe pneumonia. Fast breathing ≥ 50 breaths/minute in a child aged 2–11 months and ≥ 40 breaths/minute in a child aged 12-59 months, with or without chest in-drawing was categorized as `pneumonia`.[19] Cough or difficulty in breathing plus at least one of the following: (a) oxygen saturation < 90% or central cyanosis or (b) severe respiratory distress (e.g. grunting, very severe chest in-drawing) or (c) signs of pneumonia with a general danger sign (inability to breastfeed or drink, lethargy or reduced level of consciousness, convulsions) was categorized as `severe pneumonia`.[19] Excluded were children with cough for ≥ 14 days or those that had been hospitalized in last 14 days. [18]

For peer review only

For peer review only - http://bmjopen.bmj.com/site/about/guidelines.xhtml

Sample Size

We assumed that the incidence of radiological pneumonia is 3/100 child years of observations. Then for a margin of error of 1.5/100 child years of observation, incidence of pneumonia in the community of 20/100 child years of observation, alpha level of 0.05, and power of 90% when the estimated population of children under-five years of age in Lucknow district [20] is 750,000; 693 cases had to be included per district.

Data collection

Data was collected by surveillance officers hired for the project at each of the four district sites. They had postgraduate degree in social sciences and at least 10 years experience in community based health research. After recruitment, they were imparted six-day centralized training on project procedures and logistics. Classroom as well as practical skills training in real life setting was given by the coordinating centre in Lucknow. Pre and post tests were conducted to ascertain knowledge. Skills acquired by them were assessed during field observations. The coordinating centre provided annual refresher training to the surveillance officers from all four district sites in Lucknow. This was done to ensure quality of data collected.

After obtaining written, informed consent of the caregivers, data was collected through face-to-face interviews with caregivers, as well as by abstraction from hospital records. Socio-demographic data obtained by interviewing caregivers was: child`s age, gender, residence, birth order, immunization status, current breastfeeding status, parental education and occupation, smoking status of parents, family type, housing infrastructure, use of biomass fuel etc. Caregivers were also asked about the symptoms of disease and its duration in days.

For peer review only - http://bmjopen.bmj.com/site/about/guidelines.xhtml

Clinical data, recorded by pre-existing hospital staff at the time of hospitalization, was abstracted by surveillance officers. Data was collected on anthropometry (weight and height), fever (axillary temperature ≥37.5°C), oxygen saturation by pulse oxymetry where done, pallor, central cyanosis, signs of pneumonia along with general danger sign and vital signs (heart rate and respiratory rate). Presence of auscultatory wheeze was abstracted or inquired from the treating clinician. In case information on a clinical variable was missing in the medical chart, the surveillance officers contacted the clinician and obtained the same. Thus, there was no missing data for clinical variables reported in this manuscript.

At the hospitals, clinicians generally used Integrated Management of Childhood Illness definitions [21] to identify pallor, cyanosis, wheeze on auscultation and general danger sign as it is incorporated in their medical undergraduate training. Most clinicians of public health sector had also received a formal in-service training on Integrated Management of Childhood Illness. [21] Clinical outcome (survival or mortality) was noted from the hospital records on follow up.[17 18]

Chest x-ray (CXR) image acquisition and archiving

CXR (poster-anterior view) was done on the advice of treating physician. These CXRs were obtained by the surveillance officers at the time of recruitment. CXRs were either analog or digital. In case of digital CXRs, second copy was obtained where possible. If only single analog image was available, then the hardcopy of CXR was obtained from the caregiver after the child was discharged. If the caregiver was not ready to give the hardcopy of CXR (in <1% cases),

For peer review only - http://bmjopen.bmj.com/site/about/guidelines.xhtml

image of the same was captured by surveillance officers using 16 megapixel cell phone camera and portable CXR view-box.

CXRs of recruited cases were subsequently scanned and converted into digital format using a diagnostic quality film image digitizer (Microteck International Limited ®, Medi 6000 plus). [22] These were archived for web-based radiological interpretation. Digital images were stored in JPEG format at 300 dpi resolution. Each CXR file was anonymized and given a unique identification number. Digital CXRs were uploaded on customized online data management software.

Interpretation of radiological images

A panel of radiologists was constituted for standardized interpretations of CXRs. Four radiologists were part of this panel, one of whom was Project co-investigator-Radiology (NK). All radiologists are faculty in medical teaching institutes and also look after pediatric radiology. They have more than fifteen years experience in interpreting pediatric CXRs.

Radiologists were trained according to the methodology developed by Department of Immunization, Vaccines and Biologicals of the WHO for research purpose. [11] An international WHO-certified trainer from the International Centre for Diarrhoeal Disease Research, Bangladesh imparted two-day in-house training to the radiologists. The objectives of this training were to standardize interpretation and coding of CXRs, to develop a CXR reporting form **[S1 Appendix]** and to provide training on web-based CXR retrieval and reporting system. During the training, 210 CXRs of the WHO data set were used. For assessing post training concordance,

For peer review only - http://bmjopen.bmj.com/site/about/guidelines.xhtml

another set of 48 CXRs was provided for interpretation to individual radiologists. Post-test agreement with WHO findings was about 80%. Inter-observer variation was about 25% and was for only minor interpretations such as quality of film, end-point infiltrates etc. Repeat training was conducted on an additional set of 44 CXRs provided by the WHO to ensure standardization in interpretation. Thereafter, concordance achieved by the radiologists was reviewed quarterly by the study arbitrator. Radiologists met annually to review key concepts and discuss challenges faced in interpreting CXRs.

After training, radiologists independently reviewed CXRs and registered their findings in an online standardized chest radiograph interpretation form **[S1 Appendix].** For optimal viewing of CXRs, all radiologists used similar workstations. Specifications were provided for the computer monitor and hardware to be used. It was ensured that computer monitors had the correct brightness and contrast adjustment for optimal viewing.

During online evaluation, radiologists reported the quality of film as `*interpretable*` or `*un-interpretable*`. Further, they categorized `*interpretable*` CXRs as either `adequate/optimal` which allowed for confident interpretation of consolidation and pleural effusion as well as other infiltrates or `suboptimal` which allowed interpretation of only consolidation and pleural effusion, but not of other infiltrates. In `*un-interpretable*` CXRs, no comment was possible for radiological abnormality such as consolidation, pleural effusion or other infiltrates. [12]

After interpreting film quality, radiologists evaluated interpretable CXRs for abnormal radiological findings. For each CXR evaluated, radiological abnormality could be presence of

For peer review only - http://bmjopen.bmj.com/site/about/guidelines.xhtml

consolidation, other infiltrates or pleural effusion. `*Consolidation*` was defined as a dense or confluent opacity that occupied a portion or whole of a lobe or the entire lung that may or may not contain air bronchograms. `*Other infiltrates*` were defined as linear and patchy opacities (interstitial infiltrate) in a lacy pattern, featuring peri-bronchial thickening and multiple areas of atelectasis, also including minor patchy infiltrates that were not of sufficient magnitude to constitute end-point consolidation, and small areas of atelectasis which may be difficult to distinguish from consolidation. `*Pleural effusion*` was defined as the fluid in the lateral pleural space between the lung and chest wall that was spatially associated with a pulmonary parenchymal infiltrate (including `other infiltrates`) or had obliterated enough of the hemithorax to obscure any infiltrates. In most cases, this was to be seen at the costo-phrenic angle or as a layer of fluid adjacent to the lateral chest-wall and this does not include fluid seen in the horizontal or oblique fissures.[12] Primary end-point pneumonia (PEP) for research purpose was the presence of consolidation or pleural effusion which could be with or without other infiltrates.

Final conclusions were categorised as: (a) `*Abnormal*` when it was `PEP only` or `other infiltrates only` or `Both PEP and other infiltrates` and (b) `*Normal*` when no abnormal findings were seen. [12]

Data manager checked for inconsistencies and completeness after online evaluation of CXRs by individual radiologists. Thereafter, CXRs with concordant and discordant interpretations were identified. Interpretations were considered concordant when there was an agreement between two or more radiologists on final conclusions and discordant if all the three radiologists

For peer review only - http://bmjopen.bmj.com/site/about/guidelines.xhtml

disagreed. Discordant interpretations were forwarded to the study arbitrator (NK). Arbitrator assessed discordant CXRs online and her interpretation was taken as final.

Data management and statistical analysis

Clinical data of hospital surveillance network was entered online in customized software. Primary entry was done by the four participating sites. Secondary data entry was done by the coordinating site in separate customized software. Anonymized CXRs were uploaded on customized software. Each of the three panelists assessed the CXRs online, blind to peer assessments as well as clinical features of the case. CXR assessment data was downloaded from the online software in MS Access database.

Exploratory data analysis was performed for detection of outlier and missing observations for all the variables. Un-interpretable CXRs were not analyzed. Among interpretable CXRs, concordant radiological abnormalities were taken as final. Weight-for-age (WAZ) z-score each child was calculated using WHO Anthro Survey Analyser. [23] Weight of 7.59% (215/2829) children was missing. Missing weight was estimated using regression based imputation technique. [24] Kappa statistics was performed for agreement analysis among radiologists for CXRs findings. Statistical analysis was performed using SPSS version 22.0 (Chicago, IL). [25] A p-value of $<0{\cdot}05$ was taken as statistically significant using a two-tailed distribution.

Univariate analysis was performed to evaluate heterogeneity, stratified by four participating districts for socio-demographic variables such as child`s age, gender, residence, birth order, immunization status, current breastfeeding status, parental education and occupation, smoking

For peer review only - http://bmjopen.bmj.com/site/about/guidelines.xhtml

status of parents, family type, housing infrastructure, use of biomass fuel and for clinical variables such as weight, height, duration of fever and oxygen saturation.

We report proportions of radiological abnormalities among children hospitalized for CAP by four districts. Univariate analysis was performed to assess association of socio-demographic variables and clinical signs of CAP with radiological abnormalities. Chi-square test was used for categorical variables and student's t-test for continuous variables. ANOVA was used to test the significance of continuous variables when there were more than two groups. Multivariate unconditional logistic regression was performed to find association of presence of various radiological abnormalities with other variables that had univariate association with radiological abnormalities (p value $\leq$ 0.2) and/or were clinically meaningful, controlling for district of residence.

We developed four models in which the dependent (outcome) were different CXR findings and these were as follows:

Model I: Abnormal versus Normal

Model II: Primary End-Point Pneumonia alone or with Other Infiltrates versus Normal

Model III: Primary End-Point Pneumonia alone versus Normal

Model IV**:** Other Infiltrates only versus Normal

Independent variables were the same in all the four models. These were participating districts, age, gender, use of biomass fuel, symptoms of CAP such as duration of illness, presence of

For peer review only - http://bmjopen.bmj.com/site/about/guidelines.xhtml

wheeze on auscultation, pallor, vomiting everything and malnutrition status of the case [WAZ≤ -2 SD (malnourished) and WAZ ≤ -3 SD (severely malnourished)].

Patient and public involvement in research

Patients or public were not involved in the development of research question, study design or conducting the research. Reporting of this research conforms to the guidelines for Strengthening the Reporting of Observational Studies in Epidemiology (STROBE) [26]**.**

RESULTS

From January 2015 to April 2017, 3290 cases were screened in hospital surveillance network of four districts. Out of these, 3214 were eligible and consenting for inclusion **[Figure 1].** Among them, in 3195 (99.40%) CXR was done and only in 19 (1.0%) cases CXR not done. However, only 88.54% (2829/3195) CXRs were interpretable and remaining 11.45% (366/3195) were un-interpretable. In cases with interpretable CXRs, 99.11 % (2804/2829) had `severe pneumonia` as per the WHO criteria [19].

Concordance among ≥ 2 radiologists on final conclusion of CXRs findings was 86.0%. Kappa statistics was calculated for agreement of CXRs findings between Reader 1 versus Reader 2 (K_1=0.31), Reader 2 versus Reader 3 (K_2=0.46) and Reader 3 versus Reader 1(K_3=0.42). Among interpretable CXRs, 22.44% (635/2829) cases had PEP alone or with other infiltrates, 12.09% (342/2829) had other infiltrates only and 65.46% (1852/2829) were normal [**figure 1**].

For peer review only - http://bmjopen.bmj.com/site/about/guidelines.xhtml

Table 1 shows univariate distribution of socio-demographic and clinical variables among hospitalized cases across participating districts. A variation was observed in socio-demographic variables such as place of residence, type of house, type of family, maternal and paternal education and occupation, use of biomass fuel and parental smoking status across the four districts. We also report clinical variables of recruited cases across the four districts in **table 1**. Among those where pulse-oximetry was done, the proportion of cases with oxygen saturation < 90 % was found to be different across four districts.

Table 1: Distribution of socio-demographic and clinical variables among hospitalized children for participating districts (January 2015-April 2017)

**Characteristics**	**Lucknow**	**Etawah**	**Patna**	**Darbhanga**	**Total**
**Socio-demographic Characteristics**	**n=1025 (%)**	**n=389 (%)**	**n=744 (%)**	**n=671 (%)**	**N=2829 (%)**
**Gender**					
Male	659(64.29)	287(73.78)	557(74.87)	502(74.81)	2005(70.87)
**Place of residence**					
Rural	195(19.02)	279(71.72)	304(40.86)	614(91.51)	1392(49.20)
Urban	830(80.98)	110(28.28)	440(59.14)	57(8.49)	1437(50.80)
**Family Type**					
Joint	688(67.12)	360(92.54)	707(95.03)	383(57.08)	2138(75.57)
Nuclear	337(32.88)	29(7.46)	37(4.97)	287(42.77)	690(24.39)
**House type**					
Mud	64(6.24)	54(13.88)	123(16.53)	374(55.74)	615(21.74)
Bricks	854(83.32)	256(65.81)	453(60.89)	85(12.67)	1648(58.25)
Combined	107(10.4)	79(20.31)	168(22.58)	212(31.59)	566(20.01)
**Mother's Education**					
No formal education	203(19.80)	56(14.40)	328(44.09)	496(73.92)	1083(38.28)
Class I-V	108(10.54)	28(7.20)	82(11.02)	38(5.66)	256(9.05)
Class VI-XII	379(36.98)	176(45.24)	243(33.66)	112(16.69)	910(32.17)
Graduate/ Post graduation	335(32.68)	129(33.16)	91(12.23)	25(3.73)	580(20.50)
**Father's Education**					
No formal education	167(16.29)	29(7.46)	153(20.56)	345(51.42)	694(24.53)
Class I-V	85(8.29)	19(4.88)	91(12.23)	82(12.22)	277(9.79)
Class VI-XII	437(42.63)	206(52.96)	328(44.09)	205(30.55)	1176(41.57)
Graduate/ Post graduation	336(32.78)	135(34.70)	172(23.12)	39(5.81)	682(24.11)

For peer review only

For peer review only - http://bmjopen.bmj.com/site/about/guidelines.xhtml

**Birth Order**					
1st	435(42.44)	187(48.07)	315(42.34)	192(28.61)	1129(39.91)
2nd	343(33.46)	120(30.85)	235(31.59)	258(38.45)	956(33.79)
3rd	153(14.93)	47(12.08)	129(17.34)	137(20.42)	466(16.47)
More than 3rd	93(9.07)	35(9.00)	62(8.33)	83(12.37)	273(9.65)
**Immunization Status**					
Complete for age	792(77.27)	300(77.12)	711(95.56)	544(81.07)	2347(82.96)
Incomplete for age	220(21.46)	84(21.59)	25(3.36)	126(18.78)	455(16.08)
Unimmunized	13(1.27)	5(1.29)	8(1.08)	1(0.15)	27(0.95)
**Currently Breast Feeding**					
Yes	653(63.71)	256(65.81)	589(79.17)	537(80.03)	2035(71.93)
No	372(36.29)	133(34.19)	155(20.83)	134(19.97)	794(28.07)
**Father's Occupation**					
Unemployed	13(1.27)	20(5.14)	27(3.63)	63(9.39)	123(4.35)
Daily wages	329(32.10)	81(20.82)	165(22.18)	474(70.64)	1049(37.08)
Salaried/ Professional	397(38.73)	104(26.74)	245(32.93)	55(8.20)	801(28.31)
Self-Employment	286(27.90)	184(47.30)	307(41.26)	79(11.77)	856(30.26)
**Mother's Occupation**					
Home maker	961(93.76)	376(96.66)	701(94.22)	484(72.13)	2522(89.15)
Daily wages	17(1.66)	3(0.77)	17(2.28)	171(25.48)	208(7.35)
Salaried/Professional	47(4.59)	9(2.31)	18(2.42)	7(1.04)	81(2.86)
Self-Employment	0(0.0)	1(0.26)	8(1.08)	9(1.34)	18(0.64)
**Biomass fuel**					
Yes	211(20.59)	245(62.98)	263(35.35)	609(90.76)	1328(46.94)
No	814(79.41)	144(37.02)	481(64.65)	62(9.24)	1501(53.06)
**Smoking Status-Father**					
Yes	152(14.83)	45(11.57)	56(7.53)	59(8.79)	312(11.03)
No	873(85.17)	344(88.43)	688(92.47)	612(91.21)	2517(88.97)
**Indoor smoking-Father**					
Yes	83(8.10)	21(5.40)	16(2.15)	43(6.41)	163(5.76)
No	942(91.90)	368(91.60)	728(97.85)	628(93.59)	2666(94.24)
**Smoking Status-Family member**					
Yes	129(12.59)	55(14.14)	45(6.05)	102(15.20)	331(11.70)
No	896(87.41)	334(85.86)	699(93.95)	569(84.80)	2498(83.30)
**Indoor smoking – Family member**					
Yes	84(8.20)	27(6.94)	27(3.63)	94(14.01)	232(8.20)
No	941(91.80)	362(93.06)	717(96.37)	577(85.99)	2597(91.80)
**Clinical Variables at**	**n**	**n**	**n**	**n**	**n**

For peer review only - http://bmjopen.bmj.com/site/about/guidelines.xhtml

**the time of admission at hospital**	**Mean± SD**	**Mean± SD**	**Mean± SD**	**Mean± SD**	**Mean± SD**
Age (months)	1025<br>14.53±13.88	389<br>10.69±10.95	744<br>10.26±11.35	671<br>12.30±13.29	2829<br>12.35±12.85
Height (cm)	303<br>68.61±13.78	324<br>70.66±13.75	34<br>64.38±10.25	266<br>70.46±12.14	927<br>69.70±13.26
Weight (kg)	1025<br>7.96±2.97	389<br>7.34±2.73	744<br>7.11±2.78	671<br>7.78±2.93	2829<br>7.61±2.90
Fever Duration (days)	929<br>4.46±2.71	321<br>3.59±2.37	689<br>4.25±2.52	569<br>3.54±2.47	2508<br>4.08±2.59
**Respiratory Rate**					
Respiratory Rate (2-11 months)	602<br>53.38±14.05	272<br>60.87±9.60	540<br>53.82±10.16	451<br>60.78±7.26	1864<br>56.37±11.49
Respiratory Rate (12-59 months)	423<br>47.75±14.17	117<br>53.22±13.17	204<br>45.59±10.11	220<br>58.03±6.83	964<br>50.30±12.76
Oxygen saturation done (n, %)	528 (51.51)	343 (88.17)	236 (34.25)	319 (56.06)	1426 (50.40)
Oxygen saturation < 90% (n, %)	61(11.53)	57(16.61)	49 (20.76)	43(13.47)	210 (14.72)
Grunting (n, %)	461(44.98)	353 (90.75)	687 (92.34)	649 (96.72)	2150 (76.00)
Very severe chest in-drawing (n, %)	953 (92.97)	352 (90.49)	739 (99.33)	651 (97.02)	2695 (95.26)
**Signs of Pneumonia with a general danger sign**					
Lethargy or reduced level of consciousness (n, %)	423 (41.27)	259 (66.58)	6 (0.81)	412 (61.40)	1100(38.88)
Inability to breastfeed or drink (n, %)	291(28.39)	259 (66.58)	75 (10.08)	312 (46.50)	937(33.12)
Convulsions (n, %)	16 (1.56)	19 (4.58)	13 (1.75)	100 (14.90)	148(5.23)
Central cyanosis (n,%)	15 (1.46)	7 (1.80)	26 (3.49)	14 (2.09)	62 (2.19)

Among those with interpretable CXRs, 34.5% (977/2829) had radiological abnormalities, which were PEP alone or with other infiltrates in 64.9% (635/977) and other infiltrates in 35.1% (342/977) (**table 2).** In the same table, we report these findings by district as well as socio-demographical and clinical associates of normal versus abnormal CXR as well among those with abnormal CXRs, in those with PEP alone or with other infiltrates versus other infiltrates only.

For peer review only - http://bmjopen.bmj.com/site/about/guidelines.xhtml

For peer review only

We observed statistically significant district-wise heterogeneity in radiological abnormalities (**table 2**). We found higher proportion of radiological abnormalities as well as PEP alone or with other infiltrates in Patna and Lucknow districts, and lower proportion in Etawah and Darbhanga districts. Statistically significant higher proportion of females hospitalized for CAP had radiologically abnormal CXR (**table 2**). Likewise, statistically significantly higher proportion of abnormal CXR findings were reported in hospitalized cases who had symptoms of fever, pallor, wheezing on auscultation, vomiting everything or were malnourished (**table 2**). Among cases with abnormal CXRs, statistically significantly higher proportion of cases with other infiltrates had wheezing on auscultation.

Table 2: Distribution of socio-demographic and clinical factors by chest radiograph findings among hospitalized children from January 2015-April 2017

	**Interpretable chest X rays**				**Abnormal chest X rays**		
	**N=2829**	**Normal**<br>**1852**<br>**n (%)**	**Abnormal**<br>**977**<br>**n (%)**	**p value**	**PEP* alone or with other infiltrates**<br>**635**<br>**n (%)**	**Other infiltrates**<br>**342**<br>**n (%)**	**p value**
**Participating site** (row %)							
Lucknow	1025	636 (62.05)	389 (37.95)	<0.0001	282 (72.49)	107 (27.51)	<0.0001
Etawah	389	275 (70.69)	114 (29.31)		73 (64.04)	41 (35.96)	
Patna	744	457 (61.42)	287 (38.58)		184 (64.11)	103 (35.89)	
Darbhanga	671	484 (72.13)	187 (27.87)		96 (51.34)	91 (48.66)	
**Socio-demographic & clinical factors** (column %)							

For peer review only - http://bmjopen.bmj.com/site/about/guidelines.xhtml

**Age-group (months)**							
2-11	1865	1223 (66.04)	642 (65.71)	0.86	409 (64.41)	233 (68.13)	0.26
12-59	964	629 (33.96)	335 (34.29)		226 (35.59)	109 (31.87)	
**Gender**							
Male	2005	1354 (73.11)	651 (66.63)	<0.0001	426 (67.09)	225 (65.79)	0.72
Female	824	498 (26.89)	326 (33.37)		209 (32.91)	117 (34.21)	
**Place of residence**							
Rural	1392	921 (49.73)	471 (48.21)	0.44	299 (47.09)	172 (50.29)	0.34
Urban	1437	931 (50.27)	506 (51.79)		336 (52.91)	170 (49.71)	
**Biomass fuel**							
Yes	1501	867 (46.81)	461 (47.19)	0.44	294 (42.30)	167 (48.83)	0.24
No	1328	985 (53.19)	516 (52.81)		341 (53.70)	175 (51.17)	
**Immunization status**							
Complete for age	2347	1546 (83.48)	801 (81.99)	0.32	516 (81.26)	285 (83.33)	0.54
Incomplete	482	306 (16.52)	176 (18.01)		119 (18.74)	57 (16.67)	
**Clinical Features**							
Fever	2499	1616 (87.26)	883 (90.38)	0.014	575 (90.55)	308 (90.06)	0.82
Pallor	764	465 (25.11)	299 (30.60)	0.002	200 (31.50)	99 (28.95)	0.41
Wheeze on auscultation	2054	1377 (74.35)	677 (69.29)	0.005	415 (65.35)	262 (76.61)	0.0003
Duration of illness fever [days] (n, Mean ± SD)	2499	1611 3.91±2.51	888, 4.40±2.70	<0.0001	577, 4.57±2.82	342, 4.08±2.44	0.011
**Respiratory Rate**							
Respiratory Rate [2-11 months] (n, Mean ± SD)	1865	1243 55.52±11.29	642 57.99±11.70	<0.0001	409 58.12±11.88	233 57.74±11.40	0.69
Respiratory Rate [12-59 months] (n, Mean ± SD)	964	629 49.78±12.41	335 51.28±13.37	0.08	226 51.35±13.31	109 51.12±13.35	0.88
Fast Breathing for	1735	1130	605	0.11	384	221	0.69

For peer review only

age [2-11 months]		(61.02)	(61.92)		(60.47)	(64.62)	
Fast Breathing for age [12-59 months]	862	562 (30.35)	300 (30.71)	0.92	204 (32.13)	96 (28.07)	0.53
**Signs of Pneumonia with a general danger sign n (%)**							
Lethargy or reduced level of consciousness	1101	732 (39.52)	369 (37.77)	0.39	247 (38.90)	122 (35.67)	0.33
Inability to breastfeed or drink	937	612 (33.05)	325 (33.27)	0.46	211 (33.23)	114 (33.33)	0.97
Convulsions	148	98 (5.29)	50 (5.12)	0.93	33 (5.20)	17 (4.97)	0.87
Central Cyanosis	62	39 (2.11)	23 (2.35)	0.34	16 (2.52)	7 (2.05)	0.64
**Malnutrition Status**							
Normal *	1880	1293 (69.82)	587 (60.08)		367 (57.80)	220 (64.33)	
Malnutrition*	517	333 (17.98)	184 (18.83)	< 0.0001	122 (19.21)	62 (18.13)	0.06
Severe malnutrition*	432	226 (12.20)	206 (21.08)		146 (22.99)	60 (17.54)	

*Normal-weight of age z score > -2SD; Malnutrition-weight-for-age z ≤ -2SD and Severe malnutrition-weight-for- age z ≤ -3SD

Table 3 describes four multivariate unconditional logistic regression models to find associates of various abnormal CXR findings. After controlling for age, gender, symptoms of pneumonia, duration of illness, biomass fuel and malnutrition status of cases, statistically significant district-wise heterogeneity remained in the first three models. Model I, which compared abnormal versus normal CXRs, II, which compared CXRs with PEP alone or with other infiltrates versus normal and III, which compared CXRs with PEP alone versus normal, had similar associates for radiological abnormalities whereas Model IV, which compared CXRs with other infiltrates only versus normal, was different. Across all the four models, female gender and those with severe

For peer review only

For peer review only - http://bmjopen.bmj.com/site/about/guidelines.xhtml

malnutrition had statistically significantly higher risk for having abnormal CXRs. A higher risk of radiological abnormalities was also observed in those children with longer duration of illness.

Table 3: Independent Associations between Chest Radiograph findings and demographic and clinical variables among hospitalized children of Community Acquired Pneumonia, using Unconditional Logistic Regression

Variables	Model – I Abnormal/Normal Ref		Model – II PEP alone or with other infiltrates /Normal Ref		Model – III PEP alone / Normal Ref		Model – IV Other infiltrates / Normal Ref	
	Adjusted Odd Ratio (95%CI)	p value	Adjusted Odd Ratio (95%CI)	p value	Adjusted Odd Ratio (95%CI)	p value	Adjusted Odd Ratio (95%CI)	p value
**Districts**								
Lucknow vs. Others	1.58 (1.20-2.10)	<0.0001	2.07 (1.48-2.89)	<0.0001	2.20 (1.52-3.19)	<0.0001	0.98 (0.65-1.47)	0.93
Etawah vs. Others	1.22 (0.88-1.70)	0.23	1.30 (0.87-1.95)	0.19	1.49 (0.95-2.30)	0.07	1.17 (0.74-1.87)	0.50
Patna vs. Others	1.67 (1.27-2.20)	<0.0001	1.89 (1.36-2.64)	<0.0001	2.25 (1.56-3.24)	<0.0001	1.39 (0.95-2.07)	0.09
**Age – Group (months)**								
2-11 Ref								
12-59	0.92 (0.77-1.10)	0.34	0.95 (0.77-1.17)	0.62	1.03 (0.82-1.29)	0.79	0.86 (0.66-1.13)	0.27
**Gender**								
Male Ref								
Female	1.39 (1.16-1.66)	<0.0001	1.34 (1.08-1.65)	0.008	1.28 (1.01-1.61)	0.03	1.48 (1.14-1.92)	0.004
**Symptoms of pneumonia¶**								
Wheezing	0.83 (0.68-1.01)	0.06	0.72 (0.57-0.90)	0.005	0.75 (0.59-0.96)	0.02	1.14 (0.83-1.55)	0.42
Pallor	1.30 (1.08-1.58)	0.006	1.28 (1.03-1.60)	0.02	1.22 (0.95-1.55)	0.12	1.34 (1.01-1.77)	0.04
Vomiting everything	0.90 (0.75-1.09)	0.28	0.80 (0.64-0.99)	0.04	0.78 (0.62-1.01)	0.05	1.09 (0.83-1.08)	0.51
**Duration of illness, fever (days)**	1.06 (1.04-1.09)	<0.0001	1.08 (1.04-1.12)	<0.0001	1.08 (1.04-1.12)	<0.0001	1.03 (0.98-1.48)	0.24
**Biomass fuel**	1.28 (1.05-1.57)	0.02	1.39 (1.10-1.76)	0.006	1.40 (1.14-1.88)	0.003	1.08 (0.79-1.45)	0.64

For peer review only - http://bmjopen.bmj.com/site/about/guidelines.xhtml

**Malnutrition Status**								
Normal *Ref								
Malnutrition*	1.18 (0.93-1.45)	0.15	1.17 (0.91-1.52)	0.23	1.17 (0.88-1.55)	0.27	1.12 (0.82-1.52)	0.47
Severe malnutrition*	1.65 (1.31-2.09)	<0.0001	1.82 (1.34-2.36)	<0.0001	1.87 (1.41-2.47)	<0.0001	1.62 (1.71-2.23)	0.003

Abbreviations: Ref : Reference Category; PEP: Primary End-Point Pneumonia

Footnotes: ¶ No signs of pneumonia taken as a reference
*Normal: weight-for-age z score> -2SD; Malnutrition: weight-for -age z ≤ -2SD; Severe malnutrition: weight-for-age z ≤ -3SD

DISCUSSION

This active, prospective, hospital-based surveillance study was conducted to assess radiological abnormalities in CXRs and to identify the demographic and clinical correlates of specific radiological abnormalities in children aged 2-59 months, hospitalized with WHO defined CAP, residing in pre-specified districts of Uttar Pradesh and Bihar. The study was conducted from January 2015 to April 2017, prior to the introduction of PCV in the national immunization programme of the Government of India.[9]

In our study, among interpretable CXRs, we found that 22.44% (635/2829) cases had PEP alone or with infiltrates, 12.09% (342/2829) had other infiltrates only and 65.46% (1852/2829) had normal findings. Our study used WHO case definition for CAP. [19] A panel of three trained radiologists interpreted CXRs, adopting WHO recommended methodology. [11 12] These make our study methodology robust and results generalizable.

There were 88.54% (2829/3195) interpretable CXRs in the current study. This is similar to 83% (3587/3973) interpretable CXRs reported by Pneumonia Etiology Research for Child Health

For peer review only

For peer review only - http://bmjopen.bmj.com/site/about/guidelines.xhtml

(PERCH) study conducted on 4232 children (1-59 months) in nine sites in seven countries. [27] Consistent with PERCH findings, a vaccine probe trial conducted in Gambia found proportion of interpretable CXRs among unvaccinated cases of pneumonia to be 84.32% (242/287). [28]

There have been several studies in the past two decades, which have reported CXRs findings in hospitalized cases of pediatric CAP. Almost all of these were conducted before the introduction of PCV in their respective regions. A small prospective study conducted in Ethiopia reported radiological abnormality in CXRs in 48.3% (95% CI 39.49-57.22) among 122 children aged 3 months to 14 years with clinically diagnosed WHO-defined severe pneumonia. [29] Similar findings were reported from the Gambian vaccine probe trial where the proportion of radiological abnormality was 45% (95% CI: 43.35-46.46) among unvaccinated hospitalized cases of clinical pneumonia. [28] Likewise, PERCH study found that 54% (95% CI: 52.31-55.57) of CXRs among cases of CAP were abnormal. [27] In all of these studies, proportion of cases with abnormal CXRs is higher than 34.5% (95% CI 32.8-36.3) found by us in the current study. However, our findings are similar to PERCH rural study site of Matlab, Bangladesh that reported radiological abnormality in 35.3% (95% CI: 29.77-40.85) CXRs of hospitalized cases of CAP. [27] Another PERCH urban site of Dhaka, Bangladesh reported 63.10% (95% CI 56.18 -70.02) cases with abnormal CXRs. [27] In our study, radiological abnormalities in CXRs were higher in cases from largely urban districts of Patna and Lucknow compared to rural districts of Darbhanga and Etawah. This is consistent with rural-urban differences in Bangladesh sites of PERCH. [27] Variation in CXR findings among cases of CAP may be due to place of residence, infecting organism, immune response of patient and prior duration of disease.

For peer review only - http://bmjopen.bmj.com/site/about/guidelines.xhtml

In 2016, WHO's Department of Immunization, Vaccines and Biologicals standardized the categorization of radiological pneumonia and established that PEP can be taken as a good surrogate marker of bacterial pneumonia in epidemiological and vaccine efficacy studies.[11] In our study, 22.44 % (95% C.I. 20.90 -23.98), CXRs had PEP alone or with other infiltrates and hence were probably bacterial in etiology. This is similar to findings of PERCH study that reported PEP alone or with other infiltrates in 27% (95% C.I. 25.50 -28.40) hospitalized cases of CAP. [27] However, a study conducted in Gambia reported that 45% (95% CI: 43.35-46.46) non-vaccinated children had PEP and/or other infiltrates [28] which is higher than that found by us or the PERCH study. PEP in CXR has been associated with increased risk of treatment failure ($p=0.002$), increased length of hospitalization ($p=0.0003$) and more days of respiratory support ($p=0.002$) in Botswana when compared with cases reporting` no significant pathology` on CXRs.[30]

In our study, female gender ($p<0.001$) was at the higher risk of developing radiological abnormalities compared to males (**table 3**). The results are in concordance with a hospital-based case-control study carried out in Brazil that reported male gender as a protective factor against pneumonia (OR=0.53; 95%CI 0.39–0.72). [31] However, a study in Mozambique, Africa reported that male gender was not significantly associated with presence of radiological abnormalities (OR =0.77; 95%CI 0.56–1.05) in children (0-59 months) suffering from severe pneumonia. [32] In contrast, a Gambian study reported male preponderance for all pneumonia that was most marked for those whose CXRs showed `other infiltrates/abnormalities`. [28]

For peer review only - http://bmjopen.bmj.com/site/about/guidelines.xhtml

In our study, we observed differential care-seeking by gender for CAP in all four project districts. Although females admitted with CAP were at higher risk of having radiological abnormalities, lower proportions were hospitalized. Gender inequality in health care seeking for females is common in India, as in other South Asian countries. [33] [34] Since there is no health-care financing or provision of health insurance in India, in case of severe illness, parents are less likely to incur out-of-pocket expenditure or incur debts to pay expenses on medical treatment of their daughters as compared to sons. [35] Another Indian study found that male children were five times more likely to be taken early for medical care and three times more likely to be seen by qualified medical doctors compared to female children. [36] We also found that large proportion of hospitalized cases of pneumonia were from urban areas, as there is poor health-care seeking from rural areas. [37]

A systematic review with meta-analysis conducted in 2019 suggests that no one clinical feature is sufficient on its own to diagnose radiological pneumonia. [38] However other socio-demographic and clinical correlates of abnormal CXRs found by us in Model 1 (abnormal versus normal), which increased the risk of radiological abnormalities were presence of pallor, severe malnutrition, longer duration of illness and exposure to biomass fuel. In developing countries exposure to biomass fuel used for cooking has been reported as an important risk factor for CAP.[39] In rural India, majority of the households use biomass fuel like firewood, dung cakes and wood for cooking. [40] Young children are at risk to adverse effects of exposure to biomass fuel as either the households have no separate cooking space or have poor ventilation and sometimes young children stay with their mother while she cooks.

For peer review only - http://bmjopen.bmj.com/site/about/guidelines.xhtml

Other correlates of PEP/radiological pneumonia, which were more likely to be bacterial in etiology, as found in Model II, which compared PEP alone or with other infiltrates versus normal, and Model III, which compared PEP alone versus normal **(table 3)**, besides those found in Model I, were presence of vomiting everything and wheeze on auscultation, both of which were found to be protective. These symptoms/signs are more often reported in viral pneumonia.[41] Correlates of radiological abnormalities of `other infiltrates` (Model IV which compared other infiltrates with normal), which increased the risk, were again female gender, pallor and severe malnutrition. Hence it is difficult to attribute radiological findings of other infiltrates to either bacterial or viral etiology.

Based on our study, almost two-third hospitalized cases of CAP had normal CXRs and could be perhaps of viral etiology. This is supported by a recent study that reported 61.4% (95% CI 57·3–65·6) cases to be viral.[41] One-third of cases of CAP had abnormal CXRs and thus were more likely to be bacterial in etiology.

In India, 13-valent PCV has been introduced in May 2017. A three dose schedule is followed with two primary and one booster, at 6 weeks, 14 weeks and 9 months of age, respectively. PCV 13 provides coverage against 13 serotypes (1, 3, 4, 5, 6A, 6B, 7F, 9V, 14, 18C, 19A, 19F and 23F). [42] Several studies have assessed serotype distribution of pneumococcal disease among children in India. A study conducted in Vellore, India, found that the most common serotypes causing invasive infections among under-five children were 14, 19F, 5, 6A and 6B, all of which are covered by the 13-valent PCV. [43] Another population-based surveillance study conducted in rural Bangladesh found that the most common serotypes of *S. pneumoniae* were 1, 5, 14, 18C

For peer review only - http://bmjopen.bmj.com/site/about/guidelines.xhtml

and 19A and 38 which caused invasive disease and all but one were covered by the 13 valent vaccine. [44] A systematic review and meta-analysis of data collected on Invasive Pneumococcal Disease serotypes from under-five children during the pre-PCV period (between 1980-2007) found that serotypes included in both the 10-valent and 13-valent PCVs accounted for 10 million cases and 600,000 deaths worldwide.[45]

Several strengths of the study are worth-noting. This was an active, prospective, multisite study where recruitments were done from a large hospital surveillance network established especially for the study in four districts of two Indian states that have high under-five mortality rates. Standard WHO definition was used to identify hospitalized cases of CAP. Radiological abnormalities were interpreted by a panel of three trained radiologists at locations out of the surveillance network, blinded to each other as well as clinical features of the case. Despite these strengths, our study findings have certain limitations. First, in our study, pre-existing x-rays machines which were not of uniform specification were used. This might have caused variation in quality of CXR images, though this error was minimized by digitizing the CXR images centrally. Secondly, in our study, clinical data collection was recorded by clinicians in the network hospitals and there could be observer bias. This could also have lead to possibly over reporting of presence of wheezing. In this study, we have not collected information on use of antibiotic prior to hospitalizations; as such information is not available reliably. However, in another study, done in one of the network hospitals of Lucknow in the recent past, it was found that 70.5% children tested positive for antibiotics on urine examnation.[46] Prior use of antibiotics could have possibly lead to under-estimation of radiological pneumonia. We also observed that

For peer review only - http://bmjopen.bmj.com/site/about/guidelines.xhtml

pulse oxymetry was not routinely done in the network hospitals. This could have an impact on the case management but would not have affected the radiological findings of CXRs.

CONCLUSION

Among hospitalized cases of CAP, almost one-third children had abnormal chest radiographs of which about two-thirds had abnormalities related with possible bacterial etiology *(Streptococcus pneumonia)*. Hence, the introduction of pneumococcal vaccination is likely to reduce the burden of childhood pneumonia in India. Since the study was done prior to the introduction of PCV in India, continued surveillance will be required to assess the impact of PCV on radiological findings in cases admitted with CAP. The impact of introduction of PCV in the national immunization programme on under-five mortality rate and burden of CAP needs to be assessed too.

Acknowledgements: We thank the state governments of Uttar Pradesh and Bihar for giving us permission to conduct the study. We extend our gratitude to participating tertiary and secondary care hospitals for their contribution in this study. We offer thanks to Dr. Kamrun Nahar from the International Centre for Diarrhoeal Disease Research, Bangladesh for training the panel of study radiologists. Members of the Lucknow Community-Acquired Pneumonia (CAP) Group: King George's Medical University (KGMU), Lucknow, India: Prof. Shally Awasthi, Department of Pediatrics, Prof. Neera Kohli, Department of Radio-diagnosis, Prof. Monika Agarwal, Department of Community Medicine and Public Health; Mr. Puneet Dhasmana, Project Staff; Patna Medical College and Hospital, Patna, Bihar-Prof. Neelam Verma

For peer review only - http://bmjopen.bmj.com/site/about/guidelines.xhtml

and Prof. Chandra Bhushan Kumar, Department of Pediatrics; Darbhanga Medical College and Hospital, Darbhanga, Bihar, India- Prof. Chittaranjan Roy, Department of Community Medicine, Prof. Kripanath Mishra, Department of Pediatrics; Uttar Pradesh University of Medical Sciences, Etawah, Uttar Pradesh, India- Prof. Pankaj Kumar Jain, Department of Community Medicine, Prof. Rajesh Yadav, Department of Pediatrics.

Contributors: The study was conceived and designed by SA. CAP study group performed data acquisition.CMP and NM[1]conducted the statistical analysis of the data. The paper was written by SA, TR, MA and CMP. AC, NM[3], RCS and NK interpreted chest x-rays. All authors were involved in drafting and revising the work and approved final submission.

Funding statement: The study was supported by Bill & Melinda Gates Foundation (https://www.gatesfoundation.org/) via Grant No: OPP1118005**.** Funding agency had no role in the design of study and collection, analysis and interpretation of data, or in writing the manuscript.

Competing interests: None declared.

Patient consent for publication: Not required.

Ethics approval: The Institutional Ethics Committee of King George's Medical University (Lucknow), The Uttar Pradesh University of Medical Sciences (Etawah), Patna Medical College and Hospital (Patna) and Darbhanga Medical College and Hospital (Darbhanga) gave ethical approval for conduct of study.

Provenance and peer review: Not commissioned; externally peer reviewed.

Data sharing statement: The data contained within this study can be obtained by writing to shally07@gmail.com

For peer review only

For peer review only - http://bmjopen.bmj.com/site/about/guidelines.xhtml

Copyright information: This is an Open Access article distributed in accordance with the Creative Commons Attribution Non Commercial (CC BY-NC 4.0) license, which permits others to distribute, remix, adapt, build upon this work non-commercially, and license their derivative works on different terms, provided the original work is properly cited and the use is non-commercial. See: http://creativecommons.org/licenses/by-nc/4.0/

Supporting Information

S1 Appendix: Chest radiograph interpretation form

REFERENCES:

1. UNICEF U. Levels and trends in child mortality. Estimates developed by the UN inter-agency group for child mortality estimation. 2011.
2. Amouzou A, Velez LC, Tarekegn H, *et al*. One is too many: ending child deaths from pneumonia and diarrhoea. New York: Unicef, 2016. pp 73–75
3. Mathew JL, Patwari AK, Gupta P, *et al*. Acute respiratory infection and pneumonia in India: a systematic review of literature for advocacy and action: UNICEF-PHFI series on newborn and child health, India. *Indian Pediatr*2011; 48: 191–218.
4. Farooqui H, Jit M, Heymann DL, *et al*. Burden of severe pneumonia, pneumococcal pneumonia and pneumonia deaths in Indian states: modelling based estimates. *PLoS One* 2015 ; 10(6).

For peer review only - http://bmjopen.bmj.com/site/about/guidelines.xhtml

5. Rudan I, O'brien KL, Nair H, *et al*. Epidemiology and etiology of childhood pneumonia in 2010: estimates of incidence, severe morbidity, mortality, underlying risk factors and causative pathogens for 192 countries. *Journal of global health*. 2013 Jun; 3(1).

6. Leung DT, Chisti MJ, Pavia AT. Prevention and control of childhood pneumonia and diarrhea. *Pediatric Clinics*. 2016 Feb 1;63(1):67-79.

7. Virkki R, Juven T, Rikalainen H, *et al*. Differentiation of bacterial and viral pneumonia in children. *Thorax*. 2002 May 1;57(5):438-41.

8. World Health Organization. Pneumococcal conjugate vaccines in infants and children under 5 years of age: WHO position paper–February 2019. [Accessed 12 July 2019]

9. Government of India. Introduction of Pneumococcal Conjugate Vaccine: Operational Guidelines. Ministry of Health and Family Welfare, 2017 Available: https://nhm.gov.in/New_Updates_2018/NHM_Components/Immunization/Guildelines_for_immunization/Operational_Guidelines_for_PCV_introduction.pdf. [Accessed 12 July 2019]

10. Cherian T, Mulholland EK, Carlin JB, *et al*. Standardized interpretation of paediatric chest radiographs for the diagnosis of pneumonia in epidemiological studies. *Bulletin of the World Health Organization*. 2005;83:353-9

11. Pneumonia Vaccine Trial Investigators' Group. Standardization of Interpretation of Chest Radiographs for the Diagnosis of Pneumonia in Children. Geneva: Department of Vaccines and Biologicals, World Health Organization; 2001

12. Mahomed N, Fancourt N, De Campo J, *et al*. Preliminary report from the World Health Organization chest radiography in epidemiological studies project. *Pediatric radiology*. 2017 Oct 1;47(11):1399-404.

For peer review only - http://bmjopen.bmj.com/site/about/guidelines.xhtml

13. Greenberg D, Givon-Lavi N, Ben-Shimol S,*et al*. Impact of PCV7/PCV13 introduction on community-acquired alveolar pneumonia in children< 5 years. *Vaccine*. 2015 Aug 26;33(36):4623-9. 6.

14. Sutcliffe CG, Shet A, Varghese R, *et al*. Nasopharyngeal carriage of Streptococcus pneumoniae serotypes among children in India prior to the introduction of pneumococcal conjugate vaccines: a cross-sectional study. *BMC Infect Dis*. 2019 Dec;19(1):605.

15. Annual health survey (AHS) factsheet 2012–13 Uttar Pradesh [Internet]. New Delhi: Office of Registrar General & Census Commissioner, Ministry of Home Affairs, Government of India; 2010–2011. Available:http://www.censusindia.gov.in/vital_statistics/AHSBulletins/AHS_Factsheets_2012-13/FACTSHEET-UTTAR_PRADESH.pdf. [Accessed 5 August 2019]

16. Annual health survey (AHS) factsheet 2012–13 Bihar [Internet]. New Delhi: Office of Registrar General & Census Commissioner, Ministry of Home Affairs, Government of India; 2010–2011. Available:http://www.censusindia.gov.in/vital_statistics/AHSBulletins/AHS_Factsheets_2012-13/FACTSHEET-Bihar.pdf. [Accessed 5 August 2019].

17. Awasthi S, Pandey CM, Verma T, *et al*. Incidence of community-acquired pneumonia in children aged 2-59 months of age in Uttar Pradesh and Bihar, India, in 2016: An indirect estimation. *PloS one*. 2019 Mar 20;14(3):e0214086.

18. Awasthi S, Singh JV, Kohli N, *et al*. Hospital-based surveillance for radiological pneumonia in children under 5 years of age in Uttar Pradesh and Bihar. *Pediatric Infectious Disease*. 2016 Jun 30;8(2):52-7.

For peer review only

For peer review only - http://bmjopen.bmj.com/site/about/guidelines.xhtml

19. World Health Organization. Pocket Book of Hospital Care for Children: Guidelines for the Management of Common Childhood Illnesses. 2nd edn. Geneva, Switzerland: World Health Organization, 2013.

20. Charan J, Biswas T. How to calculate sample size for different study designs in medical research?. Indian journal of psychological medicine. 2013 Apr;35(2):121.

21. Integrated Management of Childhood Illness (IMCI) Chart Booklet [online] Geneva: World Health Organization. Available: https://apps.who.int/iris/bitstream/handle/10665/104772/9789241506823_Chartbook_eng.pdf [Accessed19 December 2019].

22. Bassignani MJ, Bubash-Faust L, Ciambotti J, *et al*. Conversion of teaching file cases from film to digital format: a comparison between use of a diagnostic-quality digitizer and use of a flatbed scanner with transparency adapter. *Acad Radiol*. 2003 May 1;10(5):536-42.

23. World Health Organization. The WHO Anthro Survey Analyser. In: World Health Organization [Internet]. Available: https://www.who.int/nutgrowthdb/software/en/. [Accessed: 10 June 2019].

24. Horton NJ, Kleinman KP. Much ado about nothing: A comparison of missing data methods and software to fit incomplete data regression models. The American Statistician. 2007 Feb 1;61(1):79-90.

25. SPSS Inc. Released 2009. SPSS for Windows [software], Version 22·0. Chicago, SPSS Inc. Available from:www.spss.com

26. von Elm E, Altman DG, Egger M, et al. The strengthening the reporting of observational studies in epidemiology (STROBE) statement: guidelines for reporting observational studies. J Clin Epidemiol 2008;61:344–9

For peer review only - http://bmjopen.bmj.com/site/about/guidelines.xhtml

27. Fancourt N, Deloria Knoll M, Baggett HC, *et al*. Chest radiograph findings in childhood pneumonia cases from the multisite PERCH study. *Clin Infect Dis*. 2017 May 29;64(suppl_3):S262-70

28. Enwere G, Cheung YB, Zaman SM, *et al*. Epidemiology and clinical features of pneumonia according to radiographic findings in Gambian children. *Trop Med Int Health*. 2007 Nov;12(11):1377-85.

29. Meiron Hassen, Alemayehu Toma, Mulugeta Tesfay, *et al*. Radiologic Diagnosis and Hospitalization among Children with Severe Community Acquired Pneumonia: A Prospective Cohort Study. *Biomed Res Int*, vol. 2019, Article ID 6202405, 8 pages, 2019. https://doi.org/10.1155/2019/6202405.

30. Kelly MS, Crotty EJ, Rattan MS, *et al*. Chest radiographic findings and outcomes of pneumonia among children in Botswana. *Pediatr Infect Dis J*. 2016 Mar;35(3):257.

31. da Fonseca Lima EJ, Mello MJ, Lopes MI, *et al*. Risk factors for community-acquired pneumonia in children under five years of age in the post-pneumococcal conjugate vaccine era in Brazil: a case control study. *BMC Pediatr*. 2016 Dec;16(1):157.

32. Sigauque B, Roca A, Bassat Q, *et al*. Severe pneumonia in Mozambican young children: clinical and radiological characteristics and risk factors. *J Trop Pediatr*. 2009 Apr 28;55(6):379-87.

33. Ismail SA, McCullough A, Guo S, *et al*. Gender-related differences in care-seeking behaviour for newborns: a systematic review of the evidence in South Asia. BMJ global health. 2019 May 1;4(3):e001309.

34. Fikree FF, Pasha O. Role of gender in health disparity: the South Asian context. BMJ 2004;328:823–6. 10.1136/bmj.328.7443.823

For peer review only - http://bmjopen.bmj.com/site/about/guidelines.xhtml

35. Asfaw A, Lamanna F, Klasen S. Gender gap in parents' financing strategy for hospitalization of their children: evidence from India. Health economics. 2010 Mar;19(3):265-79.

36. Pandey A, Sengupta PG, Mondal SK, *et al*. Gender differences in healthcare-seeking during common illnesses in a rural community of West Bengal, India. Journal of Health, Population and Nutrition. 2002 Dec 1:306-11.

37. Jana A, Basu R. Examining the changing health care seeking behavior in the era of health sector reforms in India: evidences from the National Sample Surveys 2004 & 2014. Global health research and policy. 2017 Dec;2(1):6.

38. Rambaud-Althaus C, Althaus F, Genton B, *et al*. Clinical features for diagnosis of pneumonia in children younger than 5 years: a systematic review and meta-analysis. *Lancet Infect Dis*. 2015 Apr 1;15(4):439-50

39. Awasthi S, Glick AH, Fletcher RH. Effect of cooking fuels on Respiratory Diseases in Pre-school children in Lucknow, India. *Am J Trop Med Hyg* 1996; 55: 48-51

40. Tielsch JM, Katz J, Thulasiraj RD, *et al*. Exposure to indoor biomass fuel and tobacco smoke and risk of adverse reproductive outcomes, mortality, respiratory morbidity and growth among newborn infants in south India. *Int J Epidemiol*. 2009 Sep 16;38(5):1351-63.

41. O'Brien KL, Baggett HC, Brooks WA, *et al*. Causes of severe pneumonia requiring hospital admission in children without HIV infection from Africa and Asia: the PERCH multi-country case-control study. *The Lancet*. 2019 Jun 27

42. World Health Organization. Pneumococcal conjugate vaccines in infants and children under 5 years of age: WHO position paper–February 2019. Accessed 12th March. 2019.

For peer review only - http://bmjopen.bmj.com/site/about/guidelines.xhtml

43. Balaji V, Jayaraman R, Verghese VP, *et al*. Pneumococcal serotypes associated with invasive disease in under five children in India & implications for vaccine policy. The Indian journal of medical research. 2015 Sep;142(3):286.

44. Arifeen SE, Saha SK, Rahman S, *et al*. Invasive pneumococcal disease among children in rural Bangladesh: results from a population-based surveillance. Clinical Infectious Diseases. 2009 Mar 1;48 (Supplement_2):S103-13.

45. Johnson HL, Deloria-Knoll M, Levine OS, *et al*. Systematic evaluation of serotypes causing invasive pneumococcal disease among children under five: the pneumococcal global serotype project. PLoS medicine. 2010 Oct 5;7(10):e1000348

46. Bénet T, Picot VS, Awasthi S, *et al*. Severity of pneumonia in under 5-year-old children from developing countries: a multicenter, prospective, observational study. The American journal of tropical medicine and hygiene. 2017 Jul 12;97(1):68-76

Figure Legend

Figure 1: Flow diagram of cases of community acquired pneumonia recruited from participating districts before the introduction of pneumococcal conjugate vaccine (January 2015-April 2017)

For peer review only

For peer review only - http://bmjopen.bmj.com/site/about/guidelines.xhtml

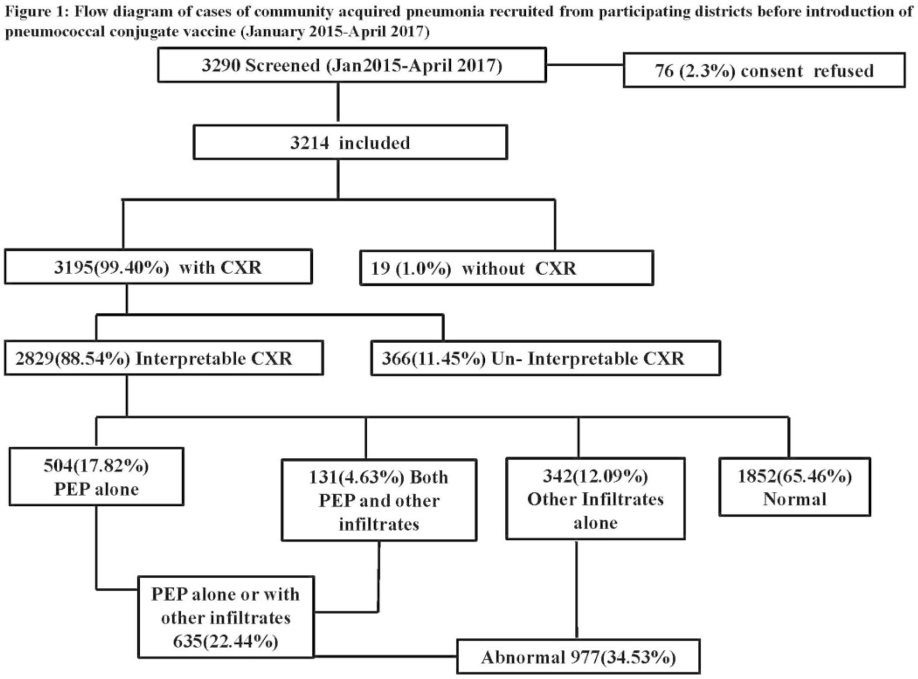

Figure 1: Flow diagram of cases of community acquired pneumonia recruited from participating districts before the introduction of pneumococcal conjugate vaccine (January 2015-April 2017)

254x190mm (300 x 300 DPI)

Childhood Pneumonia Surveillance

Department of Paediatrics, KGMU, Lucknow ,UP

Form-R

(RADIOLOGY REPORT FORM)

1	**Drs_ID**	[__][__][__][__]
	**Radiology Report**	**Patient Details**
2	**IDNo:**	[__] / [__] / [__][__][__]/[__][__][__][__]<br>State /District / Unit / Subject number<br>*(For office use)*
2	Date Of Report	[__][__]/[__][__]/[__][__][__][__] (DD/MM/YYYY)
	**Report Details**	**Findings (tick one)**
3	Image Quality	Adequate ☐ Suboptimal ☐ Un-interpretable ☐
4	Significant Pathology	Yes ☐ No ☐ Un-interpretable ☐
5	**End Point Consolidation**	
5a	Left	Yes ☐ No ☐ Un-interpretable ☐
5b	Right	Yes ☐ No ☐ Un-interpretable ☐<br>Uninterpretable
6	Other Infiltrates/Abnormalities	
6a	Left	Yes ☐ No ☐ Un-interpretable ☐
6b	Right	Yes ☐ No ☐ Un-interpretable ☐
7	Pleural Fluid	
7a	Left	Yes ☐ No ☐ Un-interpretable ☐
7b	Right	Yes ☐ No ☐ Un-interpretable ☐
8	Comments:	[ ]
9	Conclusion:	a) Primary endpoint pneumonia only ☐<br>b) Other infiltrate only ☐<br>c) Both PEP and other infiltrate ☐<br>d) Normal ☐<br>e) Un-interpretable for any findings☐

y only

STROBE Statement—Checklist of items that should be included in reports of ***cross-sectional studies***

	Item No	Recommendation	Page Number
**Title and abstract**	1	(*a*) Indicate the study's design with a commonly used term in the title or the abstract	1
		(*b*) Provide in the abstract an informative and balanced summary of what was done and what was found	2-3
**Introduction**			
Background/rationale	2	Explain the scientific background and rationale for the investigation being reported	4
Objectives	3	State specific objectives, including any pre-specified hypotheses	5
**Methods**			
Study design	4	Present key elements of study design early in the paper	5
Setting	5	Describe the setting, locations, and relevant dates, including periods of recruitment, exposure, follow-up, and data collection	5
Participants	6	(*a*) Give the eligibility criteria, and the sources and methods of selection of participants	5-6
Variables	7	Clearly define all outcomes, exposures, predictors, potential confounders, and effect modifiers. Give diagnostic criteria, if applicable	5-6
Data sources/ measurement	8*	For each variable of interest, give sources of data and details of methods of assessment (measurement). Describe comparability of assessment methods if there is more than one group	7-8
Bias	9	Describe any efforts to address potential sources of bias	25
Study size	10	Explain how the study size was arrived at	6
Quantitative variables	11	Explain how quantitative variables were handled in the analyses. If applicable, describe which groupings were chosen and why	11-13
Statistical methods	12	(*a*) Describe all statistical methods, including those used to control for confounding	11-13
		(*b*) Describe any methods used to examine subgroups and interactions	Table 1, 2 &3
		(*c*) Explain how missing data were addressed	12 reference 24
		(*d*) If applicable, describe analytical methods taking account of sampling strategy	11-13
		(*e*) Describe any sensitivity analyses	NA
**Results**			
Participants	13*	(a) Report numbers of individuals at each stage of study—eg numbers potentially eligible, examined for eligibility, confirmed eligible, included in the study, completing follow-up, and analysed	14
		(b) Give reasons for non-participation at each stage	Figure 1
		(c) Consider use of a flow diagram	Figure 1
Descriptive data	14*	(a) Give characteristics of study participants (eg demographic, clinical, social) and information on exposures and potential confounders	14-15 Table 1
		(b) Indicate number of participants with missing data for each variable of interest	Figure 1

For peer review only

Outcome data	15*	Report numbers of outcome events or summary measures	
Main results	16	(*a*) Give unadjusted estimates and, if applicable, confounder-adjusted estimates and their precision (eg, 95% confidence interval). Make clear which confounders were adjusted for and why they were included	Table 3 (adjusted odds ratio)
		(*b*) Report category boundaries when continuous variables were categorized	Table 3
		(*c*) If relevant, consider translating estimates of relative risk into absolute risk for a meaningful time period	NA (calculated only odds ratio)
Other analyses	17	Report other analyses done—eg analyses of subgroups and interactions, and sensitivity analyses	Table 3
**Discussion**			
Key results	18	Summarise key results with reference to study objectives	21-23
Limitations	19	Discuss limitations of the study, taking into account sources of potential bias or imprecision. Discuss both direction and magnitude of any potential bias	25-26
Interpretation	20	Give a cautious overall interpretation of results considering objectives, limitations, multiplicity of analyses, results from similar studies, and other relevant evidence	21-23
Generalisability	21	Discuss the generalisability (external validity) of the study results	21
**Other information**			
Funding	22	Give the source of funding and the role of the funders for the present study and, if applicable, for the original study on which the present article is based	27

*Give information separately for exposed and unexposed groups.

Note: An Explanation and Elaboration article discusses each checklist item and gives methodological background and published examples of transparent reporting. The STROBE checklist is best used in conjunction with this article (freely available on the Web sites of PLoS Medicine at http://www.plosmedicine.org/, Annals of Internal Medicine at http://www.annals.org/, and Epidemiology at http://www.epidem.com/). Information on the STROBE Initiative is available at www.strobe-statement.org.

For peer review only

For peer review only - http://bmjopen.bmj.com/site/about/guidelines.xhtml
